# Genetic correlation-guided mega-analysis of DO mice provides mechanistic insight and candidate genes for age-related pathologies

**DOI:** 10.1371/journal.pgen.1012037

**Published:** 2026-02-27

**Authors:** Martin N. Mullis, Austin E. Y. T. Lefebvre, Kathyayini Sivasubramanian, Angela Luo, Florian Schmid, Matt Sooknah, Kevin M. Wright, Anil Raj, José Zavala-Solorio, Chunlian Zhang, Johannes Riegler, Astrid Gillich, J. Graham Ruby

**Affiliations:** Calico Life Sciences LLC, South San Francisco, California, United States of America; Newcastle University, UNITED KINGDOM OF GREAT BRITAIN AND NORTHERN IRELAND

## Abstract

Diversity Outbred (DO) mice are a powerful model system for mapping complex traits due to their high genetic diversity and mapping resolution. However, while there are extensive tools available for standard genetic analysis in DO mice, fewer techniques have been implemented to facilitate integrated, cross-study analysis. Here, we implement Haseman-Elston regression to estimate genetic correlations among 7,233 phenotypes measured across eleven independent DO mouse studies. We used this network of genetic correlations to cluster phenotypes according to shared genetics, which enhanced the power to detect quantitative trait loci (QTL). This approach empowered the detection of 884 QTL for 383 meta-phenotypes, explaining an average of 40.36% of the total genetic variance per mega-analysis. We leveraged this network for insights into specific areas of biology, including lifespan, frailty, immune composition, histological and functional lung phenotypes, and histological phenotypes of the aorta. We found the genetics of lifespan to share limited correlation with the genetics of frailty but stronger correlation with the genetics of immune cell composition. Additionally, mega-analyses driven by genetic correlations identified candidate genes (e.g., *Cdkn2b*) associated with degraded extracellular matrix in the aorta. Finally, an ensemble of genetic analyses implicated pulmonary neuroendocrine cell signaling and/or differentiation as a key driver of multiple lung pathophenotypes.

## Author summary

Diversity Outbred (DO) mice are a powerful system for studying the genetics of complex traits, but many of the techniques that are useful for human genetic studies have not been adapted for use with them. Here, we implement a method for DO’s – genetic correlation – that estimates whether pairs of traits are influenced by the same genetic factors. This technique allows traits with similar underlying biology to be identified, even when data on the traits was collected in different cohorts of animals. By applying this method to thousands of traits from different datasets, we gained insight into how seemingly disparate aspects of physiology are connected. We also identified ensembles of traits whose genetics can be analyzed together, empowering the discovery of shared genetic factors. We applied this technique to discover genes that influence the health of elastic tissues: aortas and lungs. We also provide the tools and resources to gain similar insights from the wider scope of DO mouse research studies, both past and future.

## Introduction

Diversity Outbred (DO) mice are an outbred mouse population consisting of individuals derived from the Collaborative Cross (CC) and are a powerful model system for mapping complex traits [1]. The inbred founding strains are genetically diverse and high levels of heterozygosity can lead to sub-megabase (Mb) mapping resolution [1–3]. Cohorts of genetically unique individuals also provide an intervention testing platform in which strain background effects can be avoided [4].

Versus human cohorts, DO mice provide the typical advantages of in vivo biology, including enhanced opportunities for experimental manipulation. However, salient to the application of genetic analyses, mouse experiments typically use smaller numbers of individuals than human cohort studies, limiting their power. Further, while data are typically shared amongst the DO research community [5], a similar ecosystem of analytical tools that facilitate cross-study analyses is lacking. The DO platform would benefit from implementation of methodologies to enable comparative analysis between independent studies. Ideally, these approaches could be used to inform genetic meta-analysis of phenotypes within and across studies, empowering the detection of quantitative trait loci (QTL) influencing phenotypes of interest.

Genetic correlation (*r*_*g*_) is one such methodology, which measures the proportion of genetic variance that is shared between two traits due to common underlying genetic effectors [6]. This relationship can be useful for discovering shared biological mechanisms or pathways, particularly when the focal traits are genetically complex. Importantly, genetic correlations can be estimated for pairs of traits measured in different cohorts, facilitating cross-study genetic analyses, and can provide insight into gene-by-gene (*GxG*) or gene-by-environment (*GxE*) interactions through the measurement of a single phenotype in different populations, environments, or treatment groups. Multiple methods for *r*_*g*_ estimation are described, but only a residual maximum likelihood (REML)-based method has been implemented for DO mice [7,8]. This implementation requires the compared traits to be measured in a common set of animals, limiting it to within-study applications (e.g. [77]). Another method, linkage disequilibrium score regression (LDSC), can be performed using meta-statistics and is commonly applied to human GWAS; however, this method provides biased heritability and genetic correlation estimates within admixed populations with limited recombination [9,10].

Haseman-Elston regression is a method originally developed for the estimation of trait heritability (*h*^*2*^) and involves regression of phenotypic similarity against kinship across pairs of related individuals [11]. Heritability and genetic correlation are linked concepts (see Methods), implying that Haseman-Elston can be extended to compute *r*_*g*_ for any pair of phenotypes. Its reliance on only between-animal comparisons, independence from a population’s haplotype structure, and straight-forward calculation all evince appropriateness for the estimation of *r*_*g*_ between traits measured in independent cohorts of DO mice.

We implemented Haseman-Elston for DO mice and used it to estimate *h*^*2*^ and *r*_*g*_ across 7,233 phenotypes spanning 11 independent studies. These include traits such as lifespan, frailty, immune cell composition, and newly-reported histological phenotypes in the lung and aorta. Intra-trait *r*_*g*_ estimates between independent longitudinal measurements or intervention groups revealed that the genetic architectures of many complex traits in DO mice are more consistent between age groups than across dietary interventions. Phenotypes were clustered according to *r*_*g*_ patterns across the dataset, then aggregated for QTL discovery via mega-analysis. In total, 884 QTL were detected across 383 meta-phenotypes, with statistically-significant QTL explaining an average of 40.36% of the meta-traits’ heritable phenotypic variances. Patterns of genetic correlations among clusters of traits and lifespan revealed a significant overlap in genetic architecture governing immune cell composition and longevity, while measurements captured by frailty assays were less genetically correlated with lifespan. Finally, *r*_*g*_-guided meta-analyses of aorta- and lung-derived phenotypes identified candidate genes associated with degradation of the extracellular matrix in the aorta and inflammation and/or tumorigenesis in the lungs.

## Results

### Implementation of Haseman-Elston regression to estimate the heritabilities and genetic correlations of DO mouse traits

**Fig 1:**
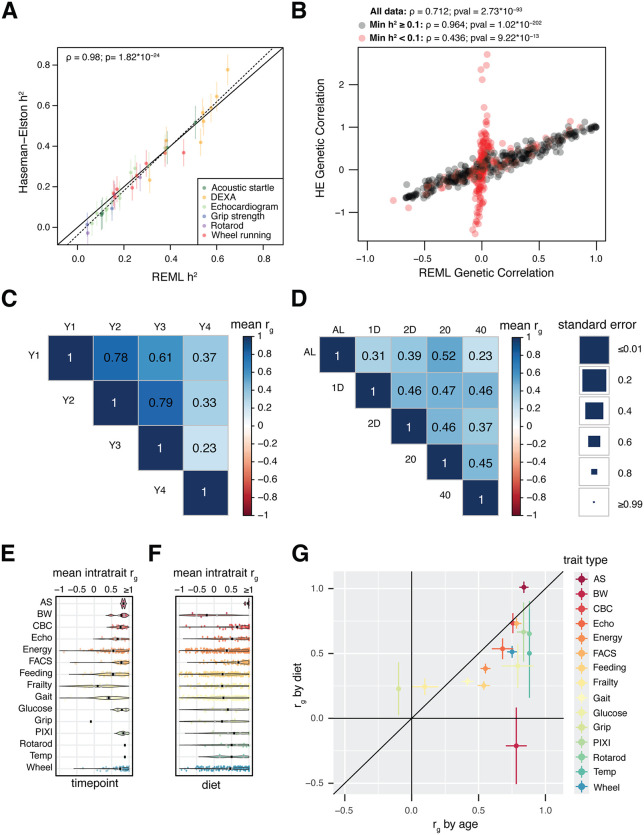
Performance of Haseman-Elston regression implementation and estimation of *r_g_* among traits measured across diet and time point. A, Comparison of *h^2^* estimates for 36 DO mouse traits derived from our implementation of Haseman-Elston regression to those from an orthogonal method, Residual Maximum Likelihood (REML). The line of identity is denoted by a solid line; the best-fit line is denoted by a dashed line. The Pearson correlation coefficient and accompanying p-value are reported in the upper-left of the plot area. Traits are colored by type: ‘AS’ - Acoustic Startle. ‘DX’ - DEXA scan. ‘EC’ - Echocardiogram. ‘GS’ - Grip Strength. ‘RR’ - Rotarod. ‘WR’ - Wheel Running. B, Comparison of r_g_ estimates among all pairs of 36 DO mouse traits derived from Haseman-Elston regression to those from REML. C, Mean intratrait *r*_*g,t*_ across time points for individual traits measured in the DRiDO study. Black bars denote the average mean intratrait r_g_ for traits of a particular type, and gray bars represent the mean intratrait *r_g_* + /- the standard error of the mean. ‘AS’ - acoustic startle. ‘BW’ - body weight. ‘CBC’ - complete blood count. ‘FACS’ - immune cell composition via fluorescence activated cell sorting. ‘PIXI’ - body composition (lean, fat, bone density). D, Mean intratrait *r*_*g,d*_ dietary groups for individual traits measured in the DRiDO study. E, Mean intratrait r_g_ across diet and time point for each category of trait, with accompanying standard errors. F, Mean genetic correlations among traits measured at different time points in the Dietary Restriction in DO Mice (DRiDO) study. Size of the squares are inversely proportional to the standard error of each mean genetic correlation (*left*). G, Mean genetic correlations among traits measured in different dietary groups in the DRiDO study.

We sought to utilize genetic correlations (*r*_*g*_) to guide mega-analysis of phenotypes from DO mouse populations by identifying groups of traits with shared genetic components. To this end, we implemented an updated form of Haseman-Elston regression [12] in which the vectorized outer product of z-score normalized pairwise trait values (a measure of phenotypic similarity) are regressed on pairwise relatedness to estimate the narrow-sense heritability (*h*^2^) for traits (S1A-C Fig and S1 Table and Data D1-D2 available at https://doi.org/10.5061/dryad.sj3tx96gd), **Methods**) [13]. This implementation was tested on simulated phenotypes and produced accurate *h*^*2*^ estimates across a range of *h*^*2*^ values and sample sizes, with precision increasing as a function of the sample size (S1D Fig, **Methods**). It was further evaluated using real data by comparing *h*^*2*^ estimates it produced to those generated by the REML method [8]. Haseman-Elston and REML produced highly correlated *h*^2^ estimates across 36 traits from that reflected diverse physiology (Fig 1A; ρ = 0.98; p = 1.82x10^-24^). Previous comparisons of Haseman-Elston and REML have yielded similarly correlated results [14]. Our implementation also produced *h*^*2*^ estimates that were highly correlated with a previous implementation of Haseman-Elston regression (S2 Fig) [15].

Initial calculation of the standard error (SE) for Haseman-Elston estimates of *h*^2^, using Fisher’s techniques for calculation of the standard error of a regression coefficient [11,16,17], failed to accurately reflect the variances obtained from subset analyses or repeated simulations, becoming less accurate as true *h*^2^ increased. A potential theoretical basis for the discrepancy in our own standard error estimates is that the independence of phenotypic measurements decreases as a function of both the trait *h*^*2*^ and the relatedness of individuals, impacting the number of effective individuals in the analysis; to address this, we derived an empirical ad hoc method for SE calculation, discussed in detail in **Methods**.

As in previous work extending Haseman-Elston regression to genetic correlations [17], we applied our implementation to pairs of traits (i.e., genetic correlation *r*_*g*_) via a structural equation model (S1C Fig), allowing the estimation of *r*_*g*_ provided there are positive *h*^*2*^ estimates for both traits. Haseman-Elston regression was used to estimate all 630 pairwise genetic correlations among the 36 phenotypes from [8], and those values were compared to *r*_*g*_ estimates produced by REML. Estimates produced by these two methods were positively correlated (Fig 1B; ρ = 0.71; p = 2.73x10^-93^). However, Haseman-Elston produced exaggerated *r*_*g*_ estimates for pairs of traits with low *r*_*g*_ according to REML. Differences between the two methods were inversely proportional to the *h*^2^ of the traits used in the *r*_*g*_ estimates (S3A-B Fig), with REML estimates trending towards zero in low-heritability cases. We hypothesized that the extreme values produced by Haseman-Elston regression were driven by low statistical power, either due to small sample sizes (n) or low *h*^2^ of the traits being compared. Because sample size was relatively large across the 36 traits examined (min = 646, max = 900, mean = 854), we focused on the impact of one or more traits having low *h*^2^. Indeed, the standard error of HE regression estimates increased as either the minimum or mean *h*^2^ of the pair of traits decreased (S3C-D Fig). Haseman-Elston and REML estimates were more strongly correlated when both traits under consideration had *h*^*2*^ ≥ 10% (Fig 1B, black points; (ρ = 0.96; p = 1.02x10^-202^), while estimates were more weakly correlated for trait pairs involving one traits with *h*^*2*^ < 10% (ρ = 0.44; p = 9.22x10^-13^). We therefore imposed a heritability threshold of *h^2^* ≥ 10% for all downstream analysis, unless specified.

### The genetic bases of many complex traits were more sensitive to diet than age

A recent study of Dietary Restriction in Diversity Outbred mice (DRiDO) [18] included 429 diverse traits with *h*^2^ ≥ 10% (S1 Table). All of these traits were measured in mice on one of five diets: an *ad libitum* diet (‘AL’), a one day intermittent fasting diet (‘1D’), a two day intermittent fasting diet (‘2D’), 20% reduced calorie diet (‘20CR’), or a 40% reduced calorie diet (‘40CR’). Of these traits, 415 had been measured longitudinally (either annually or bi-annually, with at least two measurements across the study) and provided an opportunity to assess the degree to which age or diet modifies the genetic architecture of heritable traits in DO mice.

For each of the 415 traits with annual measurements, *r*_*g*_ was estimated combinatorially between all time points (Fig 1C), and also combinatorially between all diet groups for each yearly measurement (Fig 1D). When averaged across all traits, mean intra-trait *r*_*g*_ estimates (*r¯*_*g*_) were the highest at younger timepoints, with a marked decrease late in an animal’s lifespan (year 4). With the exception of year 4 data, intra-trait *r¯*_*g*_ estimates were consistently lower among diet groups than time points.

To explore whether these patterns were consistent across diverse sets of traits, the six longitudinal intra-trait *r*_*g*_ values and ten dietary intra-trait *r*_*g*_ values were separately averaged within each individual trait, resulting in mean timepoint intra-trait values (*r̄*_*g,t*_; Fig 1E) and mean dietary intra-trait values (*r̄*_*g,d*_; Fig 1F). The average *r̄*_*g,t*_ across the traits was 0.591, revealing substantially shared genetics for traits across ages. The genetics of rotarod (*r̄*_*g,t*_ = 0.880), body temperature (‘Temp’; *r̄*_*g,t*_ = 0.880), and acoustic startle (‘AS’; *r̄*_*g,t*_ = 0.839) were the most stable with age; and the genetics of gait (*r̄*_*g,t*_ = 0.417), frailty (*r̄*_*g,t*_ = 0.098), and grip strength (‘Grip’; mean *r̄*_*g,t*_ = -0.098) were the least stable. The average *r¯*_*g,d*_ across traits was 0.390 (Fig 1F), i.e. the genetics of phenotypes measured in that study interacted more with dietary interventions (*GxE*) than with age (*GxAge)*. The genetics of acoustic startle (*r¯*_*g,d*_ = 1.010), hematology (‘CBC’; *r¯*_*g,d*_ = 0.733), and immune cell composition (‘FACS’; *r¯*_*g,d*_ = 0.731) were the most stable across diet and the genetics of frailty (*r¯*_*g,d*_ = 0.244), grip strength (*r¯*_*g,d*_ = 0.228), and body weight (‘BW’; *r¯*_*g,d*_ = -0.212) were the least stable. Across all of the traits measured in the DRiDO study, the only categories in which the genetics were more responsive to age than diet were acoustic startle, grip strength, and frailty (Fig 1G).

### Constructing an atlas of genetic correlations across DO mouse studies

**Fig 2:**
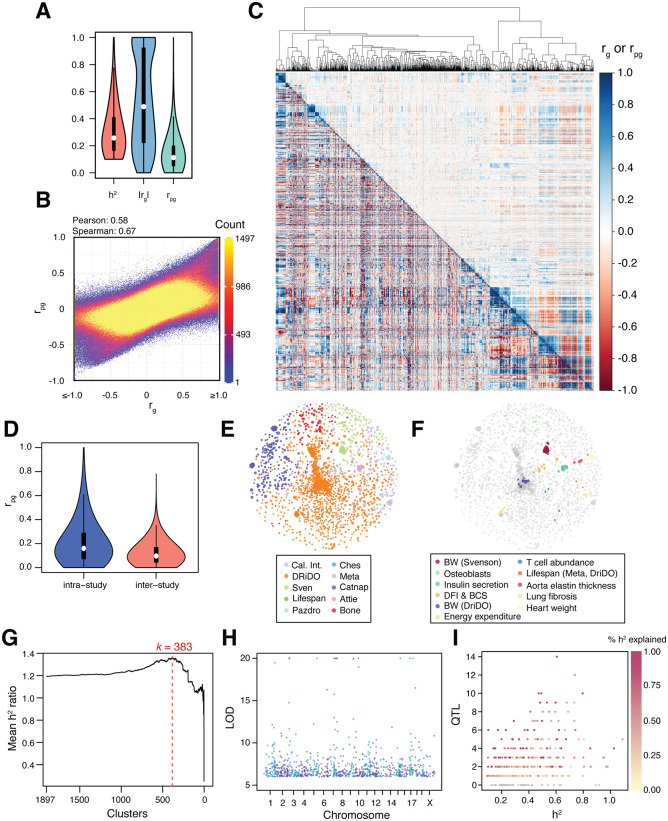
An atlas of *r_g_* and *r_pg_* estimates among complex phenotypes in DO mice. A, The distribution of *h^2^*, *r_g_*, and *r_pg_*, estimates for DO mouse phenotypes used in downstream analysis. The median, 25^th^ percentile, and 75^th^ percentile of the distribution are denoted via box and whisker plots. B, The relationship between the pairwise genetic correlation (*r_g_*) and Pearson correlation of genetic correlations (*r_pg_*) for each pair of traits in the dataset. Pearson and Spearman correlation coefficients are reported. C, A hierarchically clustered heatmap of *r_g_* (*lower triangle*) and *r_pg_* (*upper triangle*) estimates among 1,898 DO phenotypes. Phenotypes measured in individual dietary groups from the DRiDO study are excluded. The accompanying dendrogram shows euclidean distances among traits based on their *r_pg_* estimates. D, The distribution of *r*_*pg*_ values among traits measured in the same study (intra-study, blue) or in different studies (inter-study, red). The median, 25^th^ percentile, and 75^th^ percentile of the distribution are denoted via box and whisker plots. E-F, Force-directed network diagrams of the 1,898 DO mouse phenotypes used in downstream analysis, with nodes corresponding to individual traits. Networks are colored by study (E) and trait type (F). *Cal. Int.* - Calico internal phenotypes. *Sven -* Svenson high fat diet study. *Pazdro* - Heart study. *Ches* - Chesler striatum study. *Meta -* meta analysis of lifespan. *Attie* - Pancreas and insulin secretion study. *BW* - bodyweight. *DFI & BCS -* Digital frailty index and body condition score. G, Plot of mean *h^2^* ratios among clusters of traits at different levels of hierarchical clustering (k). H, The location and LOD scores of the 884 QTL detected in meta-analyses of 383 clusters of genetically correlated traits (meta-traits). I, The number of QTL detected at LOD ≥ 6 in each of the 383 meta-traits plotted against the *h^2^* of each meta-trait. Points are colored by the percent of heritable trait variation explained by the detected QTL.

Haseman-Elston regression was next used to broadly characterize the degree to which traits spanning a variety of independent studies and physiological domains share genetic architecture in DO mice. To this end, publicly available data were amassed from 11 studies (sources listed in S1-S2 Tables and Data D1 available at https://doi.org/10.5061/dryad.sj3tx96gd) [13], some of which accompany published manuscripts [5,13,18–23]. To enable estimation of pairwise kinship across studies using different genotype arrays for sequencing, genotypes from mice were interpolated across a common set of 69,005 pseudomarkers (**Methods**, S1 Data) prior to analysis. Phenotypes from each study were also corrected for age, sex, diet and/or drug treatment (when applicable), and DO generation wave prior to z-score normalization. In total, our analysis drew from 7,233 mice and 15,339 phenotypes, including 12,075 diet-specific phenotypes and 9 aggregated phenotypes consisting of phenotypically clustered frailty measurements (**Methods**). The heritability of each trait was estimated and traits with *h*^2^ < 10% excluded. We also excluded phenotypes for which *h*^2^ exceeded 1 by greater than the standard error associated with the *h*^2^ estimate (**Methods**). After filtering, 7,233 traits remained, including 5,335 diet-specific phenotypes. The mean and median *h*^2^ of the remaining set of high-confidence traits were 45.93% and 34.8%, respectively (Fig 2A and S3 Table). The mean and median |*r*_*g*_| were 0.708 and 0.489, respectively Fig 2A). Of the 26,154,528 trait pairs examined, 276,0833 pairs (10.56%) of traits exhibited individually significant non-zero genetic correlations (p < 0.05), with 13,125 pairs (0.05%) remaining significant after multiple hypothesis correction (p-value ≤ 1.912x10^-9^) (S4 Table and Table D1 available at https://doi.org/10.5061/dryad.sj3tx96gd) [13].

1,898 of the 7,233 phenotypes were measured in studies that did not include dietary interventions or were corrected for diet prior to analysis. These traits were hierarchically clustered based on their patterns of *r*_*g*_ estimates across the entire dataset of 7,233 traits by taking the Pearson correlation coefficient of *r*_*g*_ vectors (‘Pearson genetic correlation’ or ‘*r*_*pg*_*’*, S5 Table) for each trait pair (**Methods**). *r*_*pg*_ values were significantly correlated with *r*_*g*_ estimates across pairs of traits (ρ = 0.58, p < 2.2x10^-16^; Spearman r = 0.67, p = p < 2.2x10^-16^) but were notably less biased towards extreme values (>1 or <-1) (Fig 2A-2B). Traits were then hierarchically clustered by the absolute value of *r_pg_*, since the strength of genetic correlations, rather than the sign, is indicative of shared genetic architecture (Fig 2C). Phenotypes with higher *r_pg_* tended to come from the same study (mean intra-study *r*_*pg*_ = 0.2063, 95% CI = 0.2060 – 0.2065; mean inter-study *r*_*pg*_ = 0.1166048, 95% CI = 0.1165 – 0.1167), an unsurprising result given that the studies integrated into our dataset tended to focus on singular organ systems or domains of physiology (Figs 2D-2E and S4). Traits reflecting similar aspects of physiology or biological processes tended to have higher *r*_*pg*_ with one another, although similar traits measured across studies were not necessarily genetically correlated (“body weight” from the DRiDO study and “body weight” from a high-fat diet study, for example) (Fig 2F).

### Mega-analysis of genetically correlated trait clusters identifies hundreds of QTL

We sought to perform meta-analyses on clusters of genetically correlated traits to identify loci influencing particular aspects of physiology. Maximizing power in such analyses requires defining trait clusters that include data from several genetically correlated phenotypes without under-clustering and aggregating data from genetically unrelated phenotypes. To do this, trait clusters were defined such that the *h*^*2*^ of aggregated meta-phenotypes (*h*^*2*^_*meta*_) was maximized relative to unclustered traits using a “*h*^*2*^ ratio” for each trait cluster (**Methods** and S5 Fig). We computed the mean heritability ratio of clusters from *k* = 1,897 (minimal clustering) to *k* = 2 (Fig 2G). The heritability ratio consistently increased as traits were clustered until reaching a global maximum at *k* = 383 (4.96 traits per cluster), suggesting that continuous clustering of traits until this point continuously increased *h^2^* of the resulting meta-traits relative to previous levels of clustering. After clustering, *r*_*g*_ and *r*_*pg*_ were estimated combinatorially among the 383 phenome-wide meta-traits (C_1_ - C_383_; S6 Fig) to facilitate downstream analysis (S6-S11 Tables).

Genome-wide QTL scans were performed on meta-traits C_1_ - C_383_ (Fig 2H and Data D3 at https://doi.org/10.5061/dryad.sj3tx96gd) [13]. At permutation-based, trait-specific, genome-wide significance thresholds (⍺ = 0.05, 1000 permutations; see Methods), 164 total QTL were discovered (S12 Table); while 884 QTL were identified at a global threshold of LOD > 6 (S7 Fig and S13-S14 Tables, see Methods). Given meta-trait-specific FDRs and the number of QTL detected in each genome-wide scan, we expect 125 (14.1%) of the 884 QTL to be false discoveries (see Methods). Loci discovered using ⍺ = 0.05 permutation-based thresholds explained an average of 3.5% of phenotypic variation in their respective meta-analyses, while inclusion of loci discovered at LOD > 6 threshold increased this percentage to 19.46% (Fig 2I). The mean *h*^2^ of the 383 meta-traits was 36.5%, and the mean percentage of *h*^2^ explained by loci detected at genome-wide significance thresholds was 8.13%. Inclusion of loci at LOD > 6 increased this percentage to 40.36%.

### The genetics of lifespan are modestly correlated across independent DO studies

The atlas of *r*_*g*_ data included lifespan measurements from three independently conducted studies of lifespan in DO mice (“Harrison”, “Shock”, and “DRiDO”), as well as a meta-trait combining those three studies (“meta_lifespan3”) [13,18]. Collectively, these studies include lifespan measurements for 2,308 mice (232 males and 2076 females) across six diets (ad libitum, or “AL”; 20%, 30%, and 40% caloric restriction, or ‘20CR’, ‘30CR’, and ‘40CR’; one and two day intermittent fasting, or ‘1D’ and ‘2D’) and one drug treatment (rapamycin, or ‘rapa’) (S4 Table at https://doi.org/10.5061/dryad.sj3tx96gd) [13]. Haseman-Elston-based estimates of *h*^*2*^ were 11.1% (SE = 8.3%), 12.6**%** (SE = 3.6%), and 23.7% (SE = 6.1%) for the Shock, Harrison, and DRiDO studies, respectively. These were similar to previously-reported REML-based *h*^*2*^ estimates of 15.0% (SE = 15%), 18.5% (SE = 6.5%), and 24.6% (SE = 7.7%) from the three studies, respectively [13].

**Fig 3:**
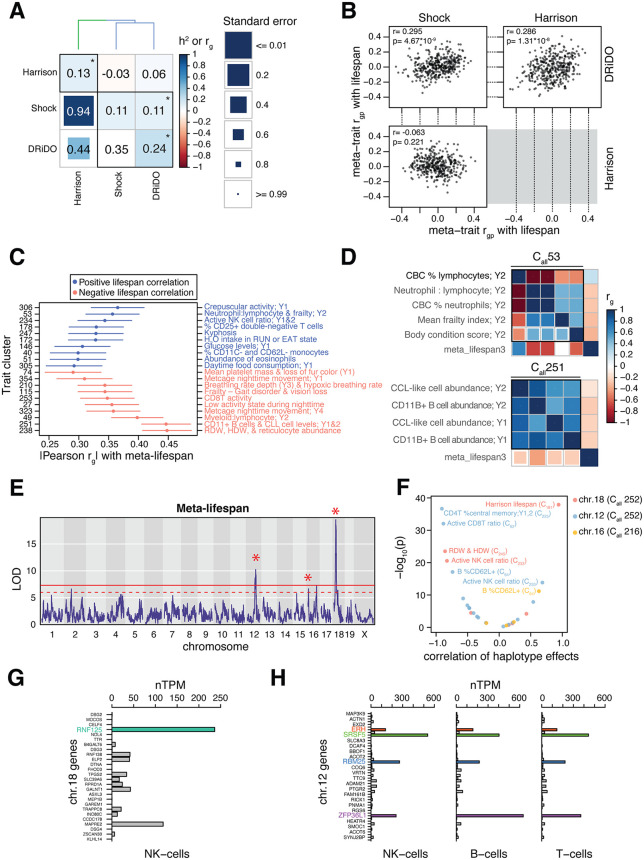
Genetic correlations involving measurements of lifespan in DO mice. A, Heatmap of genetic correlations and heritability estimates among three independent measurements of lifespan in DO mice. The diagonal of the heatmap reports the *h^2^* of each lifespan trait. The lower triangle of the heatmap shows *r_g_* among the lifespan traits and the upper triangle shows the *r_pg_* among lifespan traits. Asterisks denote statistically significant *h^2^*, *r_g_*, or *r_pg_* estimates. The dendrogram shows the results of hierarchical clustering of the lifespan traits based on euclidean distance derived from the *r_pg_* values among each pair of traits. Clusters of traits that result in a maximal mean *h^2^* ratio when combined are represented by colors in the dendrogram as well as black boxes in the heatmap. B, Scatterplots of *r_pg_* estimates between lifespan traits and the 380 other non-lifespan meta-traits. C, The strongest positive and negative *r_pg_* estimates between meta-traits and combined lifespan data. Error bars correspond to the standard error of each *r_pg_* estimate. Meta-trait number (cluster number) is shown on the left hand side of the plot and a brief summary of the phenotypes included in each meta-trait is shown on the right. D, Examples of trait clusters that, when aggregated into meta-traits, have high *r_pg_* estimates with combined lifespan data. C_all_ 53 (*top*) comprises midlife (year 2) traits reflecting relative abundances of neutrophils and lymphocytes in the blood and includes frailty index and body condition scores. C_all_ 251 (*bottom*) comprises CD11 + B and chronic lymphoid leukemia-like (CLL) cell abundance traits from early and midlife (years 1 and 2). E, Manhattan plot of an additive whole-genome scan of a mega-analysis of lifespan consisting of data from the DRiDO, Harrison, and Shock lifespan studies. The red line indicates a genome-wide significance threshold of ⍺ = 0.05 based on 1,000 permutations of the data (see Methods). The dashed red line indicates a FDR-based significance threshold of LOD >= 6 (corresponding to an FDR of ~9%). QTL mapped at genome-wide significance in the mega-analysis or one of the individual studies comprising the analysis are denoted by asterisks. F, Volcano plot depicting the correlation coefficient and -log_10_(p) of haplotype effects at lifespan loci with overlapping QTL from meta-analyses of other trait clusters. G, Expression of genes within the 2LOD support interval of the lifespan QTL on chr. 18 in human Natural Killer (NK) cells. H, Expression of genes within the 2LOD support interval of the lifespan QTL on chr. 12 in human NK, B, and T cells.

The mean of individual *h*^*2*^ estimates for lifespan from the DRiDO, Harrison, and Shock studies was 15.8% (SE = 4.0%), while the *h*^*2*^ of meta-lifespan, combining data from those three studies, was 11.7% (SE = 2.3%) (Fig 3A), suggesting some genetic factors contributing to lifespan to have had study-specific effects. Concordantly, the inter-study *r*_*g*_ estimates for lifespan were not statistically distinct from zero, and inter-study lifespan *r*_*pg*_ values were only significantly non-zero between the DRiDO and Shock studies (*r*_*pg*_ = 0.11; p = 1.34x10^-22^). The Harrison study had significant *r*_*pg*_ estimates with the other lifespan traits when correcting for the limited number of lifespan-to-lifespan correlations hypothesized (three hypotheses; Harrison-DRiDO: *r*_*pg*_ = 0.06, p = 9.69x10^-7^; Harrison-Shock: *r*_*pg*_ = -0.03, p = 4.5x10^-3^). As might be expected given their relatively low *r*_*pg*_ values, study-specific lifespan traits were not clustered into the same phenome-wide meta-traits. The *r*_*pg*_ estimates computed among the meta-traits were used to determine whether lifespan traits were genetically correlated with the same meta-traits (S6 and S8 Figs). The vector of *r*_*pg*_ values from the DRiDO study was significantly correlated with the *r*_*pg*_ values from both the Shock and Harrison studies, while the *r*_*pg*_ values from the Shock and Harrison studies were not significantly correlated (Fig 3B). The most highly correlated meta-traits were largely distinct between the three lifespan studies (S8 Fig).

Together, these results were consistent with a limited – but nonzero – overlap in the genetic basis of independently-collected lifespan measurements. Inclusion of varied interventional arms across these studies, including dietary modifications, may have contributed to their varied genetics. Genetic correlation estimates *r*_*pg*_ for lifespan assessed across various dietary intervention and pharmacological treatment groups were consistently positive (mean |*r*_*pg*_| = 0.09, SE = 0.012), with 17 of 28 pairwise comparisons (60.1%) demonstrating statistical significance, indicating a modest shared genetic architecture influencing lifespan under different intervention conditions (S9 Fig).

### Common genetic drivers influence lifespan and immune cell traits

Despite genetic differences in lifespan among studies, a previous meta-analysis of lifespan in DO mice detected six longevity-associated QTL, including a locus on chromosome 18 that reproducibly detected in two independent studies and additional loci mapped at genome-wide significance in individual studies on chromosomes 12 and 16 (Figs 3C and S10 [13]). Motivated by these findings, we considered genetic correlations among the meta-traits and aggregated lifespan data. The meta-traits sharing the highest |*r*_*pg*_| with meta-lifespan included frailty measurements [24], various metabolic cage readouts [19], and many immune cell phenotypes (Fig 3D and 3E) [18]. Mega-analysis of C_1_-C_383_ identified 19, 6, and 9 QTL with 2LOD support intervals that overlapped with the chromosome 12, 16.1 and 18 loci, respectively.

To determine if the lifespan QTL and those detected in other meta-traits were driven by the same DO founder alleles, we first performed variant association mapping at each lifespan QTL to determine a set of markers most likely to explain the effect of each locus, defined as the 1% of markers with the most significant effect on lifespan within the 2LOD support interval of each QTL (**Data D4** at https://doi.org/10.5061/dryad.sj3tx96gd) [13]. For each marker set, we calculated the Pearson correlation coefficient of the best linear unbiased predictors (BLUPs) of each DO founder allele on lifespan and on any overlapping QTL from C_1_ - C_383_. For all loci, the most significant correlations between allelic effects on lifespan and other traits (p ≤ 10^-10^) occurred between lifespan and meta-traits related to ratios of activated to naive B, T, and NK cells (Fig 3F). Notably, both lifespan-associated loci were detected in cluster C_all_233 (ratio of active to inactive NK cells in late life), and the same alleles appeared to drive effects on both sets of phenotypes. On chromosome 18, the CAST allele is associated with both decreased lifespan and an increased ratio of active NK cells; on chromosome 12, the CAST allele is associated with increased lifespan and decreased ratios of active immune cells, including CD4 + T cells, CD8 + T cells, CD62L + B cells, and NK cells (Fig 3F).

The 2LOD support intervals of the lifespan QTL on chromosome 12 and 18 encompass 48 and 26 genes, respectively (**Data D4** at https://doi.org/10.5061/dryad.sj3tx96gd) [13]. We utilized single-cell RNA-seq data from the Human Protein Atlas [25,26] to identify candidate genes within these intervals that are expressed in the relevant cell types for each lifespan QTL. At chromosome 18, ubiquitin ligase *Rnf125* is the most highly expressed gene in human natural killer cells (NK-cells) and has been shown to be a marker of NK-cells in both humans and mice (Fig 3G) [27,28]. At the chromosome 12 locus, a set of four genes appear more highly expressed across NK-cells, B-cells, and T-cells: *Erh*, *Srsf5*, *Rbm25*, and *Zfp36l1* (Fig 3H). The chromosome 16 locus was resolved to a single protein-coding gene, the RNA binding protein *Rbfox1*, which is expressed at low levels in B-cells.

### Mid-life frailty indexes shared limited genetic correlation with lifespan

We sought to quantify the extent to which frailty indexes reflect the underlying genetic basis of lifespan by genotyping animals from a prior study on frailty performed using DO mice [24]. Heritability and genetic correlations were calculated for both a video-based digital frailty index (DFI) and a manual frailty index (MFI) [29,30], as well as for the individual components of each frailty index. We also implemented a version of the traditional open field assay [31] amenable to the varied coat colors of DO mice (see Methods), and collected a large corpus of DO data using this assay in order to include the phenotypes measured in our genetic analysis. Due to precedence for inclusion of open field in frailty assessment [30], these traits were analyzed in conjunction with MFI and DFI. Across animals from which both open field and FI data were collected (N = 125; see Methods), each FI score and chronological age had negative phenotypic correlations with the performance-based open field phenotypes (total distance travelled, gait speed, angular rotation, distance per rotation) and positive correlations with exploration of the middle of the box (S11A Fig).

**Fig 4:**
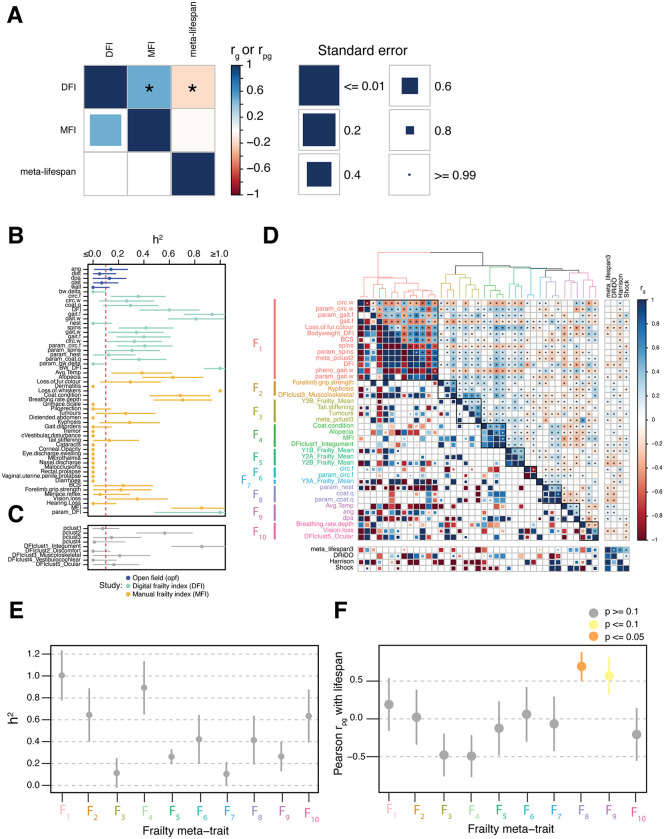
Genetic correlations among frailty and lifespan phenotypes. A, Pairwise (*r*_*g*_, bottom triangle) and Pearson (*r*_*pg*_*,* upper triangle), genetic correlations among Manual Frailty Index (MFI), Digital Frailty Index (DFI), and lifespan. Statistically significant genetic correlations are denoted with an asterisk. Box color represents the magnitude of the genetic correlation and box size is inversely correlated with the size of the standard error. B-C, Heritability and standard error of individual (B) and aggregate (C) frailty phenotypes. The dashed red line at *h^2^* = 0.1 is the threshold used to determine if phenotypes were included in downstream analysis. D, Hierarchically clustered heatmap showing *r_g_* (*lower triangle*) and *r_pg_* (*upper triangle*) among frailty phenotypes. The accompanying dendrogram shows euclidean distances among traits based on their r_pg_ estimates. Clusters of frailty traits (F_1_ - F_10_) that result in a maximal mean *h^2^* ratio when combined are represented by colors in the dendrogram as well as black boxes in the heatmap. *r_g_* and *r_pg_* among frailty and unclustered lifespan phenotypes are shown in the adjacent panels of the heatmap. E, Heritability and standard error of the meta-traits comprising clusters of genetically correlated frailty phenotypes. F, *r_pg_* and standard error among frailty meta-traits and a meta-analysis of lifespan spanning three studies.

Haseman-Elston-based *h*^*2*^ estimation yielded 0.854 (SE = 0.238) for MFI and 0.600 (SE = 0.237) for DFI. In agreement with the phenotypic correlations for these phenotypes measured in DO mice by [24]; r = 0.22; p value = 1.8 × 10^−7^), these two versions of FI shared significantly positive genetic correlations (*r_g_* = 0.484, p value = 0.041; *r_pg_* = 0.492, p value < 9.88x10^-324^). The most highly-powered lifespan phenotype (meta-lifespan) was not genetically correlated with MFI (*r_g_* = -0.001, p value = 0.998; *r_pg_* = -0.023, p value = 0.048); but shared a significant negative Pearson genetic correlation with DFI (*r*_*g*_ = 0.173, p value = 0.582; *r_pg_* = -0.184, p value = 5.20x10^-56^) (Fig 4A).

While the insignificance of most *r*_*g*_ and *r*_*pg*_ values between the frailty indexes and lifespan suggested largely non-overlapping genetics, the possibility remained that individual FI components might possess greater but obfuscated correlations, warranting individual analyses. Across the 55 scored components of MFI, DFI, and open field, 37 (67%) had significantly non-zero *h*^*2*^, with 32 (58.2%) exceeding the 10% *h*^*2*^ threshold we imposed for genetic correlation analysis (Fig 4BA and S3 Table). One more trait (“loss of whiskers”) was excluded from subsequent analysis because its estimated *h*^2^ exceeded 100% by more than its standard error. The remaining 31 frailty components (mean, median *h^2^* = 32.3%, 25.6%) had been included in the already-described phenome-wide genetic correlation analysis. We additionally constructed nine aggregate phenotypes (Fig 4C) based on either *a)* a phenotypic correlation analysis (S11B Fig), or *b)* groupings defined by the authors of the MFI [30]. Of the resulting nine aggregate traits, five had *h*^*2*^ ≥ 10%.

Genetic correlations were then estimated among the 31 individual frailty phenotypes, the five heritable aggregate traits, and the rest of the mouse phenome (including lifespan). Individually, none of the individual or aggregate frailty traits had significant *r*_*g*_ with meta-lifespan; however, 18 of the 36 frailty traits (50.0%) had significant *r*_*pg*_ with meta-lifespan when accounting for phenome-wide multiple testing (p value ≤ 1.91x10^-9^) (Fig 4D). Among these, the mean |*r*_*g*_| with meta-lifespan was 0.364 and the mean |*r*_*pg*_| with meta-lifespan was 0.092, implying a low, but non-zero, overlap in the genetics of frailty traits and lifespan. Individual and aggregate frailty phenotypes (including MFI and DFI) were hierarchically clustered into 10 meta-traits (F_1_ - F_10_) based on their phenome-wide *r*_pg_ values such that the *h*^*2*^ of the resulting meta-traits was maximized S11C Fig and Fig 4E and S15 Table). We estimated each frailty meta-trait’s genetic correlation with meta-lifespan and used these estimates to compute *r*_pg_ between frailty meta-traits and lifespan (using only the correlation across lifespan and these 10 meta-traits). Of the 10 frailty meta-traits, only two appeared to share the most genetic overlap with lifespan: F_8_ (nesting and coat quality; *r*_*pg*_ = 0.69, p = 0.018) and F_9_ (temperature and two open field behavioral traits; *r*_*pg*_ = 0.57, p = 0.067) (Fig 4F).

### Meta-analysis of histology-derived aorta phenotypes reveals genetic drivers of elastin breakage

**Fig 5:**
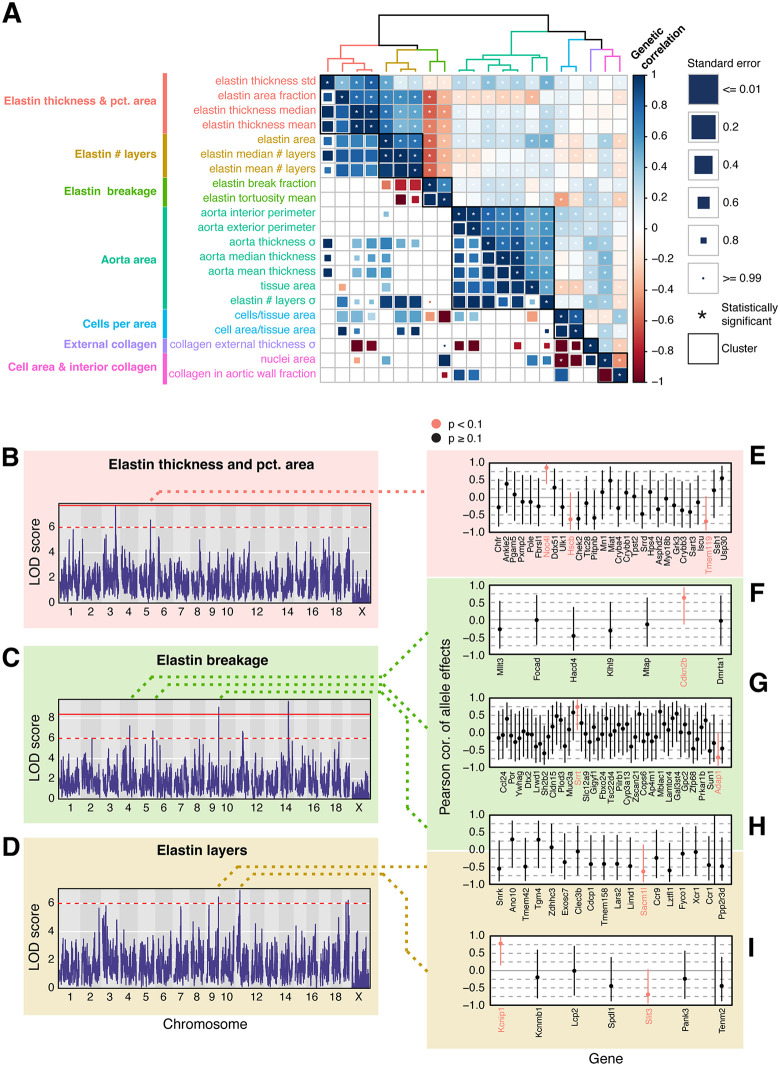
Genetic correlations among extracellular matrix traits in the aorta. A, Hierarchically clustered heatmap showing *r_g_* (*lower triangle*) and *r_pg_* (*upper triangle*) among aorta phenotypes. The accompanying dendrogram shows euclidean distances among traits based on their *r_pg_* estimates. Clusters of aorta phenotypes that result in a maximal mean *h^2^* ratio when combined are represented by colors in the dendrogram as well as black boxes in the heatmap. B, Manhattan plot of an additive whole-genome scan on aggregated aortic elastin thickness and pct. area phenotypes. The red line indicates a genome-wide significance threshold of ⍺ = 0.05 based on 1,000 permutations of the data (see Methods). The dashed red line indicates a nominal significance threshold of LOD >= 6. C, Manhattan plot of an additive whole-genome scan on aggregated aortic elastin breakage phenotypes. D, Manhattan plot of an additive whole-genome scan on aggregated aortic elastin layers phenotypes. E–I, Correlations of haplotype effects between the peak markers at QTL influencing elastin phenotypes and *cis*-eQTL in an external DO cardiac tissue transcriptomic dataset. Candidate genes are highlighted with red text.

A major advantage of DO mice is the ability to perform genetic analyses on phenotypes that would be difficult or impossible to collect from humans, e.g., histological analysis of non-expendable tissues. We conducted a novel histological study of the aortic extracellular matrix using 116 DO mice. Image analyses were performed on aortic cross-sections stained with H&E, TC, or VVG, yielding 26 phenotypes, 21 of which had *h*^*2*^ ≥ 10% (see Methods; S12 Fig). Phenotypes were hierarchically clustered into seven meta-traits (A_1_ - A_7_, S16 Table) using their phenome-wide *r*_*pg*_ values, such that the *h*^*2*^ ratio was maximized across meta-traits (Figs 5A and S13). Broadly, these meta-traits comprised *1)* elastin laminae thickness & % aortic area consisting of elastin, *2)* the number of elastin layers in the aorta, *3)* elastin breakage and tortuosity, *4)* tissue area, *5)* nuclei per tissue area, *6)* external collagen, and *7)* nuclear area and fraction of collagen inside the aorta. A meta analysis of each cluster was performed (S2 Data), identifying three QTL at permutation-based significance of ⍺ = 0.05 and an additional 14 loci at a significance threshold of LOD>= 6 [13], for a total of 17 QTL (2.43 QTL/meta-trait) (Fig 5B-5D). Given the FDR of each aorta meta-trait at LOD 6, we expect 14 (82.4%) of the 17 loci to be true positives (S15 Fig and S17 Table).

Two, six, and four QTL were detected associated with A_1_ (elastin thickness and percentage of aortic area consisting of elastin area), A_2_ (layers of elastin in the aorta), and A_3_ (elastin breakage), with a locus on chromosome 9 contributing to both A_2_ and A_3_ (Fig 5 and S18 Table). We estimated effect sizes (best linear unbiased predictors, or BLUPs) and performed association mapping on the 2LOD support interval about each QTL (S16-S17 Figs). Collectively, the QTL explain 28.59%, 51.87%, and 106.91% of variation in elastin area, breakage, and layers, respectively; however, due to the low sample sizes associated with these phenotypes (mean n = 112.57), the variance attributable to the QTL are likely inflated due to the Beavis effect. Most confidence intervals were large, spanning an average of 33 protein-coding genes (S3 Data), although two QTL were mapped at single gene resolution: *Olfm4* influencing elastin breakage on chromosome 14 and *Sorcs1* influencing the number of layers of elastin on chromosome 19 (see Discussion).

To narrow down the list of candidate genes influencing elastin in the aorta, we relied on data from an external whole-heart RNA-seq experiment [32]. For each QTL, we searched for expression QTL (eQTL) associated with genes within 2Mb of the peak marker in our meta analysis. We then computed the Pearson correlation coefficient of allele effects between the peak markers in the RNA-seq and meta-analysis datasets (Figs 5E-5I and S18). For five of the QTL, one or more candidate genes were identified with expression patterns driven by the same alleles contributing to elastin phenotypes in the aorta. Notably, we identified *Cdkn2a* as the most likely candidate within the elastin breakage QTL on chromosome 4 (Fig 5F; see Discussion).

### Meta-analysis of functional and histology-derived lung phenotypes reveals genetic contribution to pulmonary inflammation and/or tumorigenesis

In order to broadly characterize the genetic landscape of lung function in DO mice, we measured 79 functional lung phenotypes, including image-derived phenotypes from µCT scans as well as H&E and TC stains of lung tissue sections, in another distinct cohort of 240 mice, including 124 C57BL/6J and 116 DO mice (S19 and 6 Figs). The patterns of phenotypic correlations were highly similar when measured within the C57BL/6J versus DO populations (Pearson correlation across phenotype correlations = 0.737; p value < 2.2x10^-16^; S20A-B Fig), suggesting physiological generalizability between these inbred and outbred mice.

**Fig 6:**
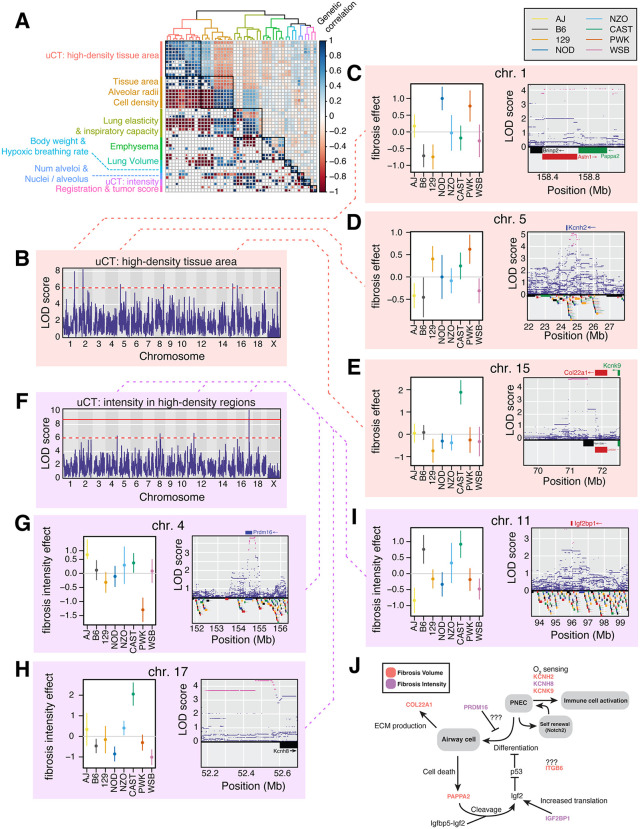
Genetic correlations among lung structural and functional phenotypes. A, Hierarchically clustered heatmap showing *r_g_* (*lower triangle*) and *r_pg_* (*upper triangle*) among lung phenotypes. The accompanying dendrogram shows euclidean distances among traits based on their *r*_*pg*_ estimates. Clusters of lung phenotypes that result in a maximal mean *h*^*2*^ ratio when combined are represented by colors in the dendrogram as well as black boxes in the heatmap. B, Manhattan plot of an additive whole-genome scan on aggregated fibrosis volume phenotypes. The red line indicates a genome-wide significance threshold of ⍺ = 0.05 based on 1,000 permutations of the data (see Methods). The dashed red line indicates a nominal significance threshold of LOD >= 6. C–E, Allelic effects (BLUPs) and corresponding standard errors of the QTL influencing fibrosis volume in the lung (*left*), and variant association mapping of the 2 LOD support intervals around each QTL (*right*). Genes within the confidence intervals are shown in the bottom panel, and candidate genes are shown above variant association results. F, Manhattan plot of an additive whole-genome scan on aggregated fibrosis intensity phenotypes. G–I, Allelic effects (BLUPs) and corresponding standard errors of the QTL influencing fibrosis intensity in the lung (*left*), and variant association mapping of the 2 LOD support intervals around each QTL (*right*). J, Model depicting the cellular functions of pulmonary neuroendocrine cells (PNECs) and showcasing the roles of candidate genes may play in contributing to fibrotic µCT readouts in the lung. Candidate genes are colored by the whole-genome scan in which they were detected.

Of the 79 lung phenotypes, *h^2^* estimates met or exceeded 10% for 49 (62%; S20C Fig). Those 49 phenotypes were therefore included in downstream genetic analyses. As above, phenotypes were hierarchically clustered by their *r*_*pg*_ values across the 1,898 phenotypes and grouped to maximize the *h^2^* ratio of each meta-trait (S21 Fig), resulting in 9 clusters of traits (L_1_ - L_9_; Fig 6A) enumerated in S19 Table. Meta analyses were performed on each cluster, identifying a single QTL at ⍺ = 0.05 permutation-based thresholds and an additional 31 loci at LOD >= 6, for a total of 32 QTL (3.56 QTL/meta analysis, Figs 6B, 6F and S22 and S20 Table). Given the FDR of each lung meta-trait at LOD 6, we expect 25 (78.1%) of the 32 loci to be true positives (S23 Fig and S21 Table).

Two clusters of lung phenotypes – L1 and L8, corresponding to the % of high-density tissue and voxel intensity of high-density tissue, respectively – were µCT-derived lung tissue density measurements that may reflect several pathological conditions of the lung, including pulmonary fibrosis, inflammation, tumor presence, or edema [33]. To better understand the nature of the µCT-derived tissue density phenotypes, we examined the phenotypic relationships between µCT measurements and fibrosis measurements derived from trichrome (TC) staining of lung tissue sections, which are expressed in the percent area of the lung that stains for collagen (S24A-G Fig). In C57BL/6J mice, the TC-derived collagen fraction increases from day 120 to day 600 (S24A Fig), while the % area of high-density tissue and voxel intensity in high-density areas decreases between 120 and 150 days before increasing with age (S24B-C Fig). Due to the resolution of the measurements, it is unclear whether the same trend occurs for collagen ratio in the lungs. In DO mice, the fraction of collagen and voxel intensity of high-density areas increased with age but not the fraction of high-density tissue (S24D-F Fig). The area of high-density tissue was not significantly correlated with collagen fraction (r = -0.131, p = 0.299;(S24G Fig) but was significantly correlated with mean distance between nuclei (r = -0.424, p = 3.85x10^-6^; S24H Fig) and presence of ectopic cell clusters (r = 0.414, p = 7.05x10^-6^; S24I Fig), both of which are hypothesized to reflect inflammation and/or tumorigenesis in the lung tissue.

Meta analysis of uCT-derived tissue density phenotypes resulted in the detection of 6 QTL influencing the % high-density tissue and 4 QTL influencing the voxel intensity of high-density tissue (Fig 6B and 6F and S20 Table and S4 Data). These QTL explain 94.2% and 62.7% of phenotypic variation in the traits, respectively; however, due to low sample sizes these estimates of QTL effects are likely inflated due to the Beavis effect [34]. Confidence intervals at the % high-density tissue and voxel intensity QTL spanned 19 and 49 genes on average, respectively (S5 Data). However, variant association mapping resolved the locus on chromosome to the *cis*-regulatory region of a single gene, *Kcnh8*, which encodes a voltage-gated potassium channel expressed specifically in pulmonary neuroendocrine cells (PNECs) within the lung (Fig 6I) [35]. Two additional potassium channels with PNEC-specific lung expression patterns, *Kcnh2* and *Kcnk9*, were identified within the 2LOD support intervals of QTL on chromosomes 5 and 15, respectively (Fig 6D-6E), with variant association mapping refining the set of candidate SNPs at chromosome 5 to those in the 5’ upstream region of *Kcnh2* (Fig 6D). *Pappa2*, a protease that activates PNEC mitogen *IGF2* via cleavage of the *IGFBP5-IGF2* complex [36], was one of three genes underlying the % high-density tissue QTL on chromosome 1 (Fig 6C). Variant association mapping localized the effect at the chromosome 4 locus to candidate SNPs in the upstream region of *Pdrm16* (Fig 6G) [37] and *Igf2bp1*, which increases levels of bioavailable *IGF2* through stabilizing interactions with its mRNA [38], on chromosome 11 (Fig 6H).

## Discussion

Our novel implementation of Haseman-Elston regression for the estimation of *r*_*g*_ in DO mice provides a tool by which phenotypes can be jointly analyzed across studies, timepoints, environments, and intervention groups. We first used *r*_*g*_ estimates to assess the extent to which age and dietary interventions impact the genetic architecture of complex traits including energy expenditure, food and water consumption, hematology, adiposity, frailty, immune cell, and body weight. Intra-trait *r*_*g*_ suggested that gene-by-diet (*GxE*) interactions played a greater role in specifying phenotype than gene-by-age (*GxAge)* interactions across nearly all types of traits, with three exceptions – acoustic startle, frailty and grip – all of which relate to the deterioration of the body with age. Notably, the mean intra-trait *r*_*g*_ among body weight phenotypes was negative implying a shared genetic architecture in which some variants have opposite effects on body weight depending on an animal’s diet. While intra-trait *r*_*g*_ was higher among traits at early timepoints, these estimates decreased on average when comparing measurements from earlier timepoints (years 1–3) to those taken late in life (year 4). This finding suggests that the effects of variants may change more rapidly later in life, or it may reflect a survival bias present in the DRiDO study: most of the animals that survived to the fourth time point were under severe (40%) caloric restriction, which was previously shown to have dramatic effects on longevity and other health-related outcomes.

### Overcoming the limited power of DO mouse genetic studies through mega-analysis

Extending Haseman-Elston based *r*_*g*_ estimates to over 7,000 DO mouse phenotypes facilitated a cross-study hierarchical clustering of traits based on genetic similarity. While individual *r*_*g*_ estimates were noisy, hierarchical clustering based on patterns of genetic correlations (*r*_*pg*_) across the entire dataset produced 383 clusters of traits that, when aggregated for mega-analysis, increased statistical power for QTL discovery. To date, no larger mega-analysis has been conducted in DO mice, and our approach resulted in the detection of hundreds QTL influencing lifespan, immune composition, aortic tissue composition, and lung function, and the QTL detected in meta-analyses explained a large fraction of variance (over 40%) in their respective meta-traits. While more detailed genetic analyses were performed on several phenotypes of interest in this study (*see below*), many sets of correlated phenotypes remain to be analyzed, and we hope this dataset will be valuable to those interested in genetic analysis in DO mice.

The granularity of clustering used in this analysis was specified to maximize mean *h*^*2*^ across all mega-analyses. However, we note that power increases in mega-analyses can arise from either i) combining multiple measurements of correlated traits from the same set of animals, or ii) combining correlated traits measured in different sets of animals. The former can increase the signal-to-noise ratio (*h*^*2*^) of genetic analysis if the error terms associated with combined traits from the same animals are orthogonal and cancel out when the traits are aggregated. The latter increases the sample size through the inclusion of additional animals, which will not increase *h*^*2*^ but should increase the precision of the estimate. In practice, mega-analyses performed in this paper allow for both approaches towards trait clustering but as clustering was optimized for maximum *h*^*2*^, trait clusters in this analysis may be biased towards the combination of measurements from within cohorts. Future implementations may incorporate the standard error of the *h*^*2*^ estimate as a criteria for clustering. Furthermore, a global solution to maximize *h*^*2*^ may not produce clustering ideal for any one particular set of phenotypes; alternative clustering solutions for individual phenotypes may be explored in the future.

Despite leveraging genetic correlations to boost power, the 884 QTL identified in our mega-analyses account for only 40.36% of the heritable variation for the average meta-trait. A previous power analysis in DO mice reports that cohorts between 800–1000 individuals provide a ~ 90% probability to detect loci explaining 3–5% of phenotypic variance in a trait, with a rapid decline in the probability of detection for smaller effects [2]. Together, these results suggest polygenic architectures composed of small-effect loci (≤1–3% phenotypic variance explained) underlie many of the meta-traits reported in this manuscript. Beyond genetic architecture, statistical power is further limited by the modest sample sizes of typical DO studies as well as the low heritabilities of many traits. As additional DO cohorts are phenotyped, the power to detect QTL via mega-analysis should improve due to increased sample size and mapping resolution.

### Identification of common genetic drivers between mouse lifespan and correlated phenotypes

Estimates of *r*_*pg*_ between lifespan measurements from three independent studies (Harrison, Shock, and DRiDO) revealed significant but modest correlations, suggesting potential differences in the genetic architecture of lifespan across study. Each lifespan study was conducted independently in different facilities/rooms using different chow, feeding schedules, temperatures, housing arrangements, and personnel, all of which could potentially redistribute mortality risk and influence the effects of genetic variants on lifespan. The inclusion of different experimental intervention groups may have also influenced the genetic factors responsible for longevity. For instance, the Harrison study examines dietary and pharmacological lifespan interventions in female mice, while the Shock study focuses on lifespan in males in females without intervention [13]. Lifespan phenotypes from these studies were not genetically correlated (Fig 3A) and were correlated with different sets of meta-traits (Fig 3B). Meanwhile, lifespan data from the DRiDO study, which includes large numbers of animals with and without interventions [18], had patterns of *r*_*pg*_ estimates with meta-traits that were significantly correlated with both the Harrison and Shock studies (Fig 3B), implying that the genetics of lifespan in the DRiDO study likely encapsulates genetic effects relevant to both of the other two studies.

Despite low *r*_*pg*_ among independent lifespan measurements, some QTL influencing lifespan are detected in multiple studies [13] and are reproduced in a meta-analysis of longevity in DOs. A lifespan locus on chromosome 18, for example, was previously reported to affect variation in red blood cell volume [18]; here, the locus also shows correlated effects on the ratio of CD11b^-^/CD11c^-^ to CD11b^+^/CD11c^+^ natural killer (NK) cells that may be driven by the ubiquitin ligase *RNF125*. A second lifespan locus on chromosome 12 has correlated effects on several clusters of genetically correlated immune cell abundance and activity traits and implicates *ERH*, *SRSF5*, *RBM25*, or *ZFP36L1* as genes potentially influencing lifespan in DO mice. Collectively, our results suggest that variation in genes related to the immune system are contributing the most to variation in lifespan across DO mouse studies.

Frailty indexes (FIs), which each measure multiple clinically defined signs of deterioration in mice [24,30], generally produce scores that correlate with age and are used as a measurement of overall health in animals. However, the extent to which these indexes reflect the underlying genetics of lifespan are unknown. While many individual frailty measurements were not significantly genetically correlated with lifespan, aggregate index scores (MFI, DFI, and clusters of phenotypically correlated frailty metrics) did often have significant, albeit weak, *r*_*pg*_ with lifespan. One interpretation is that these individual measurements may capture some fraction of the genetic variation contributing to lifespan in addition to other components unrelated to longevity, while the signal from the variants associated with aging or longevity are retained in the aggregate scores. Clustering analysis of frailty traits revealed two clusters, F8 and F9, that were most genetically correlated with lifespan. These clusters represented traits such as nesting behavior, coat quality, temperature, and movement-related traits from the open field behavioral assay, indicating that a subset of frailty traits could potentially serve as surrogate markers for predicting longevity, albeit with limitations.

### Genetic contributors to the structural health of the mouse aorta

Diseases of the aorta are a significant contributor to human mortality, driven in part by the loss of the elastic properties of the tissue due to fragmentation, thinning, and improper cross-linking of elastin fibres. Mutations in the elastin gene *Eln* and other aortic extracellular matrix (ECM) proteins (e.g., fibrillins and collagens) are known to negatively affect the structure and function of the aorta, leading to adverse cardiac events. However, outside of proteins that comprise the aortic ECM, little is known about the genetic factors that influence the microstructure of aortic elastin filaments. Meta-analysis of elastin phenotypes revealed 12 novel QTL influencing elastin structure in the aorta; notably, none of these loci overlapped *Eln*, *Fbln-2*, *Fbln-5*, *Mmp-2*, or *Mmp-9*, genes implicated in aortic dissection and other cardiac events [39–42]. However, several notable candidate genes were identified, including *Cdkn2a*, a cyclin-dependent kinase that had previously been mapped in a genome-wide association study of human lifespan [43]and was previously associated with cardiovascular disease [44]. Another notable candidate was *Olfm4*, which plays an important role in innate immunity and inflammation. Knockdown of this gene reduced inflammatory response in lipopolysaccharide (LPS)-stimulated rat cardiac cells, resulting in enhanced proliferation and decreased apoptosis. *Olfm4*, an olfactomedin domain-containing glycoprotein regulated by NF-κB, is another noteworthy candidate that is involved in innate immunity and inflammation [45]and plays a protective role in the digestive tract [46]. Chen *et al* found that suppression of *Olfm4* has a protective effect in lipopolysaccharide (LPS)-stimulated cardiomyocytes [47], and the gene has emerged as a biomarker for the severity of certain infections [48]. Association of this gene with elastin breakage suggests a genetic link between inflammation and degradation of the ECM within the aorta.

### Genetic contributors to the functional health of the mouse lung

Micro-CT-based imaging of the lung in DO mice revealed numerous candidate genes influencing variation in tissue density of the lungs of adult mice. These measurements are unlikely to reflect pulmonary fibrosis, but may capture inflammation, a key characteristic of interstitial lung disease (ILD). Interestingly, three of the candidate genes (*Kcnh2, Kcnh8,* and *Kcnk9*) are voltage-gated potassium channels expressed robustly in pulmonary neuroendocrine cells (PNECs) and have been proposed as a mechanism by which PNECs function as O_2_ sensors within the airways [35]. In addition to this role, PNECs have been shown to regulate inflammation in the lung through the secretion of CGRP, suggesting a potential relationship between O_2_ sensing, immune cell recruitment, and fibrosis in the lung. PNECs may also function as stem cells in response to airway damage [49,50], which may be induced via IGF2-IGFR signaling,and are putative precursors to small cell lung cancer (SCLC) [51]. Notably, two additional candidates, *Pappa2* and *Igf2 bp1*, may influence PNEC stem cell activity by modulating levels of bioavailable IGF2; the former through cleavage of IGFBP5, which binds IGF2 and prevents signaling, and the later through stabilizing mRNA encoded by the *Igf2* locus. A summary of candidate genes, and how they relate to the functions of PNECs, is depicted in Fig 6J. In addition to candidates directly implicated in PNEC biology, *Prdm16* has been shown to play a role in suppressing both pulmonary fibrosis via antiproliferative effects [37,52]. Together, the candidates identified in our analysis suggest an important role of PNEC biology in ILD and/or tumorigenesis.

### Discovery opportunities deriving from future data-sharing across the DO mouse research community

DO mice are a powerful model system for studying the genetics of complex traits, with great potential to parallel and complement human genetic studies. The human and DO mouse genetic research communities have common commitments to the open sharing of data. In the case of DO mice, the lack of personal privacy concerns enables easy sharing of raw individual-level data. Although large, open-access databases of DO phenotypes and mapping results have already been established [5], the DO community currently lacks the breadth of tools and resources available for facilitating cross-study genetic analysis compared to human cohorts. The implementation of Haseman-Elston regression to estimate genetic correlations among independently-collected traits provides a new mechanism for collaborative analysis across independently-conducted DO studies that is akin to the utility provided to human studies by the LDSC tool [9]. Here, we have used it to unite the corpus of DO physiological and behavior studies into a common framework, enabling insights that would not have been possible from those studies in isolation.

We have sought to be comprehensive in our integrated analysis of available DO mouse data. However, the true value of these analyses was realized through focus and review of the patterns of clustering and QTL from particular domains of biology. Here, we focused on gleaning insight from our personal areas of interest: lifespan, frailty, and elastic tissues/organs (aorta and lung). We hope that the availability of these clusters will enable similar insights across domains where we have chosen not to focus our attention – including immune cell composition, hematological parameters, cardiovascular function, body weight, adiposity, feeding, circadian rhythm, pancreatic function, and bone strength. As new DO mouse studies are published, we hope to continue expanding this network into new domains using the Haseman-Elston method. In particular, recent expansions in available transcriptomic, metabolomic, proteomic and lipidomic measurements from DO mice (e.g., [22,53,54]) set the stage for the construction of comprehensive molecular correlation matrices.

## Methods

### Ethics statement

All procedures used in our studies were reviewed and approved by the Calico Animal Care and Use Committee.

#### Dataset curation.

Data used in these analyses were collected from a variety of sources, including internal Calico studies and publicly available studies of DO mice (S2 Table). In order to standardize genetic analysis, only datasets including age, sex, and DO generation wave information were included in our analysis. Curated datasets, including processed data used in downstream analyses, are included in a Dryad data repository [13].

#### Genotyping.

Genotypes for Calico internal studies were obtained using the mouse universal genotyping array (GigaMUGA; 143,259 markers) [55]. Genotyping was performed from tail tips by Neogen Genomics (Lincoln, NE, USA). Founder genotypes were reconstructed using the R/qtl2 software [56] and samples with call rates at or above 90% were retained for analysis.

Mice from all studies were genotyped using the MUGA, MegaMUGA, or GigaMUGA genotyping arrays (S2 Table) and genotypes were called using R/qtl2. To facilitate analysis across different arrays, genotype probabilities from each study were interpolated to a set of 69,005 pseudo-markers (S1 Data) and converted to founder haplotype probabilities via hidden Markov model in R/qtl2.

#### Adjustment and normalization of trait values.

To facilitate analysis of phenotype data across studies, all phenotypes were processed using a common workflow. First, for each phenotype, outliers –defined as trait values differing from the mean by an excess of five standard deviations– were excluded. To account for common covariates in DO mouse studies, we fit the following linear fixed-effects model:


Phenotype ~ Age + Sex + Generation + Diet\]

Where ‘Age’ corresponds to the age of the animals in days at the time of trait measurement, ‘Sex’ corresponds to the sex of the animals, ‘Generation’ corresponds to the DO mouse generation wave, and ‘Diet’ corresponds to any dietary interventions or drug treatments used in the studies (when applicable). We performed z-score normalization on the residuals of each model and the resulting phenotype scores were used for downstream analysis.

Phenotypes from the DRiDO study [18] included mice from different dietary groups. These phenotypes were included in the analysis as both aggregate traits spanning diet groups (which included a categorical ‘Diet’ term in the linear model) and as individual, diet-specific phenotypes. Diet-specific phenotypes were fit using fixed-effects models without a diet term and were processed and z-normalized separately from aggregated phenotypes.

Phenotypes from the Svenson HFD study [21] included mice that were fed a high fat diet for different numbers of days. These phenotypes were modeled using a continuous ‘Diet’ term that accounted for the number of days each mouse spent on the diet.

#### Estimation of narrow-sense heritability of traits via Haseman-Elston (HE) regression.

We estimated the narrow-sense heritability (*h^2^*) of traits in Diversity Outbred (DO) mice using Haseman-Elston (HE) regression [11], which involves regressing phenotypic similarity scores on kinship among pairs of individuals. While phenotypic similarity was originally defined as the squared difference in phenotype, later implementations would use the squared sib-pair sum [57], information from both the squared differences and squared sums [58], or vectorized outer product of phenotypes [12] as the dependent variable in the regression. Our implementation draws from Haseman & Elston’s “revisited” method [12], in which the vectorized outer product of z-normalized trait values among pairs of related individuals (S1A Fig) are regressed on their corresponding kinship estimates, as in S1B Fig. The model used in this regression takes the form of:


$${\text{M}}_{\text{i}}\text{\textbackslash{},}\times \;{\text{M}}_{\text{j}}=\;\alpha \;+\;\beta {\text{r}}_{i,j}\text{\textbackslash{},+ e}$$

Where *i* and *j* are a pair of related individuals, (M_i_ x M_j_) is the product of the z-score normalized trait values of trait *M* for *i* and *j*, and r_*i,j*_ is the standardized kinship estimate of *i* and *j*, defined as the average proportion of shared alleles among these individuals. These kinship estimates are computed using the calc_kinship() function in R/qtl2 [56]. This formulation differs slightly from previous implementations of Haseman-Elston in that a variance component is not estimated using a diagonal indicator [15], allowing the extension of the method to genetic correlations among traits measured in different cohorts of individuals. The slope, β_Y_, of the resulting best-fit line represents the covariance in trait values of Y as a function of relatedness, and can be converted to a phenotypic correlation of Y with itself as a function of additive genetic effects (in other words, h^2^) via the following equation:


$$\rho_{\text{M,M}}{\text{=h}}_{\text{\textbackslash{} \textbackslash{} M}}^{\text{2}}\text{\textbackslash{}hspace\{0.33em}=\;\beta {\}}_{\text{M,M}}{\text{/2}\sigma }_{\text{\textbackslash{}hspace\{0.33em}\;M}{\}}^{\text{2}}$$

Where h^2^_M_ is the narrow-sense heritability of trait Y and σ^2^_M_ is the variance in the measurement of Y [59]. Because *M* is normalized, σ^2^_M_ is equal to 1 and the equation is simplified to:


$$\rho_{\text{M,M}}{\text{=h}}_{\text{\textbackslash{} \textbackslash{} M}}^{\text{2}}\text{\textbackslash{}hspace\{0.33em}=\;\beta {\}}_{\text{M,M}}\text{/2}$$

The relationship between genotype (G), *h^2^*, phenotype (P), and correlation of trait values due to additive genetic variance (⍴_M,M_) for two individuals is depicted in a structural equation model in Fig 1C.

The standard error about the HE regression coefficient was computed using the following formula [16]:


$$SE(\beta_{M})\;=\;\frac{S_{\in }}{\sqrt{{\Sigma (x-\underset{―}{x})}^{\text{2}}}}=\;\frac{S_{\in }}{\sqrt{\Sigma x^{\text{2}}-\frac{(\Sigma {x)}^{\text{2}}}{n}}}$$

Where $SE(\beta_{M})$ is the standard error of the HE regression coefficient for trait *M* in the population, x refers to pairwise kinship estimates on which the vectorized outer product of *M* is regressed, and S_Ɛ_ is the residual standard error, defined as:


$$S_{\varepsilon }=\sqrt{\frac{\Sigma M^{\text{2}}}{n-2}\;}\;or\;\sqrt{\frac{\Sigma y^{\text{2}}}{n-2}\;}\;$$

Where *M* or *y* are the vectorized outer product of trait values for trait *M* that are regressed on pairwise kinship estimates and *n* is the number of pairwise observations. Then, as with the regression coefficient, the standard error of the HE regression coefficient was then divided by twice the variance of the z-score normalized trait *M* used in the regression:


$$SE(h_{M}^{\text{2}})\;=\frac{SE(\beta_{M})}{2\sigma_{M}^{\text{2}}}\;=\;\frac{SE(\beta_{M})}{\text{2}}\;$$

#### Assessing the performance of HE regression.

To assess the performance of our implementation of HE regression, we performed simulations in which HE regression was used to estimate the narrow-sense heritability of phenotypes with known genetic architecture and *h^2^* values using the ‘PhenotypeSimulator’ package in R [60]. Genotypes for simulated individuals were constructed from 1,000 unlinked diploid markers at seeded minor allele frequencies of 0.05, 0.1, 0.3, or 0.4. Kinship was computed from standardized genotype values and divided by 2 to reflect the probability of sampling the same allele twice from a highly heterozygous individual, as in DO mice. Simulated fixed genetic effects were additive and entirely independent of one another; in each simulated population, 10/1000 genetic markers were randomly selected as causal and effect sizes of these markers were drawn from a normal distribution. Individual phenotypes were computed without noise from the simulated genotypes and independent background noise was added to the set of phenotypes according to the *h^2^* of the trait being simulated. At each sample size and *h^2^*, 1000 different populations and sets of phenotypes were simulated, and HE regression estimates were computed (S1D Fig).

Haseman-Elston regression has been previously shown to produce *h*^*2*^ estimates similar to those from an orthogonal Bayesian model (**Re**stricted **M**aximum **L**ikelihood; REML) [14]. We tested the performance of our implementation of HE regression by repeating this comparison using a set of 36 physiological traits in DO mice [8]. HE regression was used to estimate *h*^*2*^ on the residual values for these traits after correcting for age, sex, generation wave, and diet using a linear fixed-effects model of the form:


Y ~ age + sex + genwave + diet + ε\]

where sex, generation wave, and diet were encoded as factors and age in days was encoded as a numeric variable. The resulting HE regression estimates were compared directly to the REML heritability estimates reported in the original study (Fig 1A).

#### Extension of Haseman-Elston regression to genetic correlations.

As in [17], HE regression can also be applied to the estimation of genetic correlations for pairs of traits. This involves regressing the z-score normalized vectorized outer product of trait values for two traits, *M* and *N*, among pairs of individuals on their pairwise kinship estimates (S1**A-B Fig**):


$${\text{M}}_{\text{i}}{\times \text{N}}_{\text{j}}\text{\textbackslash{}hspace\{0.33em}=\;\;\}\alpha \;+\;\beta {\text{r}}_{\text{i,j}}\text{\textbackslash{}hspace\{0.33em}+e\}$$

The slope of the resulting best-fit line, β_M,N_, represents the covariance in the vectorized outer product of traits *M* and *N* as a function of relatedness, and can be converted to a phenotypic covariance of traits *M* and *N* as a function of additive genetic effects using a slightly modified form of the prior equation:


$$\rho_{\text{M,N}}\text{\textbackslash{}hspace\{0.33em}=\;\beta {\}}_{\text{M,N}}{\text{/2}\sigma }_{\text{M}}\sigma_{\text{N}}$$

Which again simplifies due to the use of z-score normalized trait values in the regression:


$$\rho_{\text{M,N}}{\text{=}\beta }_{\text{M,N}}\text{/2}$$

While ⍴_M,M_ is equivalent to narrow-sense heritability for trait *M*, because trait *M* is perfectly genetically correlated with itself by definition, this relationship does not hold true for a pair of traits that are not genetically equivalent (S1C Fig). Instead, ⍴_M,N_ is a function of the heritabilities of *M* and *N* as well as the genetic correlation of the two traits:


$$\rho_{\text{M,N}}\text{\textbackslash{}hspace\{0.33em}=\;h{\}}_{\text{M}}{\text{h}}_{\text{N}}{\text{r}}_{\text{g M,N}}$$

Where *h_M_* and *h_N_* are the square roots of the heritabilities of traits *M* and *N*, respectively, and *r_g M,N_* is the genetic correlation of *M* and *N*. Thus, the genetic correlation for a pair of traits can be computed from three pieces of information: *h^2^* estimates for each individual trait and HE regression coefficient for the pair of traits.

The standard error about HE regression coefficients for traits *M* and *N*, as well as for the corresponding *h*^*2*^ estimates were computed as detailed above. Standard error estimates were propagated through the structural equation model to compute SE for the r_g_ estimate via the following equation [61]:


$$SE\left(r_{g\text{\textbackslash{}hspace\{0.33em}}M,N\}\right)\text{\textbackslash{}hspace\{0.33em}\}=\text{\textbackslash{}hspace\{0.33em}\}\sqrt{{\left(\frac{SE\left(\rho_{M,N}\right)}{\rho_{M,N}}\right)}^{\text{2}}+{\left(\frac{0.5\;h_{M}\;SE\left(h_{\;M}^{\text{2}}\right)}{h_{M}}\right)}^{\text{2}}+{\left(\frac{0.5\;h_{N}\;SE\left(h_{\;N}^{\text{2}}\right)}{h_{N}}\right)}^{\text{2}}}$$

#### Empirical derivation of a standard error (SE) correction factor for HE regression.

Derivation of a standard error correction factor for HE regression was performed using simulated phenotypes and populations. Simulations for each phenotype at each population size were repeated 1,000 times.

Phenotypes at varying *h*^*2*^ levels (0.01, 0.05, 0.1, 0.2, 0.4, 0.8, 0.99) were simulated for populations of 10,000 individuals via the ‘PhenotypeSimulator’ package in R [60]. Genotypes for each population were simulated at 1,000 SNPs. 10 SNPs were selected to be causal with independent, additive effects on their respective phenotype, with minor allele frequencies sampled from the following list of values: 0.05, 0.1, 0.3, 0.4) and were standardised as described in [62]. Kinship matrices were computed via the getKinship() function and values were divided by 2 to match kinship matrices computed via the ‘QTL2’ package in R [7,49], which computes the probability of sampling a particular allele among pairs of individuals.

For each simulated phenotype, the corresponding population was split randomly into two subpopulations consisting of 5,000 individuals. These subpopulations were then downsampled to varying population sizes ranging from 220 to 5,000 individuals. For each downsampled population, *h*^*2*^ was computed via Haseman-Elston regression and the standard error of the *h*^*2*^ estimate was computed as detailed above.

To assess whether these standard error estimates were appropriate, we utilized the approach taken by Ruby *et al.* in which *h*^*2*^ estimates and standard errors from each set of 1,000 simulated populations were used to generate expected and observed differences among the corresponding downsampled subpopulations [63], see supplemental text section 5.1-2, S2 Fig). These expected and observed differences were then used to generate a vector of ratios using the formula:


$$\;\Delta norm\;=\;\frac{h_{\text{1}}^{\text{2}}-h_{\text{2}}^{\text{2}}}{\sqrt{\sigma_{\text{1}}^{\text{2}}\;+\;\sigma_{\text{2}}^{\text{2}}}}$$

Where *∆norm* is the vector of expected:observed differences, *h*^*2*^_*1*_ and *h*^*2*^_*2*_ are the heritability estimates of the two downsampled subpopulations, and σ^*2*^_*1*_ and σ^*2*^_*2*_ are the variances about the heritability estimates of the two subpopulations (see [63], supplemental text equation 56). Repeated across a sufficiently large number of samplings, the standard deviation of *∆norm* values, σ_∆*norm*_, should be approximately 1, with values <1 or >1 signifying a greater or lesser precision of the SE estimate than the null expectation. σ_∆*norm*_ was used to assess the precision of HE standard errors across population size and *h*^*2*^.

After finding that standard error estimates decreased in precision (σ_∆*norm*_ > 1) as a function of both the *h*^*2*^ of the phenotype and the size of the population (S25 Fig), we utilized a linear regression based approach to find a correction factor, *F*, that would result in the expected precision of the standard error terms (σ_∆*norm*_ ~ 1) across phenotypes of varying *h*^*2*^ measured in populations of varying size. Across a series of heritability values (0.1, 0.2, 0.4, 0.8, 0.99), σ_∆*norm*_ increased linearly with the square root of the sample size (S26 Fig), and so the following linear model was used to fit σ_∆*norm*_ as a function of √n at each *h*^*2*^:


$$\sigma_{\Delta norm}\;\sim \;\alpha \;+\;\beta n\;+\;e$$

Where ɑ, β, and e are the intercept, slope, and error of the regression, respectively. Secondary linear regressions were then run that fit the resulting ɑ and β terms as a function of *h*^*2*^ (S27 Fig):


$$\alpha \;\sim \;\alpha \text{'}\;+\;\beta \text{'}h^{\text{2}}\;+\;e\mathrm{\backslash ]}$$

$$\beta \;\sim \;\alpha \text{''}+\;\beta \;\;\text{''}h^{\text{2}}\;+\;e\mathrm{\backslash ]}$$

The square root of the sample size, ɑ’, ɑ,” β’, and β” were then used to create a SE correction factor, *F*, that should result in SE estimates with the expected levels of precision at a particular *h*^*2*^ and sample size:


$$F\;=\;\sqrt{n}\;(\beta ’h^{\text{2}}\;+\;\alpha ’)\;+\;(\beta ’’h^{\text{2}}\;+\;\alpha ’’)\;$$

The empirically derived formula for F used in the estimation of SE of *h*^*2*^ and *r*_*g*_ estimates in this manuscript is:


$$F\;=\;sqrt(n)\;(0.020225\;h^{\text{2}}\;+\;0.004225)\;+\;(-0.2352h^{\text{2}}\;+0.9467)$$

And corrected SE estimates (*SE*_*corrected*_) are derived via:


$${SE}_{corrected}\;=SE\;x\;F\;$$

This correction factor was then applied to SE estimates for 4,529 experimental traits in DO mice that varied in both *h*^*2*^ and sample size. Each trait was split randomly into two samples and *∆norm* was computed. σ_∆*norm*_ for the set of traits was 1.160 (compared to 1.203 using uncorrected SE values), suggesting that SE estimates for traits in our dataset have an approximately expected level of precision in our data (S28 Fig). Considering only individual traits with relatively large sample sizes (n ≥ 800), σ_∆*norm*_ was 1.199 (compared to 1.320 using uncorrected SE values), suggesting that our correction factor reduces the loss of precision in SE estimates as sample size increases.

Estimation of confidence intervals for *h*^*2*^ via Haseman-Elston regression have been previously described [15]; however, this implementation differs slightly from the one described herein and the standard errors produced by this method were ~ 2.5x larger than those calculated using Fisher’s methods (S2 Fig), as in [17]. Bootstrapping using a method similar to that described in [17] produced standard error estimates in between ours and those derived using the methodology from [15] (S28C Fig); however, the bootstrapping procedure is slower than estimating SE via the aforementioned methods, preventing it from scaling efficiently for large numbers of trait pairs, and suffers from the same loss in accuracy as our SE methods at higher *h*^*2*^ and population sizes (S28D Fig).

#### Intra-trait genetic correlations.

Intra-trait genetic correlations were estimated for phenotypes measured in **D**ietary **R**estriction **i**n **DO** mice (DRiDO) study [18]. These phenotypes were measured annually (or bi-annually) for up to four years per animal as well as across five dietary groups (ad libitum, or “AL”; 20% and 40% caloric restriction, or ‘20CR’ and ‘40CR’; one and two day intermittent fasting, or ‘1D’ and ‘2D’).

To estimate the average intra-trait *r_g_* among yearly time points or diet groups, *r_g_* was estimated for each trait among each pair of time points or diets. For each trait with bi-annual measurements (denoted as ‘YearX_A’ and ‘YearX_B’), the first measurement was used. *r_g_* estimates greater than 1 or -1 by more than the standard error of the *r_g_* estimate (*r_g_* > 1 + se or *r_g_* < -1 - se) were excluded from the analysis. After intra-trait *r_g_* was estimated for all traits across all pairs of time points or diets, the mean and standard error of intra-trait *r_g_* for all traits was reported for each pair of time points or diets via heatmap in Fig 1C-1D. Individual yearly traits were used in the estimation of intra-trait *r_g_* across diets.

To assess patterns of intra-trait *r_g_* across trait categories, all pairwise *r_g_* estimates were calculated across all pairs of time points or diet groups. *r_g_* estimates greater than 1 or -1 by more than the standard error of the *r_g_* estimate (*r_g_* > 1 + se or *r_g_* < -1 - se) were excluded from the analysis. For each trait, the mean and standard error of its *r_g_* estimates among time points or diets was calculated and mean values are reported via violin plots in Fig 1E-1F and summarized in Fig 1G.

#### Hierarchical clustering of traits based on Pearson genetic correlations (*r_pg_*).

For each pair of traits, Pearson genetic correlations (*r_pg_*) were estimated using z-score normalized vectors of genetic correlations (*r_g_*) from the entire dataset of 7,233 high-confidence phenotypes, which included phenotypes from the DRiDO and lifespan studies that were derived from individual dietary groups or drug treatments [13,18]. High confidence phenotypes were defined as those with *h*^*2*^ estimates ≥ 0.1. Because Haseman-Elston regression can also produce *h^2^* estimates greater than 1 due to error associated with the slope of the regression (β), we also excluded any traits with *h*^*2*^ estimates that exceeded 1 by greater than their standard error estimate (*h*^*2*^ > 1 + se(*r*_*g*_) were excluded).

Because positive and negative genetic correlations are both indicative of a shared genetic basis, the absolute value of *r*_*pg*_, or |*r*_*pg*_|, was used for clustering analysis. To facilitate hierarchical clustering of traits, |*r*_*pg*_| values were converted to euclidean distance using the formula:


$$d\;=\;(-2n(|r_{pg}|-1))$$

Where d is the euclidean distance between the pair of traits, n is the number of observations – in this case, *r_g_* measurements– used to estimate *r*_*pg*_, and |*r*_*pg*_| is the absolute value of the Pearson genetic correlation estimate for the pair of traits.

Hierarchical clustering was performed on a matrix of squared distances among 1,898 high-confidence phenotypes (diet- and drug- specific phenotypes were excluded) using the “ward.D2” hierarchical clustering algorithm from the function hcut() from the R package “factoextra” [64]. The level of clustering, *k*, that maximized average *h*^2^ when performing meta-analysis of trait clusters, was used (see “Construction of meta-traits *from clustered phenotypes to maximize h*^*2*^*” below)*.

#### Determination of a level of hierarchical clustering to maximize *h^2^.*

Because *r*_*g*_ and *r*_*pg*_ indicate the degree to which pairs of traits share underlying genetic effects, traits with higher *r*_*g*_ or *r*_*pg*_ values should be more likely to arise from one or more of the same causal variants. Combining data from genetically correlated traits should increase the statistical power to detect shared QTL, which may increase the *h*^*2*^ estimate of the aggregated phenotype data in a mega-analysis. To test this, we combined 1,000 random pairs of traits (see Construction of meta-traits below) and estimated the *h*^*2*^ of the resulting meta-traits (*h*^*2*^_*meta*_). For each pair, we took a ‘heritability ratio’:


$$h^{\text{2}}ratio\;=\frac{\left(h_{meta}^{\text{2}}\right)}{0.5\;\times \;(h_{\text{1}}^{\text{2}}+h_{\text{2}}^{\text{2}})}\;$$

Where *h*^*2*^_*meta*_ is the heritability of a meta-trait resulting from the combination of two sets of trait data, *h*^*2*^_*1*_ is the heritability of the first combined trait, and *h*^*2*^_*2*_ is the heritability of the second. There was a highly significant correlation between the degree of shared genetic architecture (*|r*_*pg*_*|*) and the *h*^*2*^ ratio for the pairs of traits (r = 0.328, p = 1.38x10^-26^; S4 Fig), implying that a ‘heritability ratio’ could be used to determine an appropriate level of clustering in our data.

Heritability ratios were applied to the phenome-wide dataset (1,898 traits) to determine a level of clustering, *k*, that would maximize the average *h*^*2*^ when performing mega-analyses. At each possible level of hierarchical clustering (i.e., from 2 clusters of traits to 1,897 clusters of traits), *h*^*2*^ ratios were computed as follows:

At each level of clustering, *k*, we first cut the dendrogram to specify *k* clusters of traits. Within each cluster, a meta-trait was constructed by computing the mean of the z-score normalized phenotype values for each animal in the cluster. Next, *h*^*2*^_*meta*_ was estimated for each of the *k* meta-traits using HE regression. Each meta-trait comprises two sub-dendrograms, which represent the two sets of traits grouped together at a particular level of clustering; for each meta-trat, the *h*^*2*^ of its constituent sub-dendrograms, *h*^*2*^_*sub1*_ and *h*^*2*^_*sub2*_ were also estimated. The *h*^*2*^ ratio for each meta-trait was defined as *h*^*2*^_*meta*_ divided by the mean of *h*^*2*^_*sub1*_ and *h*^*2*^_*sub2*_, expressed as:


$$h^{\text{2}}ratio\;=\frac{\left(h_{meta}^{\text{2}}\right)}{0.5\;\times \;(h_{sub1}^{\text{2}}+h_{sub2}^{\text{2}})}\;$$

The mean *h*^*2*^ ratio was computed across all *k* meta-traits at a particular level of clustering. We selected a level of clustering that maximized the mean of the *h*^*2*^ ratio among all groups of traits, which should maximize our statistical power to detect genetic effects influencing meta-traits.

#### Construction of meta-traits.

Meta-traits were constructed from sets of clustered phenotypes at a particular level of clustering (see above) by taking the mean of the z-score normalized phenotype values within each trait cluster. Because traits were hierarchically clustered based on the absolute value of their Pearson genetic correlation (|*r_pg_*|), traits within particular clusters may be negatively genetically correlated with one another. To facilitate the construction of meta-traits from negatively correlated phenotypes, a “focal phenotype” was assigned from each cluster, defined as the trait with the highest *h^2^* estimate. Each other trait within a cluster was then compared to this focal phenotype; traits with negative *r_pg_* estimates were sign-flipped and traits with positive *r_pg_* estimates were not. After this processing, the average of the clustered phenotypes was taken as the meta-trait value for each animal. Animals lacking any phenotype measurements for a given trait cluster were assigned “NA”.

#### Additive whole-genome scans of meta-traits.

Genetic analysis was conducted using the “rqtl2” package in R [56,65]. Whole-genome scans were performed for each meta-trait using the scan1() function utilizing a mixed effects model in which a focal trait is regressed on 8-state allele probabilities for each individual. Because age, sex, DO generation wave, and intervention (diet or drug treatment, referred to as ‘Diet’ above) were already accounted for during the preprocessing of phenotype data for Haseman-Elston regression and subsequent meta-trait construction, no additive covariates were used in whole-genome scans. QTL were called using the find_peaks() function from “rqtl2” [56] with the following settings: ‘*drop’* = 2, ‘*peakdrop’* = 3, and ‘*threshold’* set to one of the two statistical thresholds described below. Approximate confidence intervals around each QTL were defined using a 2LOD interval around the sentinel marker, as previously described [3,66–68].

For each scan, a significance threshold of ⍺ = 0.05 was established using 1,000 unrestricted permutations of the data [69], with each permutation consisting of a whole-genome scan in which meta-trait values were randomized across all individuals. Typically, restricted permutation tests that account for relatedness are used to control the type I error rate when working with structured populations (e.g. MVNpermute) [70]. However, it is common to treat DO cohorts as a single, heterogeneous population and unrestricted permutation schemes are commonly utilized [3,66,67], even in DO studies involving multiple cohorts [71,72]. The maximum LOD score observed in each permutation was recorded, resulting in a distribution of 1,000 maximum LOD scores. The 95^th^ percentile of this distribution was used as the significance threshold (⍺ = 0.05) for the corresponding meta-trait. QTL with LOD scores greater than this threshold were considered significant at our permutation-based threshold. In addition to this significance level, a significance level of LOD ≥ 6 was also used to identify loci contributing to variance in lifespan. This LOD score corresponds to an average false-discovery rate (FDR) of 27.8% in the phenome-wide meta-traits; however, FDR and the number of loci discovered vary by meta-trait. Considering individual meta-traits, we expect 85.8%, 82.4%, and 78.1% of the total QTL discovered in our dataset to be true positives at LOD ≥ 6.

FDR for each whole-genome scan was determined using 1,000 permutations of the data. In each permutation, phenotype data was sampled without replacement and additive whole-genome scans were run as described above. In each permuted scan, loci were called using the find_peaks() function from “rqtl2” [56] across a range of LOD scores. These detections were considered false discoveries, and detections in the non-permuted data were considered true discoveries. At each LOD threshold, FDR was calculated using the following formula:


$$FDR\;=\frac{{QTL}_{False}}{{QTL}_{False}\;+\;{QTL}_{True}}\;$$

We report the average FDR at a particular LOD threshold across the 1,000 permutations of the data. The number of QTL expected to be “true positives” in a particular whole-genome scan is:


True positives =⌈FDR x QTL⌉ 

Or, the product of the permutation-derived FDR times the number of total discoveries, rounded up.

#### QTL effect size estimation and variance explained.

Best linear unbiased predictors (BLUPs) and standard errors were calculated for all QTL using the scan1blups() function in “rqtl2” on meta-traits without the use of additive covariates, as above [56]. The fraction of phenotypic variance explained by each QTL was calculated using the LOD score at the sentinel genotyping marker via the formula [73]:


$$1\;-\;10^{-(2/n)LOD}$$

Where n is the number of observations (i.e., animals) and LOD is the LOD score of the peak marker at each QTL. In specified instances involving low sample sizes, LOD scores from the sentinel SNP identified in variant association mapping, rather than the sentinel genotyping marker, was used to estimate the fraction of variance explained by a QTL.

#### Variant association/ fine mapping of QTL.

Variant association mapping was conducted within a 2LOD interval around the peak position of each QTL. Fine mapping was performed via the scan1snps() function in “rqtl2” [56] without additive covariates, as detailed in the section Additive whole-genome scans of meta-traits above. Variant and gene SQLite datasets used in this analysis are available at the “rqtl2” user guide website: https://kbroman.org/qtl2/assets/vignettes/user_guide.html.

#### Correlation of haplotype effects across trait clusters.

We used 2LOD support intervals around QTL to determine which QTL from meta-traits overlapped previously detected, reproducible lifespan QTL on chromosomes 12 and 18 from meta-trait C_all_252 (DRiDO and meta-lifespan), and chromosome 16 from meta-trait C_all_216 (Shock study lifespan).

For each lifespan QTL, the top 1% of significant SNPs from variant association mapping were identified. The nearest genotyping markers to each of these SNPs were defined as the “focal markers” for each lifespan QTL. The set of unique focal markers at each lifespan QTL were then used to compute Pearson correlations between effect sizes at lifespan QTL and overlapping QTL. Sets of highly correlated haplotype effects (p < 1x10^-10^) were prioritized for downstream analysis.

#### scRNA-seq expression in human protein atlas dataset.

QTL that both overlap lifespan QTL and have haplotype effects correlated with the effects of the lifespan loci were considered potential lifespan loci. The meta-traits in which these QTL were detected were used to determine cell types of interest that could be useful for refining lists of candidate genes within the chromosome 12 and 18 confidence intervals. All genes within the 2LOD support intervals of the lifespan QTL on chromosomes 12 and 18 were recorded, and the expression levels of these genes within relevant cell types were examined using single-cell RNA seq (sc-RNAseq) data from the Human Protein Atlas [25,26].

sc-RNAseq data were downloaded from the Human Protein Atlas website at:

https://www.proteinatlas.org/humanproteome/single+cell/single+cell+type/data#cell_type_data

#### Correlation of haplotype effects at cardiac eQTL.

An external transcriptomic dataset derived from the cardiac tissue of DO mice was used to identify *cis*-eQTL overlapping with QTL detected in meta-analysis of aorta phenotypes [32]. This dataset was accessed via the Jackson Laboratory QTL Viewer website at https://churchilllab.jax.org/qtlviewer/JAC/DOHeart. For each aorta QTL, genes with *cis*-eQTL and starting positions within 2Mb of the sentinel marker were recorded. Pearson correlation coefficients were computed between the haplotype effects (BLUPs) at the peak positions of the aorta QTL and any nearby *cis*-eQTL. BLUPs were not available at each genomic marker within the external dataset, so correlations were based only on the haplotype effects at peak markers rather than across all significantly associated markers within the 2LOD support intervals of the QTL. Correlation coefficients with p < 0.1 were considered significant in our analysis.

This procedure was performed using proteomic data from the same external dataset, but no significant *cis*-pQTL were detected within 2Mb of the aorta QTL from this study.

#### Open field and frailty assays.

We implemented an open field system that would be compatible with the varied coat colors of DO mice. Details about this system, including instructions for assembly, use, and source code for the analysis of video data, are provided in this GitHub repository: https://github.com/graham-calico/OpenFieldAssay. For the phenotypes reported here, videos were collected and analyzed for 10 minutes after the mouse was placed in the box. Phenotypes were generated by the openFieldAnalysis.py script using the following non-default command-line options: “-b best --adjust_fps”.

All MFI data analyzed here are from [24], as are many of the analyzed DFI data. Additional DFI measurements reported here were collected as described by [24].

#### Aorta histology.

There were a total of 138 Diversity Outbred mouse aortas collected across 9 aged cohorts: 11 mo (n = 26), 14 mo (n = 24), 17 mo (n = 15), 20 mo (n = 11), 23 mo (n = 8), 25 mo (n = 5), 29 mo (n = 20), and 32 mo (n = 29). The whole aorta was removed, stripped of adherent tissue, then cut into four specific segments labeled with different colored dye for identification. Four segments: the ascending aorta, was cut from the base of the aorta connected to the heart and separated before the brachiocephalic trunk; the aortic arc, start at the brachiocephalic trunk and cut at the left subclavian artery; the descending aorta, start at the end of the left subclavian artery and cut at 6mm then split into two (proximal & distal) 3mm aortic segments. Each tissue was embedded into paraffin blocks and sectioned for Hematoxylin & Eosin (H&E) stain, Verhoeff van Gieson (VvG) stain, and Mason Trichrome (Trichrome) stain.

#### Lung histology.

Female C57BL/6J and DO mice were obtained from the Jackson Laboratory and allowed to acclimate for 2–4 weeks. Lungs were perfused with sterile PBS and inflated with 1.5% low melting point agarose (Invitrogen) at a pressure of 25 cm H_2_O. Lungs were excised and fixed in 10% neutral buffered formalin for 24 hours at room temperature. Left lobes were processed for histology (serial H&E and Trichrome collagen stains at 6–8 positions in the lobe with a 200 um distance between positions).

#### Micro-CT imaging of lung tissue.

To assess lung structure, mice were anaesthetized with isoflurane (1–3%) in medical air. Isoflurane levels were adjusted to maintain a breathing rate of 50–60 breath per minute. Focused lung scans with respiratory gating were acquired with a MILabs µCT system (U-CT, MILabs, Utrecht, Netherlands) using the following settings: mouse ultra focus protocol, X-ray voltage 60 kV, current 160 µA, exposure 50 ms, binning of 2X2, 0.5 degrees step angle, 6 projections per angle. Micro-CT images were reconstructed using CTRecon software (Gremse-IT GmbH, Germany) at 50 µm isotropic resolution with retrospective respiratory gating. C57BL/6J and the DO mice were imaged once. Radiation delivered per scan was less than 1 Gy and imaging time is approximately 6 min.

Lungs were automatically segmented using a machine learning (U-Net) model trained on manual segmentations of lungs [74]. After segmentation, lung masks were registered (rigid followed by affine) to a reference atlas using Elastix (Version 5.2) [75,76]. Masks for different lobes (Left, right cranial, right middle, right caudal and right accessory) were propagated to the original image space and used for lobe specific analysis. Image intensity thresholds were defined for fibrosis or inflammation (> -250 HU) and emphysema (<-750 HU). Different lung parameters like lung volume, high signal intensity volume “fibrosis volume”, emphysema volume, percentage of lung affected by fibrosis or emphysema were computed for each lobe.

#### Oxygen transfer measurements.

Carbon monoxide diffusion measurements is a commonly used technique to measure lung function (transfer rate from alveoli to the blood stream). A surrogate method has been developed to estimate oxygen transfer rates based on changes in blood oxygen saturation (spO2) when animals are breathing gas mixtures containing 21% or 7% oxygen. Mice were anesthetized with 1.5% isoflurane, transferred to a heating pad to maintain body temperature at 37°C and maintained on 0.75% isoflurane. A rectal temperature probe was used to monitor body temperature and pulse oximeter clip (MouseOxPlus, Starr Life Sciences, PA, USA) positioned on the thigh on which hair removal had been performed on the previous day using a depilatory cream. A breathing pad was attached to calculate the breathing rate. Mice were subject to hypoxia at 7% oxygen + 93% nitrogen for 2 minutes followed by normoxia at 21% oxygen + 79% nitrogen for 2 minutes. This exposure pattern was repeated three times. Temperature, breathing rate, heart rate and oxygen saturation were being recorded continuously during the experiment. Linear fits were performed for blood oxygen saturation during the switch from hypoxia to normoxia. We define the slope of this fit as oxygen transfer rate.

#### Lung mechanics measurements.

Mechanical lung parameters were measured with the flexiVent (FX, SCIREQ, Montreal, QC, Canada) system. Mice were anesthetized with ketamine (70 mg/kg) and xylazine (8 mg/kg). A 20G cannula was inserted intratracheally and connected to the flexiVent while the mice were placed in a supine position. Mice were mechanically ventilated with a tidal volume of 10 mL/kg at a breathing frequency of 150 breaths/min and with a positive end-expiratory pressure of 3 cmH_2_O. After checking for leaks and once the ventilation was underway, mice were paralyzed by injecting of rocuronium (2 mg/kg) intraperitoneally, to avoid spontaneous breathing during the procedure. Lung mechanics measurements were made using the flexiVent system’s automated algorithms and were repeated three times.

A Deep Inflation maneuver inflated the lungs to maximum capacity at a pressure of 30 cmH_2_O, maintained over a time period of 3 s, to measure the inspiratory capacity (IC). To calculate total respiratory system resistance (R_rs_), compliance (C_rs_), and elastance (E_rs_), a single-frequency (SnapShot-90) perturbation was used in alignment with mice respiratory rate and tidal volume. Impedance based measurements like newtonian resistance (R_n_), tissue damping (G), and tissue elastance (H) was obtained by running a broadband (Quick Prime-3) forced oscillation perturbation which uses a range of frequencies above and below the respiratory rate. Inspiratory capacity (A), curvature of the deflating PV loop (K), and quasi-static compliance (C_st_) were measured using stepwise pressure-controlled pressure–volume (PVs-P) loops. The forced expired volume over 0.1 s (FEV0.1) was obtained by performing the The negative pressure-driven forced expiratory (NPFE) maneuver by inflating the mouse lungs to a pressure of 30 cm H_2_O for 1 s, and holding that pressure for a period of 2 seconds followed by connecting the animal’s airways to the negative pressure reservoir (− 55 cm H_2_O) for 2 seconds. Data was excluded if the model fit was poor (coefficient of determination less than 0.9) for every model.

#### Histological image preprocessing and ROI extraction.

We developed automated computational pipelines for quantitative analysis of histological images, tailored to specific staining techniques and tissue types. These pipelines facilitate efficient handling of large whole-slide images, extraction of regions of interest (ROIs), and quantification of morphological and structural features pertinent to our study. The detailed methodology for image preprocessing and ROI extraction is extensively described in our separate publication [77]; here, we briefly summarize the key steps and specific analyses performed. Whole-slide images were downsampled to reduce computational load while preserving sufficient detail for accurate region detection. Optical density (OD) images were computed to enhance contrast between stained tissue and background. Background masking and adaptive thresholding were applied to segment tissue regions effectively. Morphological operations refined the tissue masks, and connected-component analysis identified distinct ROIs defined by bounding boxes. The downsampling scaling factor was recorded to ensure precise mapping between the downsampled images and the original resolution.

#### Analysis of aortic elastic laminae in VVG-stained images.

We quantified elastin structures within the aortic wall from Verhoeff–Van Gieson (VVG)-stained images, assessing parameters such as elastin layer thickness, continuity, waviness (tortuosity), and the number of elastin layers. The pipeline for VVG analysis is extensively detailed in our separate publication [77]. Here, we briefly summarize the pipeline. High-resolution images were processed to extract ROIs based on predefined bounding boxes. Color deconvolution isolated elastin staining in OD space, separating VVG stain components and removing artifacts. Elastin masks were created by thresholding the OD image, followed by morphological operations to refine the mask. Skeletonization reduced fibers to their centerlines for structural analysis. A network representation was constructed, classifying nodes as tip points, branch points, or regular points, and metrics like network complexity were calculated. Elastin thickness was measured using a distance transform sampled at skeleton pixels, providing a pixel-wise representation across the tissue. Waviness was quantified by calculating tortuosity along fiber paths, comparing curved distances to straight-line distances. The number of elastin layers was determined by analyzing the elastin mask and counting distinct components in horizontal and vertical directions.

#### Quantification of aortic cellular metrics in HE-stained images.

In Hematoxylin and Eosin (HE)-stained images, we quantified cellular metrics by detecting and segmenting nuclei to assess cellularity. Whole-slide images were read using the tifffile library, and ROIs were extracted based on predefined bounding boxes. Color deconvolution separated the hematoxylin and eosin stain contributions. A background mask excluded non-tissue areas through intensity thresholding, color ratio calculations, Gaussian filtering, and morphological operations. Tissue regions were identified by thresholding OD images. Nuclei detection and segmentation involved thresholding the smoothed OD channels, combining masks, and using distance transforms and watershed segmentation. Small objects were removed to eliminate noise, and connected components were labeled to identify individual nuclei. Cellular metrics such as the number of cells per tissue area and cell area per tissue area were calculated, providing insights into cell density and tissue occupancy by cells.

#### Analysis of aortic wall metrics in trichrome-stained images.

We quantified collagen density within the aortic wall, wall thickness, external collagen lining thickness, and perimeters of the aorta in trichrome-stained images. Whole-slide images were read, and ROIs were extracted. Color deconvolution separated the staining components specific to trichrome staining. Collagen masks were generated by thresholding the appropriate OD channel after Gaussian filtering. An aortic wall mask was created by summing OD channels, applying Gaussian filtering, thresholding, and refining with morphological operations. The collagen mask and aortic wall mask were combined to identify collagen within the aortic wall, and the fraction of collagen was calculated by the overlap area. Thickness measurements were obtained using distance transforms and skeletonization of the masks, providing detailed measurements across the tissue. Perimeters were calculated using connected-component analysis and morphological operations. Statistical analyses of thickness maps included metrics such as mean, standard deviation, coefficient of variation, and skewness.

#### Quantification of alveolar structures and nuclear metrics in HE-stained lung images.

In HE-stained lung images, we quantified alveolar structures and nuclear metrics to assess lung tissue architecture and cellularity. Images were downsampled for computational efficiency. A tissue mask was generated from grayscale images using adaptive thresholding and morphological operations. Alveolar structures were detected using multi-scale Laplacian or Gaussian filters, and alveolar centers were identified from local maxima in the distance-transformed image. Alveolar radii were measured by averaging distances from centers to the nearest tissue borders. Nuclei were detected via color deconvolution to extract the hematoxylin channel, followed by Gaussian filtering and thresholding. Local maxima identified individual nuclei, and nearest neighbor distances provided information on nuclear distribution and density. Cluster analyses were conducted to assess nuclear clustering based on proximity and density.

#### Quantification of collagen content in trichrome-stained lung images.

We quantified collagen content in trichrome-stained lung images by generating tissue masks and isolating collagen-specific stains via color deconvolution. Collagen masks were created by thresholding the appropriate OD channel after Gaussian filtering. Vessel regions were excluded using a vessel mask generated through image filtering and thresholding. Quantitative metrics were derived by calculating the areas occupied by tissue and collagen within the lung tissue mask. Ratios representing the proportion of lung tissue occupied by collagen were calculated by dividing the collagen area by the sum of tissue and collagen areas.

#### Histological image data analysis and validation.

Quantitative metrics from each pipeline were compiled into structured data formats and saved as CSV files for statistical analysis. A thread-safe writing mechanism ensured data integrity during concurrent processing. Optional validation images were generated to facilitate visual inspection of processing results, including overlays of masks and thickness maps on original images. All image processing operations utilized established libraries such as NumPy, SciPy, and scikit-image. Our custom-built histo-tools library was used for stain deconvolution, providing robust and efficient implementations of the required algorithms. This approach ensured consistent and efficient processing across multiple subsamples and slides, enabling large-scale quantitative analysis of histological images.

#### In vivo assays and husbandry.

All research was performed as part of Calico Life Sciences LLC (South San Francisco, CA) AAALAC-accredited animal care and use program. All research and animal use in this study was approved by the Calico Institutional Animal Care and Use Committee.

## Supporting information

S1 Fig Haseman-Elston regression for estimation of genetic correlations (*r*_*g*_). **A,** Individual phenotype data is collected for two traits (‘A’ and ‘B’). Distributions of traits are z-score normalized and a vectorized outer product is taken by multiplying each measurement of trait A by each measurement of trait B. The result is a set of pairwise phenotypic similarity scores from all combinations of individuals. **B,** Phenotypic similarity scores for each pair of individuals are regressed on pairwise kinship values. The coefficient of the regression can be used to determine the covariance of traits A and B. **C,** A structural equation model relates the covariance of traits A and B as a function of relatedness to the genetic correlation (*r*_*g*_) of the two traits. The *h^2^* and covariance of traits A and B must be known in order to solve for *r*_*g*_. **D,** Haseman-Elston *h*^2^ estimates for simulated phenotypes. Mean Haseman-Elston *h*^*2*^ estimates and 95% confidence intervals of 1,000 randomly simulated traits at different sample sizes. Actual *h*^*2*^ of the simulated traits is shown at the top of each panel.
(PDF)

S2 Fig A comparison of Haseman-Elston implementations. Comparison of *h^2^* estimates and standard errors for 34 DO mouse traits derived from our implementation of Haseman-Elston regression and the implementation of Haseman-Elston regression described in [15]. The Pearson correlation coefficient and accompanying p-value are reported in the upper-right of the plot area.Traits are colored by type: ‘AS’ - Acoustic Startle. ‘DX’ - DEXA scan. ‘EC’ - Echocardiogram. ‘GS’ - Grip Strength. ‘RR’ - Rotarod. ‘WR’ - Wheel Running.
(PDF)

S3 Fig Differences between Haseman-Elston and REML *r*_*g*_ estimates are driven by *h*^2^ of trait pairs. **A,** Differences in pairwise *r_g_* estimates between Haseman-Elston regression and REML as a function of the mean *h*^2^ of the traits being compared. **B,** Differences in pairwise *r*_*g*_ estimates between Haseman-Elston regression and REML as a function of the minimum *h*^2^ of the traits being compared. **C,** Standard error of Haseman-Elston based *r*_*g*_ estimates as a function of the mean *h*^2^ of the traits being compared. **D,** Standard error of Haseman-Elston based *r*_*g*_ estimates as a function of the minimum *h*^2^ of the traits being compared.
(PDF)

S4 Fig Mean inter- and intra-study *r*_*pg*_ estimates. Mean *r*_*pg*_ estimates among traits from each study. Values derived from a single trait pair are represented by gray boxes. ‘Calico internal’ traits comprise open field, frailty, lung, and aorta traits, including histology-based phenotypes.
(PDF)

S5 Fig Heritability of aggregated trait pairs increases relative to individual heritability estimates as a function of *r_pg_.* Scatterplot of the *h^2^* ratio for 1,000 random pairs of traits as a function of the absolute value of their *r_pg_*. The *h^2^* ratio for traits A and B is defined as the *h^2^_meta_*/ mean(*h^2^_A_*,*h^2^_B_*), where *h^2^_meta_* is the heritability of a meta-trait constituting traits A and B, and *h^2^_A_* and *h^2^_B_* are the individual heritabilities of traits A and B. The Pearson correlation coefficient and p-value between the *h^2^* ratio and |*r_pg_*| are reported.
(PDF)

S6 Fig Genetic correlations among 383 meta-traits. Hierarchically clustered heatmap of *r*_*g*_ (*lower triangle*) and *r*_*pg*_ (*upper triangle*) estimates among 383 DO meta-traits. The accompanying dendrogram shows euclidean distances among meta-traits based on their *r*_*pg*_ estimates.
(PDF)

S7 Fig Average false discovery rate (FDR) by LOD score for phenome-wide meta-traits. The solid vertical line corresponds to the average genome-wide significance threshold of ⍺ = 0.05 based on 1,000 permutations of the data, performed separately on each meta-trait. The dashed vertical line corresponds to the LOD-based threshold of 6.
(PDF)

S8 Fig rpg estimates between individual lifespan studies and meta-traits. The strongest positive and negative *r_pg_* estimates between meta-traits and lifespan data measured in **A,** the DRiDO study, **B,** the Harrison study, and **C,** the Shock study. Error bars correspond to the standard error of each *r_pg_* estimate. Meta-trait number (cluster number) is shown on the left hand side of the plot. The traits comprising each cluster can be found in S6 Table.
(PDF)

S9 Fig Genetic correlations among diet-specific lifespan measurements. **A,** Plot of mean *h^2^* ratios among clusters of diet- or drug-specific lifespan measurements at different levels of hierarchical clustering (k). **B,** Hierarchically clustered heatmap of *r_g_* (*lower triangle*) and *r_pg_* (*upper triangle*) among lifespan measurements within individual dietary or drug intervention groups. Statistically significant estimates after correcting for phenome-wide multiple testing are denoted by an asterisk.
(PDF)

S10 Fig Additive whole-genome scan on lifespan measurements from the Shock study. Solid red lines indicate a trait-specific and permutation-based genome-wide significance threshold. Dashed red lines indicate a nominal significance threshold of LOD > 6.
(PDF)

S11 Fig Phenotypic correlations among frailty traits. **A,** Phenotypic correlations between open field traits and mean digital frailty index scores (DFI), mean manual frailty index scores (MFI), and age. Statistically significant correlations are denoted by an asterisk. **B,** Phenotypic correlations among open field, DFI, and MFI traits. **C**.
(PDF)

S12 Fig A histological analysis of aorta structure in DO mice. 116 DO mice were genotyped and their aortas were harvested. Tissues were sectioned, stained with HE, TC, or VVG, and imaged.
(PDF)

S13 Fig *h^2^* ratio by clustering level for aorta phenotypes. Plot of mean *h^2^* ratios among clusters of aorta meta-traits at different levels of hierarchical clustering (k).
(PDF)

S14 Fig Genetic mapping of additional aorta meta-traits. Manhattan plots of additive whole-genome scans on aggregated aorta phenotypes: **A,** Aorta area. **B,** Cells per area. **C,** External collagen thickness. **D,** Cell area and internal collagen. Solid red lines indicate a trait-specific and permutation-based genome-wide significance threshold. Dashed red lines indicate a nominal significance threshold of LOD > 6.
(PDF)

S15 Fig Average false discovery rate (FDR) by LOD score for aorta meta-traits. The solid vertical line corresponds to the average genome-wide significance threshold of ⍺ = 0.05 based on 1,000 permutations of the data, performed separately on each meta-trait. The dashed vertical line corresponds to the LOD-based threshold of 6.
(PDF)

S16 Fig Haplotype effects of elastin QTL. Allelic effects (BLUPs) and corresponding standard errors of the loci detected in genome-wide scans of elastin meta-traits. **A,** Elastin thickness and percent area. **B,** Elastin breakage and tortuosity. **C,** Number of elastin layers.
(PDF)

S17 Fig Finemapping of elastin QTL with correlated allelic effects in cardiac cis-eQTL. **A-E,** Variant association mapping plots for each elastin QTL at which the allelic effects were significantly correlated with at least one cis-eQTL in an external transcriptomic dataset from cardiac tissue in aging mice. (*Top*) The LOD scores for each variant within the 2 LOD support intervals around each locus in the meta-analysis. The most likely candidate SNPs are highlighted in pink. (*Bottom*) Genes within the 2 LOD support intervals are depicted, with the exception of panel **C**, which depicts the genes underlying the most likely SNPs.
(PDF)

S18 Fig Additional allele effect correlations for additional elastin QTL. **A-D,** Correlations of haplotype effects between the peak markers at QTL influencing elastin phenotypes and *cis*-eQTL in an external DO cardiac tissue transcriptomic dataset. Candidate genes are highlighted with red text.
(PDF)

S19 Fig DO mice as a model for lung structure and function. Study design for the assessment of lung properties in mice. 116 DO mice (and 124 C57BL/6J) animals were genotyped and participated in a phenotyping pipeline that included µCT scans of the lungs and functional measurements. Upon exit from the study, lung tissue was harvested and fixed, and tissue sections were stained (TC or HE) and imaged.
(PDF)

S20 Fig Phenotypic correlations and *h*^*2*^ of lung phenotypes in C57BL/6J and DO mice. **A,** Phenotypic correlations among hierarchically clustered lung phenotypes in DO (*upper triangle*) and C57BL/6J (*lower triangle*) mice. Clusters were determined via silhouette score; phenotype names and the accompanying dendrogram are colored by cluster. **B**, Pairwise phenotypic correlations among pairs of lung traits plotted as a function of their correlation in C57BL/6J mice. **C,** Heritability estimates and standard errors of each lung phenotype prior to filtering. The dashed red line indicates the threshold of *h*^*2*^ < 0.1 at which traits were excluded from downstream analysis.
(PDF)

S21 Fig *h^2^* ratio by clustering level for lung phenotypes. Plot of mean *h*^2^ ratios among clusters of lung meta-traits at different levels of hierarchical clustering (k).
(PDF)

S22 Fig Genetic mapping of additional lung meta-traits. Manhattan plots of additive genome-wide scans for additional meta-traits composed of clustered lung phenotypes: **A**, Tissue area, alveolar radii, and cell density. **B,** Body weight and breathing rate under hypoxic conditions. **C,** Emphysema as measured via µCT imaging. **D,** Lung elasticity and capacity. **E,** Lung volume. **F,** Mouse registration and lung tumor scores. Solid red lines indicate a trait-specific and permutation-based genome-wide significance threshold. Dashed red lines indicate an additional threshold of LOD > 6.
(PDF)

S23 Fig Average false discovery rate (FDR) by LOD score for lung meta-traits. The solid vertical line corresponds to the average genome-wide significance threshold of ⍺ = 0.05 based on 1,000 permutations of the data, performed separately on each meta-trait. The dashed vertical line corresponds to the LOD-based threshold of 6.
(PDF)

S24 Fig µCT-derived tissue density phenotypes are not correlated with collagen levels and may reflect pulmonary inflammation or tumorigenesis. **A,** Fraction of lung tissue area stained for collagen via trichrome (TC) staining as a function of age in C57BL/6J mice. **B,** Percent area of the lung categorized as “high density” via µCT scan in C57BL/6J mice. **C,** Pixel intensity of “high density” tissue in µCT scans in C57BL/6J mice. **D,** Fraction of lung tissue area stained for collagen via TC staining as a function of age in DO mice. **E,** Percent area of the lung categorized as “high density” via µCT scan in DO mice. **F,** Pixel intensity of “high density” tissue in µCT scans in DO mice. **G-H,** Percent area of the lung categorized as “high density” via µCT scan as a function of (**G**) percent tissue area stained for collagen via TC stain, (**H**) the cellular density in the tissue via H&E (HE) staining, and (**I**) the number of abnormal lesions in the tissue in DO mice. The Pearson correlation coefficients and corresponding p values are reported above each plot. The solid lines are lines of identity and the dashed lines are best-fine lines from linear models.
(PDF)

S25 Fig Precision of Haseman-Elston *h*^2^ standard error estimates decreases as a function of *h*^2^ and sample size. Standard deviation of σ_∆*norm*_ values for simulated phenotypes across a range of *h*^*2*^ and population sizes. Values less than one indicate that the standard error of *h*^*2*^ for the simulated phenotypes is greater than expected. Values greater than one indicate that the precision of the standard error of *h*^*2*^ estimates is less than expected.
(PDF)

S26 Fig Linear relationship between σ_∆*norm*_ and √n of simulated traits. Linear regressions of σ_∆*norm*_ values for simulated phenotypes across different population sizes (n) at **A,** *h*^*2*^ = 0.1, **B,** *h*^*2*^ = 0.2, **C,** *h*^*2*^ = 0.4, **D,** *h*^*2*^ = 0.8, **E,** *h*^*2*^ = 0.99. The coefficients and *R*^2^ of each linear regression are reported in the upper left of each panel.
(PDF)

S27 Fig The relationship between coefficients from linear modeling of σ∆_*norm*_ and *h^2^.* The **A,** slope and **B,** intercept terms from linear regressions of σ_∆*norm*_ on √n plotted as a function of *h^2^*, demonstrating how standard error estimates deviate from expected precision as a function of the *h*^*2*^ of the examined phenotype. The coefficients and *R*^2^ of each linear regression are reported in the upper left of each panel.
(PDF)

S28 Fig Precision of corrected standard errors from Haseman-Elston *h^2^* estimates for DO mouse phenotypes. **A,** *∆norm* values for 1,898 traits in DO mice using corrected standard error estimates are plotted as a function of trait h^2^. **B,** The mean and standard deviation of *∆norm* values using corrected and uncorrected standard error estimates for all traits (*left*) and traits with larger sample sizes (*right*). **C,** Differences in the closed-form (*y-axis*) and analytic (*x-axis*) standard errors for *h*^*2*^ and the standard errors produced via bootstrapping as in [17]. Dashed lines indicated the mean difference among a set of 34 diverse traits measured in DO mice. **D,** Standard deviation of bootstrapped σ_∆*norm*_ values for simulated phenotypes across a range of *h*^*2*^ and population sizes.
(PDF)

S1 Table Normalized DO mouse phenotypes. Contains Mouse IDs, covariates used in phenotype processing, and z-score normalized phenotype values. ‘Mouse_ID’ is a unique identifier corresponding to each unique animal and includes study information. ‘Age’ is a covariate corresponding to the age of each animal at the time of phenotyping. For certain animals, different phenotypes were collected at different ages; for these animals, ‘Age’ is recorded as “variable”. ‘Sex’ corresponds to the sex of the animal (‘F’ for female and ‘M’ for male). ‘Diet’ corresponds to the dietary intervention group for each animal in the DRiDO study [18], but may also correspond to the number of days on a high fat diet (animals from the Svenson high-fat diet study, denoted as ‘Sven_HFD’ [Gatti et al. 2017]) or pharmacological intervention (e.g., lifespan data from [78]). ‘Genwave’ corresponds to the DO generation of each animal. Additional columns correspond to various z-score normalized phenotypes after correction for the aforementioned covariates. Phenotype names contain a prefix corresponding to the study from which they were collected: ‘attie’ (pancreatic phenotypes from [53]), ‘bone’ (bone strength phenotypes from [23]), ‘cal_cr’ (DRiDO phenotypes from [18]), ‘cal_int’ (newly published frailty, aorta, and lung phenotypes), ‘catnap’ (metabolic cage data from [11]), ‘ches_striatum’ (phenotypes from [20]), ‘lifespan’ (lifespan data from independent lifespan studies analyzed in [78]; ‘shock’ and ‘har’ correspond to the Shock and Harrison studies, respectively), ‘pazdro_heart’ (cardiac phenotypes from [22]), and ‘Sven_HFD’ (various phenotypes from [21]). Phenotypes may also contain a suffix corresponding to dietary intervention groups from the DRiDO study: ‘AL’ (*ad libitum*), ‘20’ (20% caloric restriction), ‘40’ (40% caloric restriction), ‘1D’ (one-day intermittent fasting), ‘2D’ (two-day intermittent fasting). Phenotypes from the DRiDO study collected annually or bi-annually will contain timepoint information corresponding to the year of the study in which they were collected (‘Y1’ - ‘Y4’). Biannual measurements are labeled with ‘A’ or ‘B’, corresponding to the first or second annual time point.
(ZIP)

S2 Table Data sources for DO mouse phenotypes. Contains information on the studies from which phenotypes were obtained. This includes the number of animals used and phenotypes measured, the general focus of each study, the source of the phenotype and genotype data, the genotyping array used, and the prefix assigned to corresponding Mouse_ID and phenotype labels.
(XLSX)

S3 Table Heritability (*h*^*2*^) of individual traits. Individual heritability estimates for each of the phenotypes in the dataset, before filtering based on *h*^*2*^. ‘Trait’ corresponds to one of the phenotypes in S1 Table. ‘h2’ provides the heritability of the trait as estimated by Haseman-Elston regression. ‘SE’ corresponds to the standard error of the trait. ‘p’ corresponds to the p-value of the *h*^*2*^ estimate. ‘n’ corresponds to the number of animals used in the estimate.
(TXT)

S4 Table Hierarchically clustered matrix of genetic correlations (*r*_*g*_). Row and column names correspond to the DO phenome after filtering by *h*^*2*^. This matrix includes DRiDO phenotypes split by diet group.
(RDATA)

S5 Table Hierarchically clustered matrix of Pearson genetic correlations (*r*_*pg*_). Row and column names correspond to the DO phenome after filtering by *h*^*2*^. This matrix includes DRiDO phenotypes split by diet group.
(RDATA)

S6 Table Meta-trait composition. Lists 1,898 phenotypes, filtered by *h*^*2*^ and excluding diet-specific DRiDO phenotypes. The ‘Cluster’ column specifies the meta-trait each trait comprises.
(TXT)

S7 Table Meta-traits constructed from clustered individual phenotypes. Meta-phenotype values for each animal in the dataset. Animal IDs are listed in the ‘Mouse_ID’ column, with each subsequent column corresponding to a meta-trait. IF none of the traits comprising a meta-trait were measured in a particular animal, the trait value for that animal is ‘NA’.
(TXT)

S8 Table Heritability of meta-traits. Heritability estimates for each of the meta-phenotypes in the dataset, excluding meta-traits composed of lifespan data from the Ellison study (prefixed with “lifespan_ell”, which were not significantly heritable and excluded from analysis). ‘Trait’ corresponds to a meta-trait, which is composed of individual phenotypes listed in S7 Table. ‘h2’ provides the heritability of the trait as estimated by Haseman-Elston regression. ‘SE’ corresponds to the standard error of the trait. ‘p’ corresponds to the p-value of the *h*^*2*^ estimate. ‘n’ corresponds to the number of animals used in the estimate.
(TXT)

S9 Table Genetic correlations among meta-traits. Pairwise genetic correlation estimates for each of the meta-phenotypes in the dataset, excluding meta-traits composed of lifespan data from the Ellison study (prefixed with “lifespan_ell”, which were not significantly heritable and excluded from analysis). Each row contains the following information: the pair of traits (‘Trait1’ and ‘Trait2’, respectively), the *r*_*g*_ estimate (‘rg’), the standard error of *r*_*g*_ (‘SE’), the p value associated with the Haseman-Elston regression (‘rg_p’), the Pearson correlation coefficient of the phenotypes (‘r’), the p value associated with the Pearson correlation coefficient (‘r_p’), the *h*^*2*^ estimates for each trait (‘h2_1’ and ‘h2_2’), and the number of animals in which the traits were measured (‘n_1’ and ‘n_2’).
(TXT)

S10 Table Hierarchically clustered matrix of genetic correlations among meta-traits. Row and column names correspond to the meta-trait after removing two meta-traits assembled from low *h*^*2*^ lifespan estimates.
(TXT)

S11 Table Hierarchically clustered matrix of Pearson genetic correlations among meta-traits. Row and column names correspond to the meta-trait after removing two meta-traits assembled from low *h*^*2*^ lifespan estimates.
(TXT)

S12 Table QTL detected in meta-traits at permutation-based significance levels. For each QTL, the following information is recorded: the meta-trait associated with the locus (‘Trait’), the chromosome (‘Chr’) and position information (‘Pos’, in megabases), the LOD score (‘LOD’), and the bounds of the 2LOD support interval (‘CI_start’ and ‘CI-end’).
(TXT)

S13 Table QTL detected in meta-traits at LOD ≥ 6. For each QTL, the following information is recorded: the meta-trait associated with the locus (‘Trait’), the chromosome (‘Chr’) and position information (‘Pos’, in megabases), the LOD score (‘LOD’), and the bounds of the 2LOD support interval (‘CI_start’ and ‘CI-end’).
(TXT)

S14 Table False discovery rates (FDRs) for each phenome-wide meta-trait at LOD ≥ 6 and α = 0.05 significance thresholds. For each meta-trait (‘Trait’), FDR rates are listed at the permutation-based ⍺ = 0.05 (‘alpha_05_FDR’) and LOD ≥ 6 (‘LOD6_FDR’) genome-wide significance thresholds.
(TXT)

S15 Table Frailty meta-trait composition. Lists each of the frailty phenotypes after filtering by *h*^*2*^. The ‘Cluster’ column specifies the meta-trait each trait comprises.
(TXT)

S16 Table Aorta meta-trait composition. Lists each of the aorta phenotypes after filtering by *h*^*2*^. The ‘Cluster’ column specifies the meta-trait each trait comprises. Trait suffixes correspond to the type of stain from which the measurement was derived: H&E (‘_he*’),* trichrome (‘_tc’), or Verhoeff-Van Gieson (VVG; ‘_vvg’).
(TXT)

S17 Table False discovery rates (FDRs) for each aorta meta-trait at LOD ≥ 6 and α = 0.05 significance thresholds. For each meta-trait (‘Trait’), FDR rates are listed at the permutation-based ⍺ = 0.05 (‘alpha_05_FDR’) and LOD ≥ 6 (‘LOD6_FDR’) genome-wide significance thresholds.
(TXT)

S18 Table QTL detected in aorta mega-analysis at LOD ≥ 6. For each QTL, the following information is recorded: the meta-trait associated with the locus (‘Trait’), the chromosome (‘Chr’) and position information (‘Pos’, in megabases), the LOD score (‘LOD’), and the bounds of the 2LOD support interval (‘CI_start’ and ‘CI-end’).
(TXT)

S19 Table Lung meta-trait composition. Lists each of the frailty phenotypes after filtering by *h*^*2*^. The ‘Cluster’ column specifies the meta-trait each trait comprises. As in S16 Table, trait suffixes correspond to the type of stain from which a phenotypic measurement was derived, when present.
(TXT)

S20 Table QTL detected in lung mega-analysis at LOD ≥ 6. For each QTL, the following information is recorded: the meta-trait associated with the locus (‘Trait’), the chromosome (‘Chr’) and position information (‘Pos’, in megabases), the LOD score (‘LOD’), and the bounds of the 2LOD support interval (‘CI_start’ and ‘CI-end’).
(TXT)

S21 Table False discovery rates (FDRs) for each lung meta-trait at LOD ≥ 6 and α = 0.05 significance thresholds. For each meta-trait (‘Trait’), FDR rates are listed at the permutation-based ⍺ = 0.05 (‘alpha_05_FDR’) and LOD ≥ 6 (‘LOD6_FDR’) genome-wide significance thresholds.
(TXT)

S1 Data Pseudomarkers used to interpolate genotype data across study. This is loaded into R as both a genetic map (named ‘gmap’ by default) and a physical map (named ‘pmap’ by default). Each map is a list of 20 vectors corresponding to chromosomes 1–19 and the X chromosome, respectively. Values in each vector correspond to the genetic position in centimorgans (‘gmap’) or physical position in megabases (‘pmap’). The names of each vector correspond to the name of the pseudomarker using the naming convention “chromosome_position”, where position is the position of the pseudomarker in bases.
(RDATA)

S2 Data Genome-wide scan data for aorta meta-traits. (RDATA)

S3 Data Variant association mapping for each aorta meta-trait QTL, and lists of genome annotations within the confidence intervals of the QTL. (ZIP)

S4 Data Genome-wide scan data for lung meta-traits. (RDATA)

S5 Data Lists of genome annotations within lung meta-trait QTL confidence intervals. (ZIP)
