# Supplementary figures and images for: Genetic correlation-guided mega-analysis of DO mice provides mechanistic insight and candidate genes for age-related pathologies
Source: PLoS Genet. 2026 Feb 27;22(2):e1012037. doi: 10.1371/journal.pgen.1012037 (PMC12948109; doi:10.1371/journal.pgen.1012037)

**A**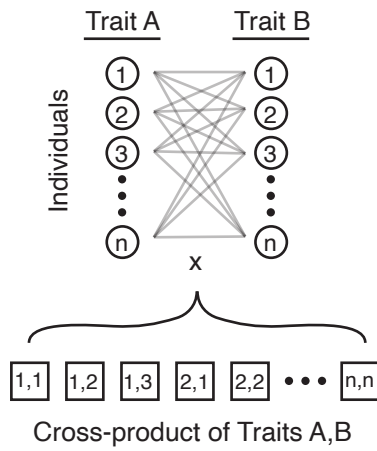**B**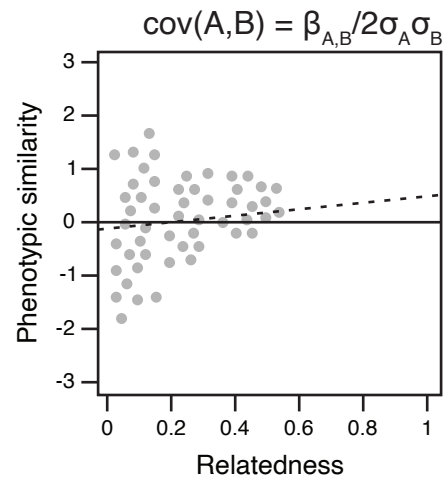**C**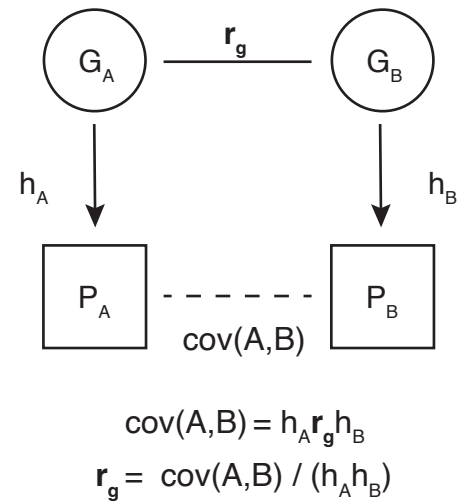**D**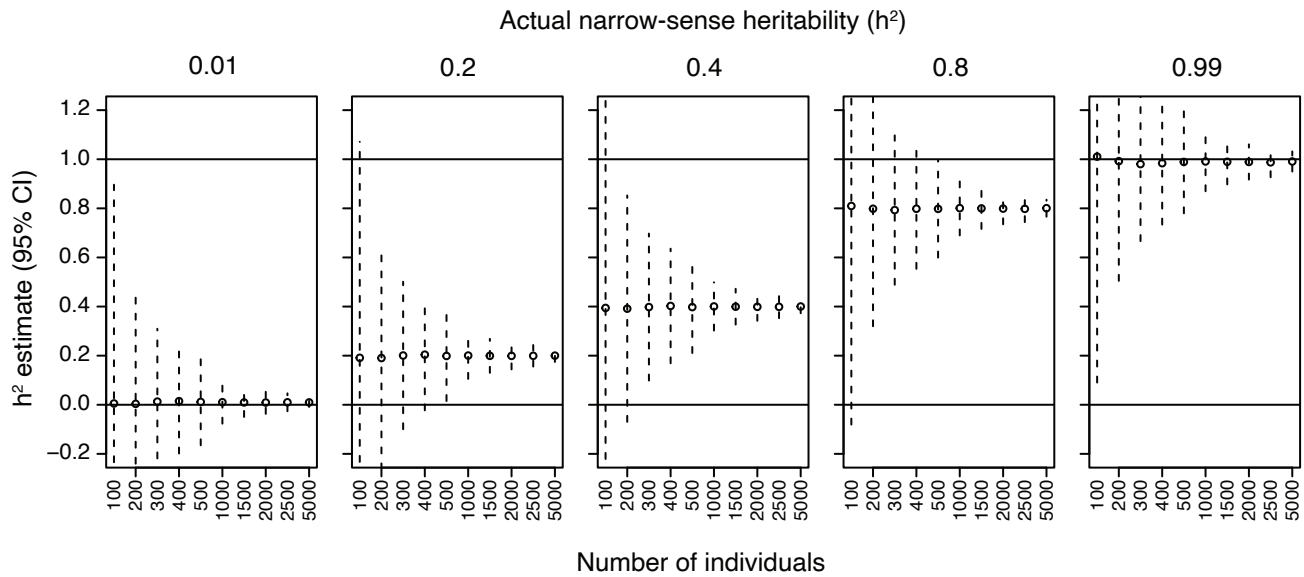

Supplement: S1 Fig — A, Individual phenotype data is collected for two traits (‘A’ and ‘B’). Distributions of traits are z-score normalized and a vectorized outer product is taken by multiplying each measurement of trait A by each measurement of trait B. The result is a set of pairwise phenotypic similarity scores from all combinations of individuals. B, Phenotypic similarity scores for each pair of individuals are regressed on pairwise kinship values. The coefficient of the regression can be used to determine the covariance of traits A and B. C, A structural equation model relates the covariance of traits A and B as a function of relatedness to the genetic correlation (rg) of the two traits. The h2 and covariance of traits A and B must be known in order to solve for rg. D, Haseman-Elston h2 estimates for simulated phenotypes. Mean Haseman-Elston h2 estimates and 95% confidence intervals of 1,000 randomly simulated traits at different sample sizes. Actual h2 of the simulated traits is shown at the top of each panel. (PDF) [file pgen.1012037.s001.pdf]

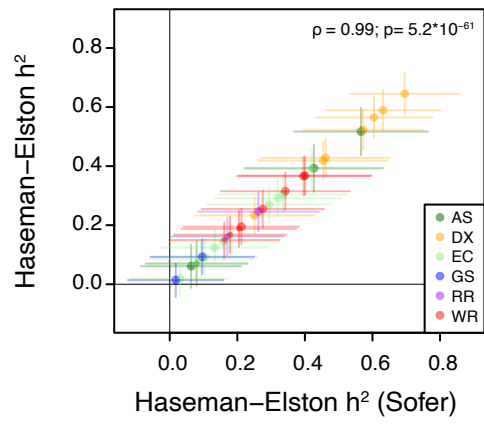

Supplement: S2 Fig — Comparison of h2 estimates and standard errors for 34 DO mouse traits derived from our implementation of Haseman-Elston regression and the implementation of Haseman-Elston regression described in [15]. The Pearson correlation coefficient and accompanying p-value are reported in the upper-right of the plot area.Traits are colored by type: ‘AS’ - Acoustic Startle. ‘DX’ - DEXA scan. ‘EC’ - Echocardiogram. ‘GS’ - Grip Strength. ‘RR’ - Rotarod. ‘WR’ - Wheel Running. (PDF) [file pgen.1012037.s002.pdf]

**A**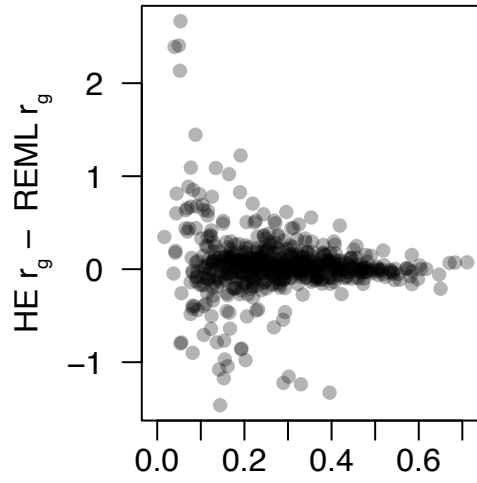**B**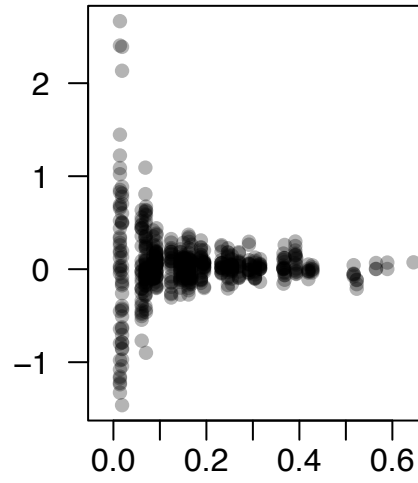**C**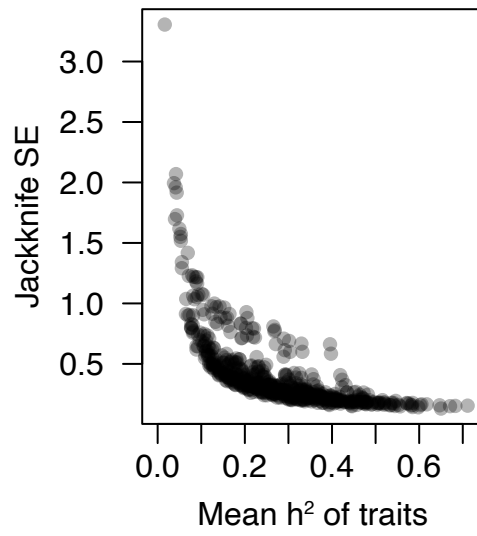**D**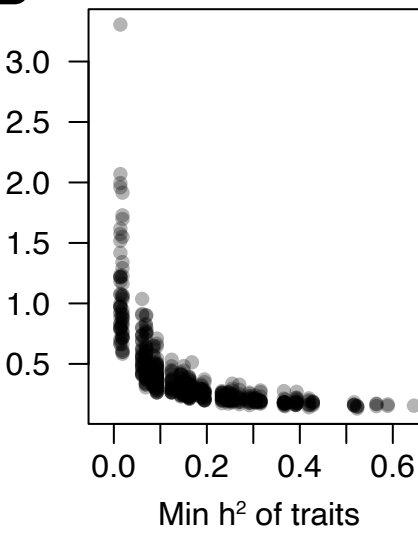

Supplement: S3 Fig — A, Differences in pairwise rg estimates between Haseman-Elston regression and REML as a function of the mean h2 of the traits being compared. B, Differences in pairwise rg estimates between Haseman-Elston regression and REML as a function of the minimum h2 of the traits being compared. C, Standard error of Haseman-Elston based rg estimates as a function of the mean h2 of the traits being compared. D, Standard error of Haseman-Elston based rg estimates as a function of the minimum h2 of the traits being compared. (PDF) [file pgen.1012037.s003.pdf]

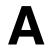

Supplement: S4 Fig — Mean rpg estimates among traits from each study. Values derived from a single trait pair are represented by gray boxes. ‘Calico internal’ traits comprise open field, frailty, lung, and aorta traits, including histology-based phenotypes. (PDF) [file pgen.1012037.s004.pdf]

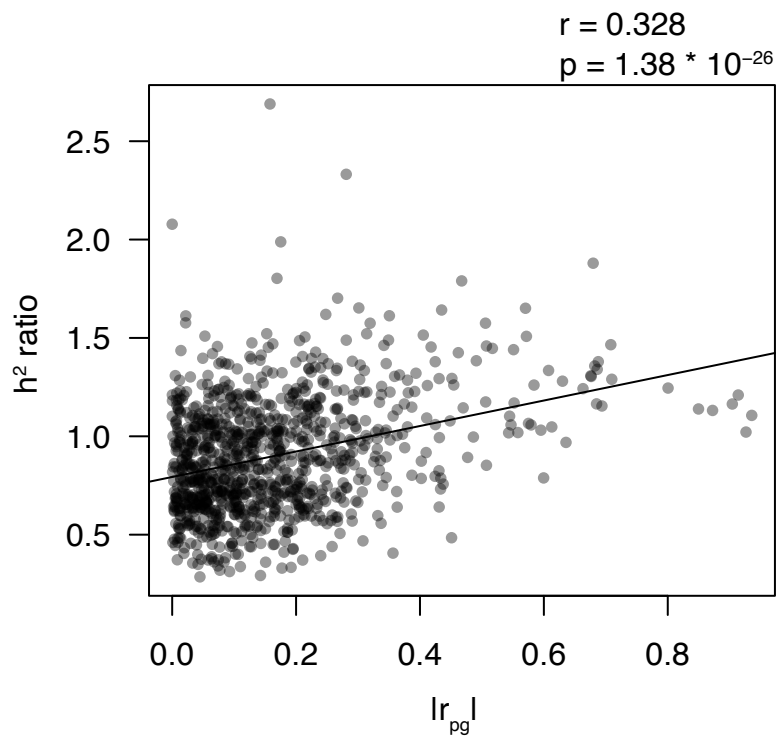

Supplement: S5 Fig — Scatterplot of the h2 ratio for 1,000 random pairs of traits as a function of the absolute value of their rpg. The h2 ratio for traits A and B is defined as the h2meta/ mean(h2A,h2B), where h2meta is the heritability of a meta-trait constituting traits A and B, and h2A and h2B are the individual heritabilities of traits A and B. The Pearson correlation coefficient and p-value between the h2 ratio and |rpg| are reported. (PDF) [file pgen.1012037.s005.pdf]

Figure S6, Mullis et al, 2025

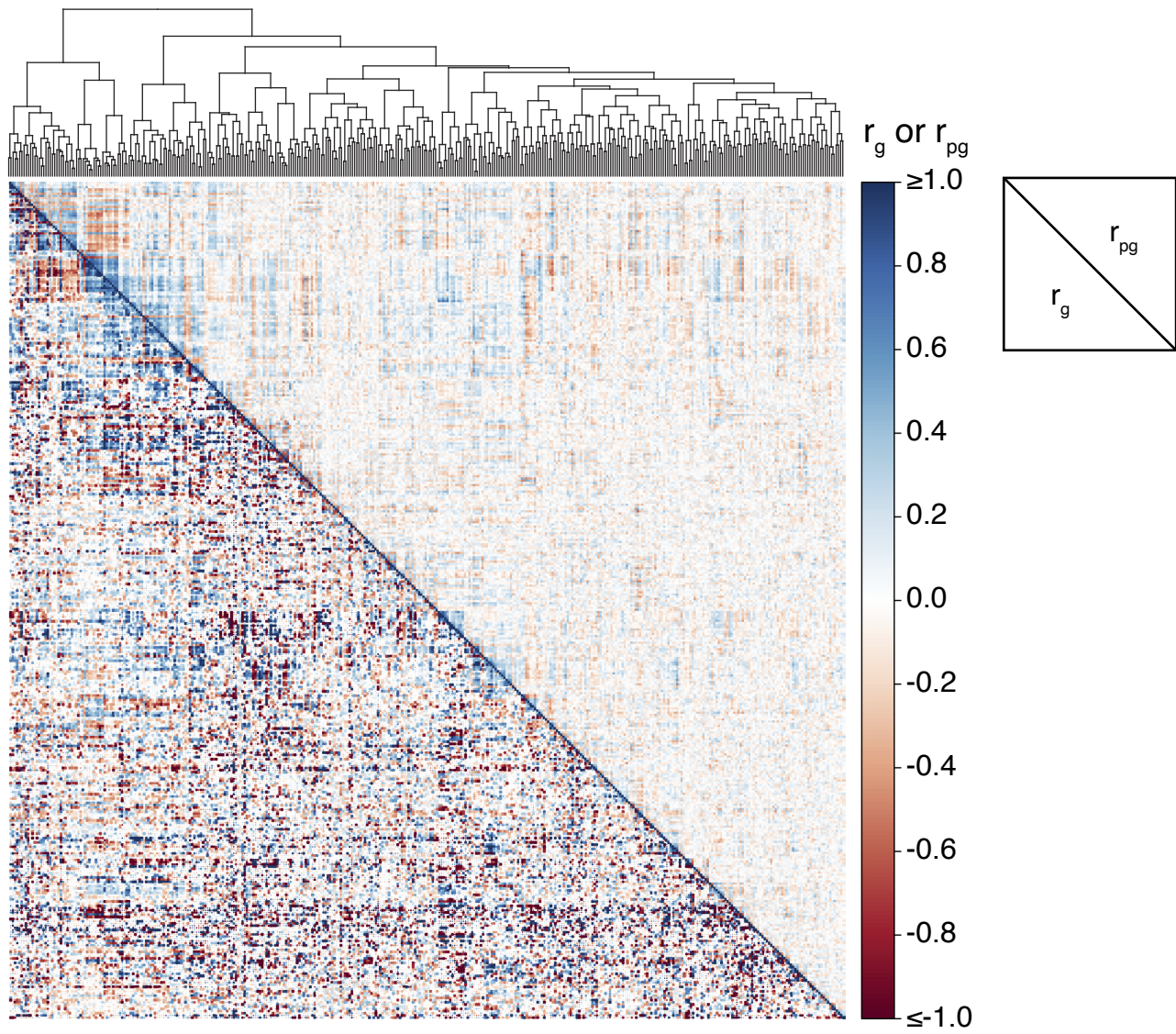

Supplement: S6 Fig — Hierarchically clustered heatmap of rg (lower triangle) and rpg (upper triangle) estimates among 383 DO meta-traits. The accompanying dendrogram shows euclidean distances among meta-traits based on their rpg estimates. (PDF) [file pgen.1012037.s006.pdf]

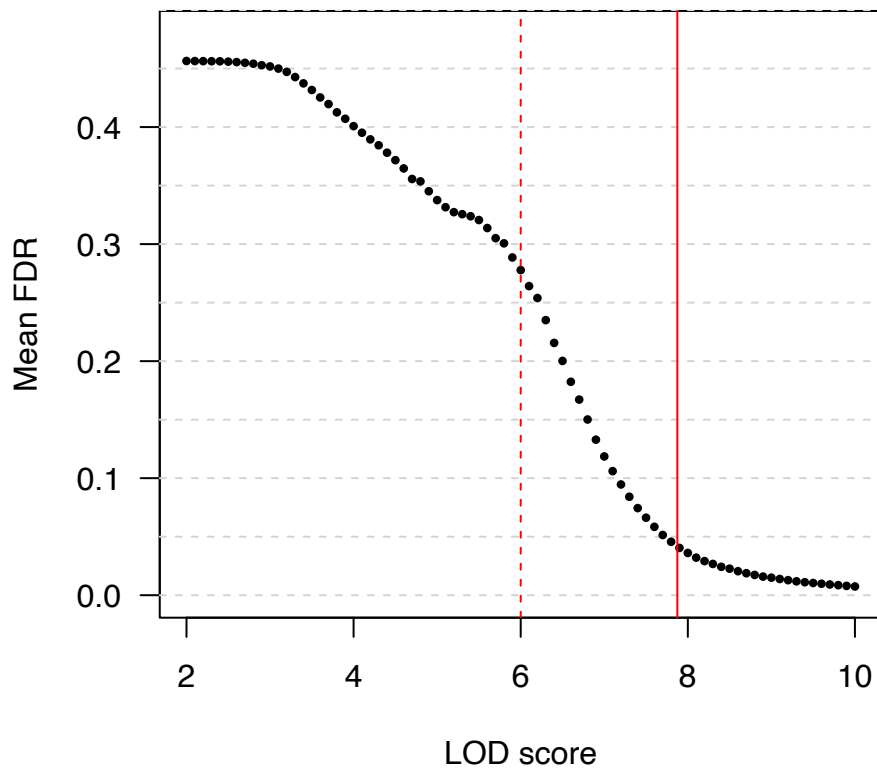

Supplement: S7 Fig — The solid vertical line corresponds to the average genome-wide significance threshold of ⍺ = 0.05 based on 1,000 permutations of the data, performed separately on each meta-trait. The dashed vertical line corresponds to the LOD-based threshold of 6. (PDF) [file pgen.1012037.s007.pdf]

**A**

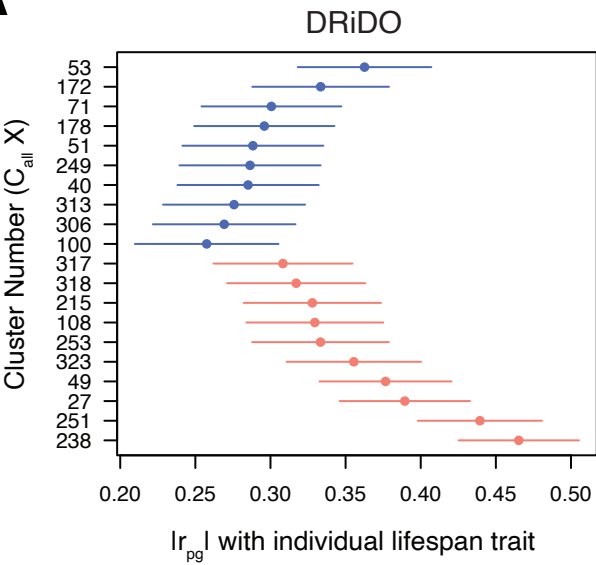

**B**

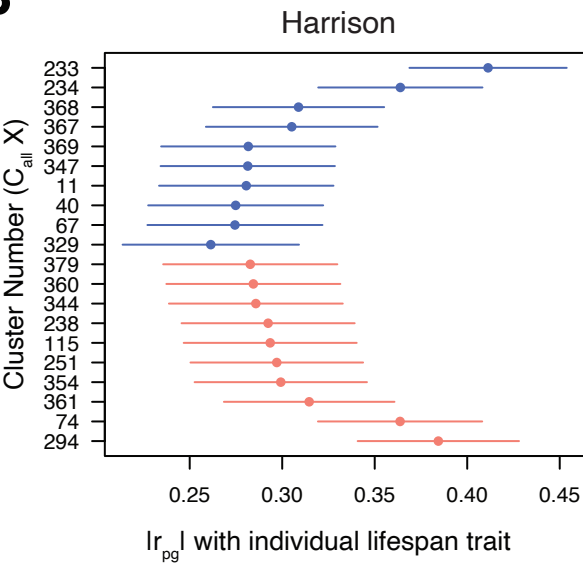

**C**

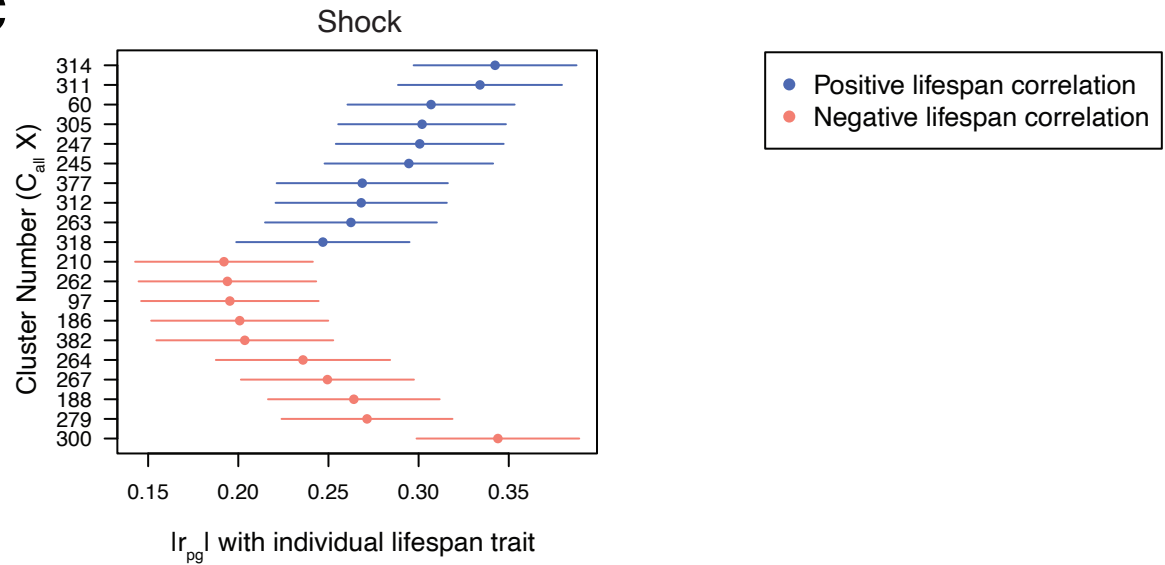

Supplement: S8 Fig — The strongest positive and negative rpg estimates between meta-traits and lifespan data measured in A, the DRiDO study, B, the Harrison study, and C, the Shock study. Error bars correspond to the standard error of each rpg estimate. Meta-trait number (cluster number) is shown on the left hand side of the plot. The traits comprising each cluster can be found in S6 Table. (PDF) [file pgen.1012037.s008.pdf]

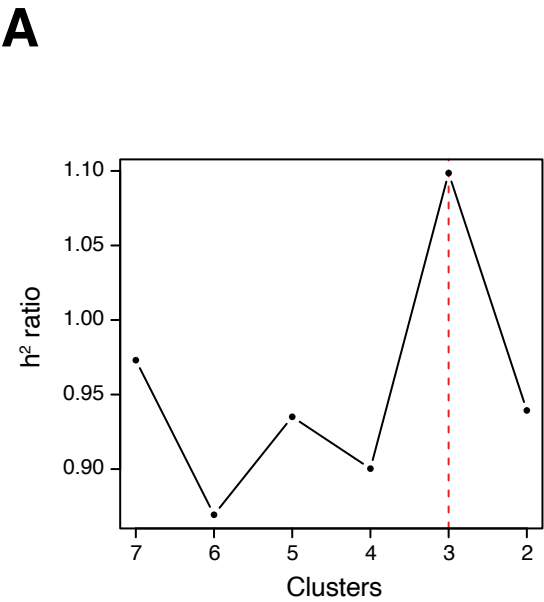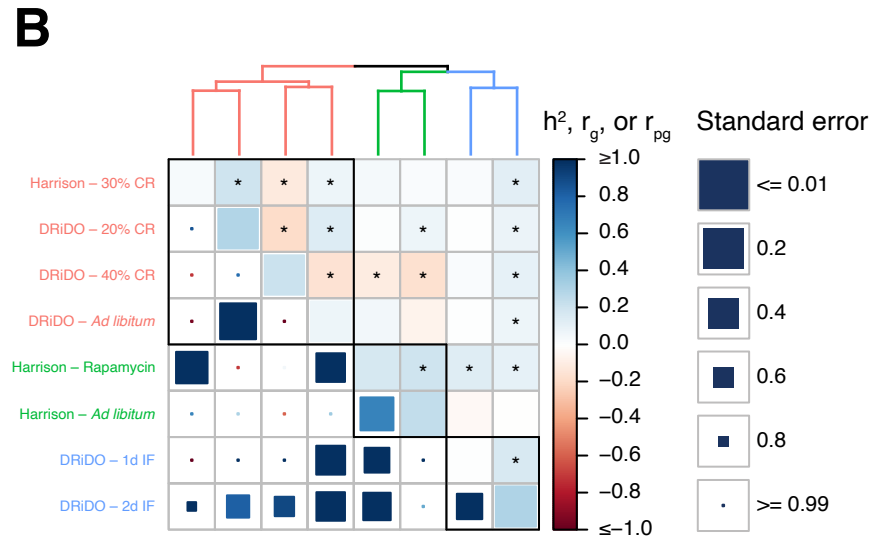

Supplement: S9 Fig — A, Plot of mean h2 ratios among clusters of diet- or drug-specific lifespan measurements at different levels of hierarchical clustering (k). B, Hierarchically clustered heatmap of rg (lower triangle) and rpg (upper triangle) among lifespan measurements within individual dietary or drug intervention groups. Statistically significant estimates after correcting for phenome-wide multiple testing are denoted by an asterisk. (PDF) [file pgen.1012037.s009.pdf]

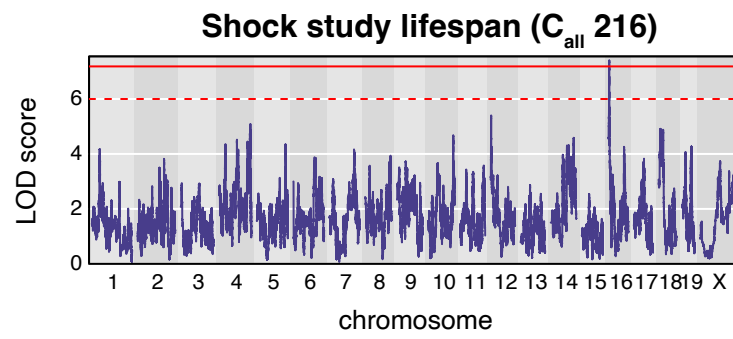

Supplement: S10 Fig — Solid red lines indicate a trait-specific and permutation-based genome-wide significance threshold. Dashed red lines indicate a nominal significance threshold of LOD > 6. (PDF) [file pgen.1012037.s010.pdf]

**A**

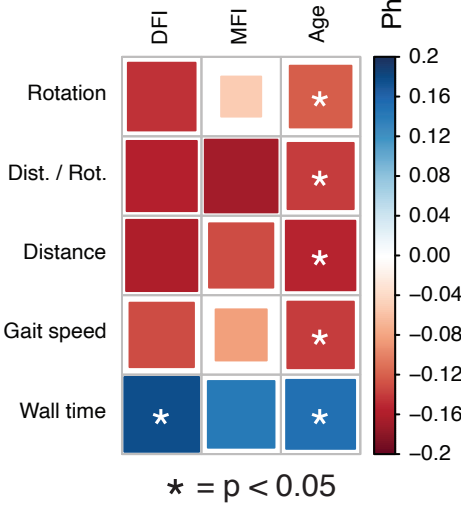

**B**

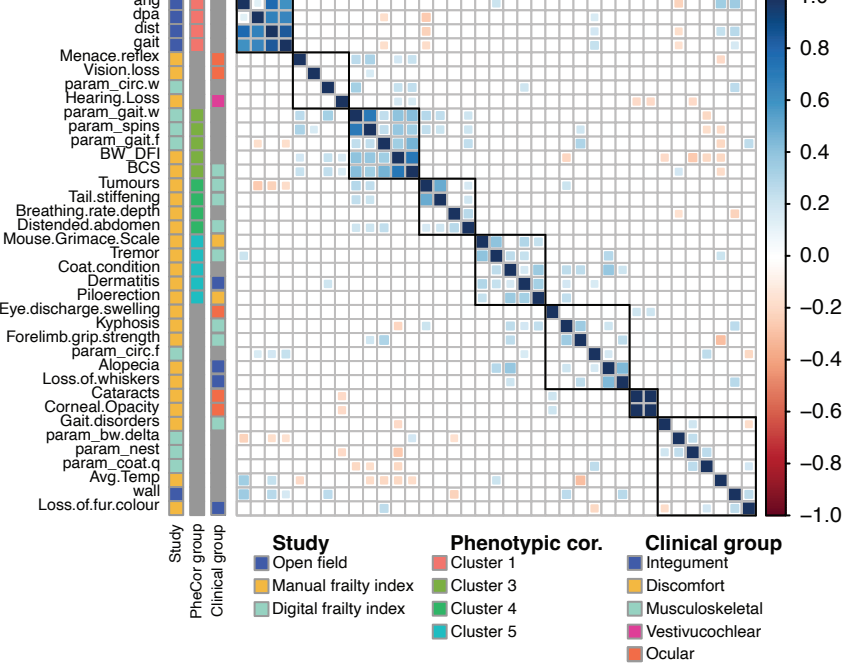

**C**

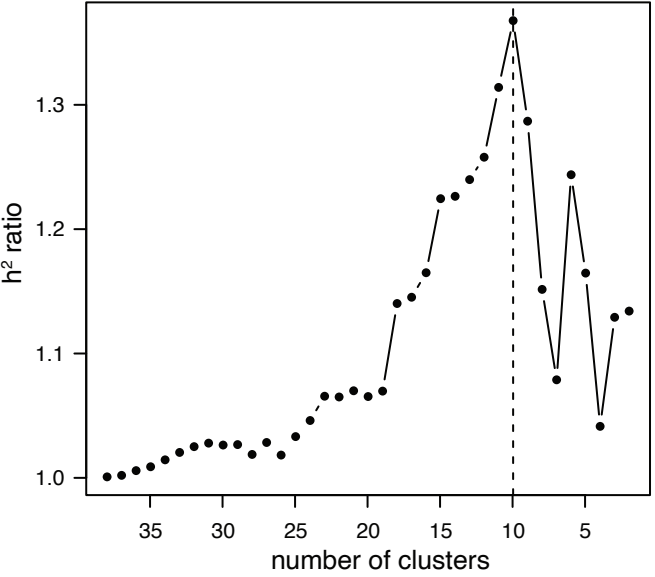

Supplement: S11 Fig — A, Phenotypic correlations between open field traits and mean digital frailty index scores (DFI), mean manual frailty index scores (MFI), and age. Statistically significant correlations are denoted by an asterisk. B, Phenotypic correlations among open field, DFI, and MFI traits. C. (PDF) [file pgen.1012037.s011.pdf]

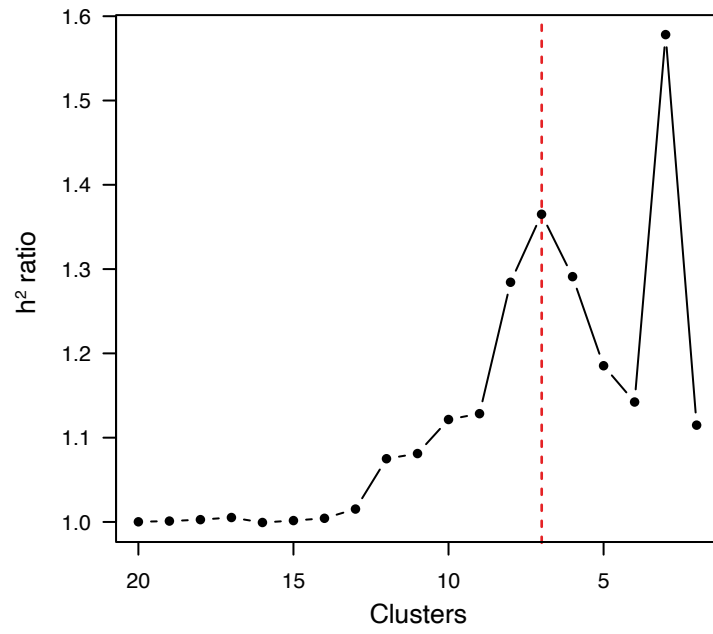

Supplement: S13 Fig — Plot of mean h2 ratios among clusters of aorta meta-traits at different levels of hierarchical clustering (k). (PDF) [file pgen.1012037.s013.pdf]

**A**

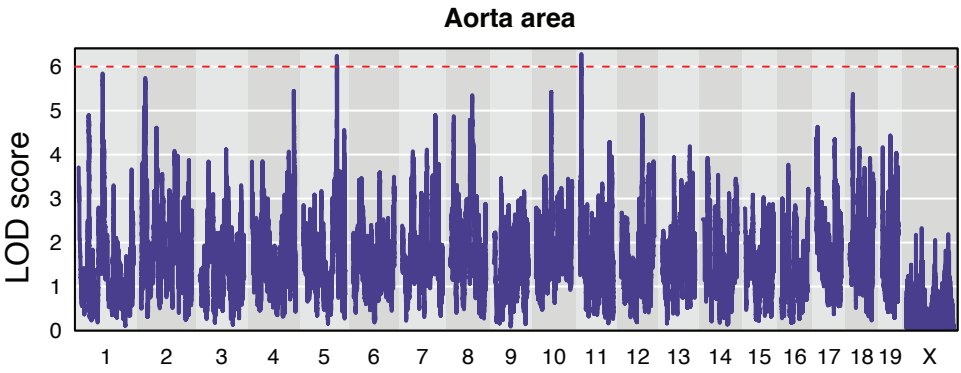

**B**

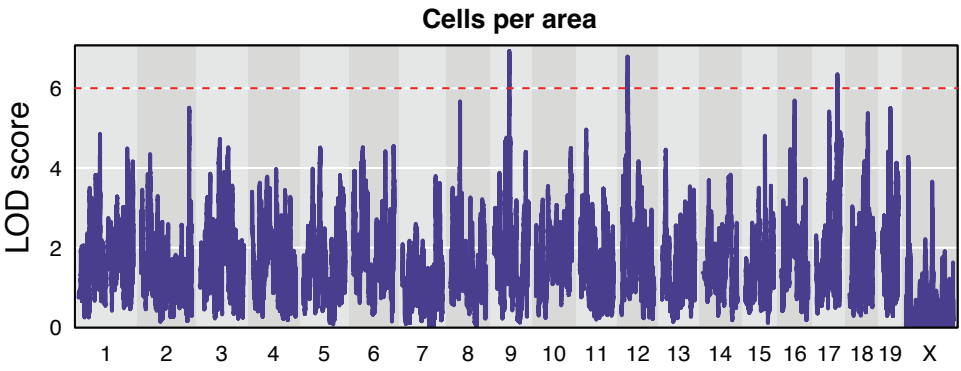

**C**

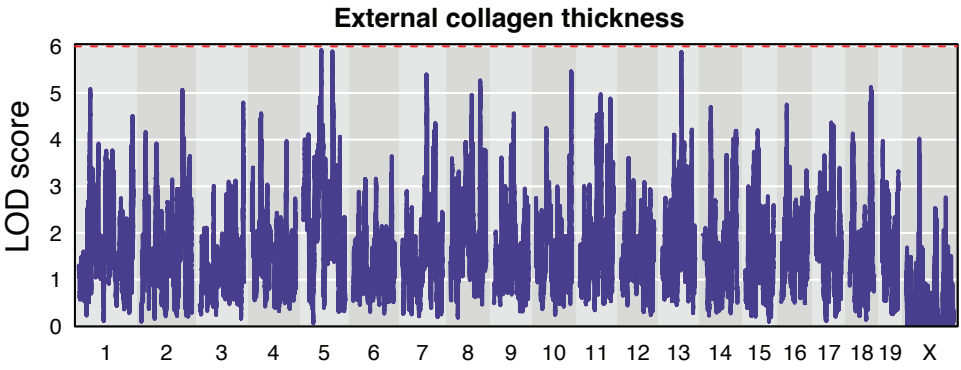

**D**

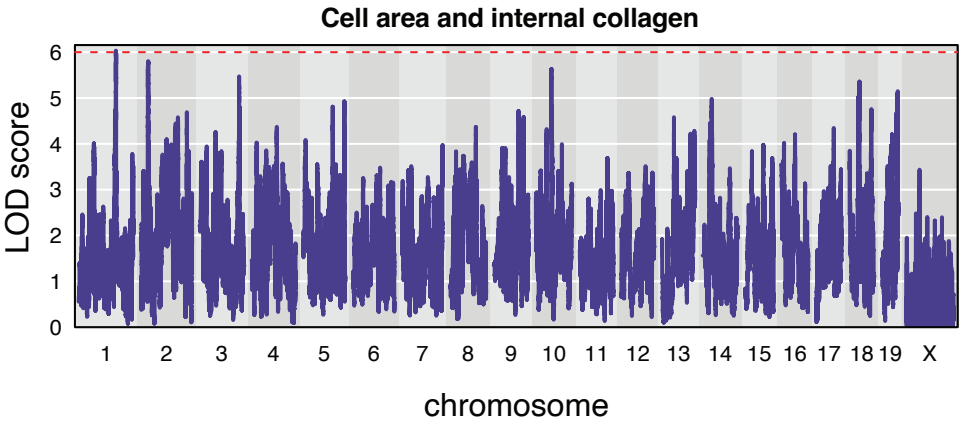

Supplement: S14 Fig — Manhattan plots of additive whole-genome scans on aggregated aorta phenotypes: A, Aorta area. B, Cells per area. C, External collagen thickness. D, Cell area and internal collagen. Solid red lines indicate a trait-specific and permutation-based genome-wide significance threshold. Dashed red lines indicate a nominal significance threshold of LOD > 6. (PDF) [file pgen.1012037.s014.pdf]

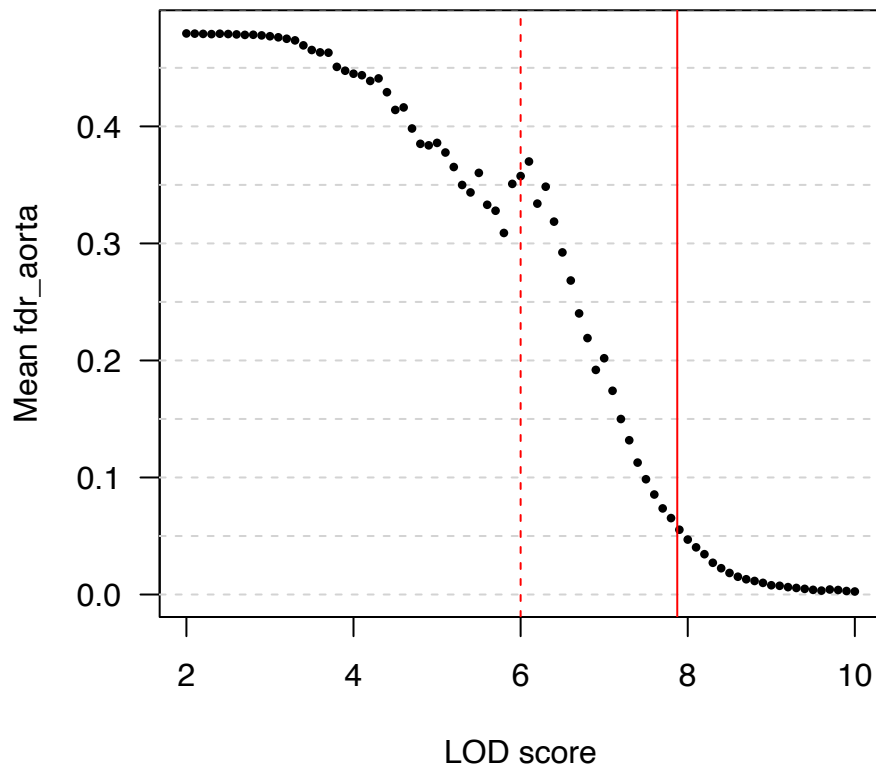

Supplement: S15 Fig — The solid vertical line corresponds to the average genome-wide significance threshold of ⍺ = 0.05 based on 1,000 permutations of the data, performed separately on each meta-trait. The dashed vertical line corresponds to the LOD-based threshold of 6. (PDF) [file pgen.1012037.s015.pdf]

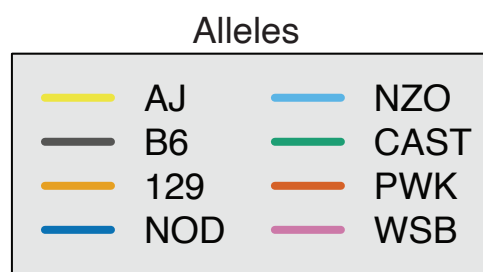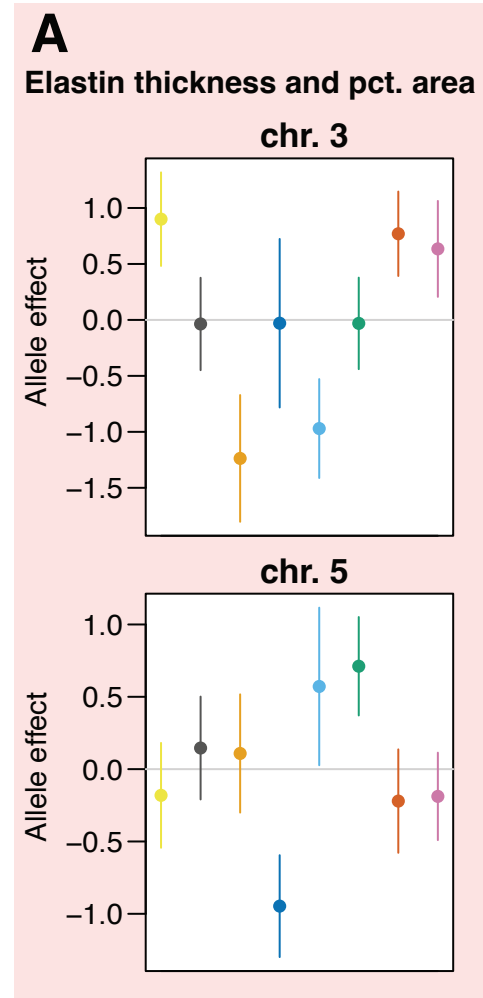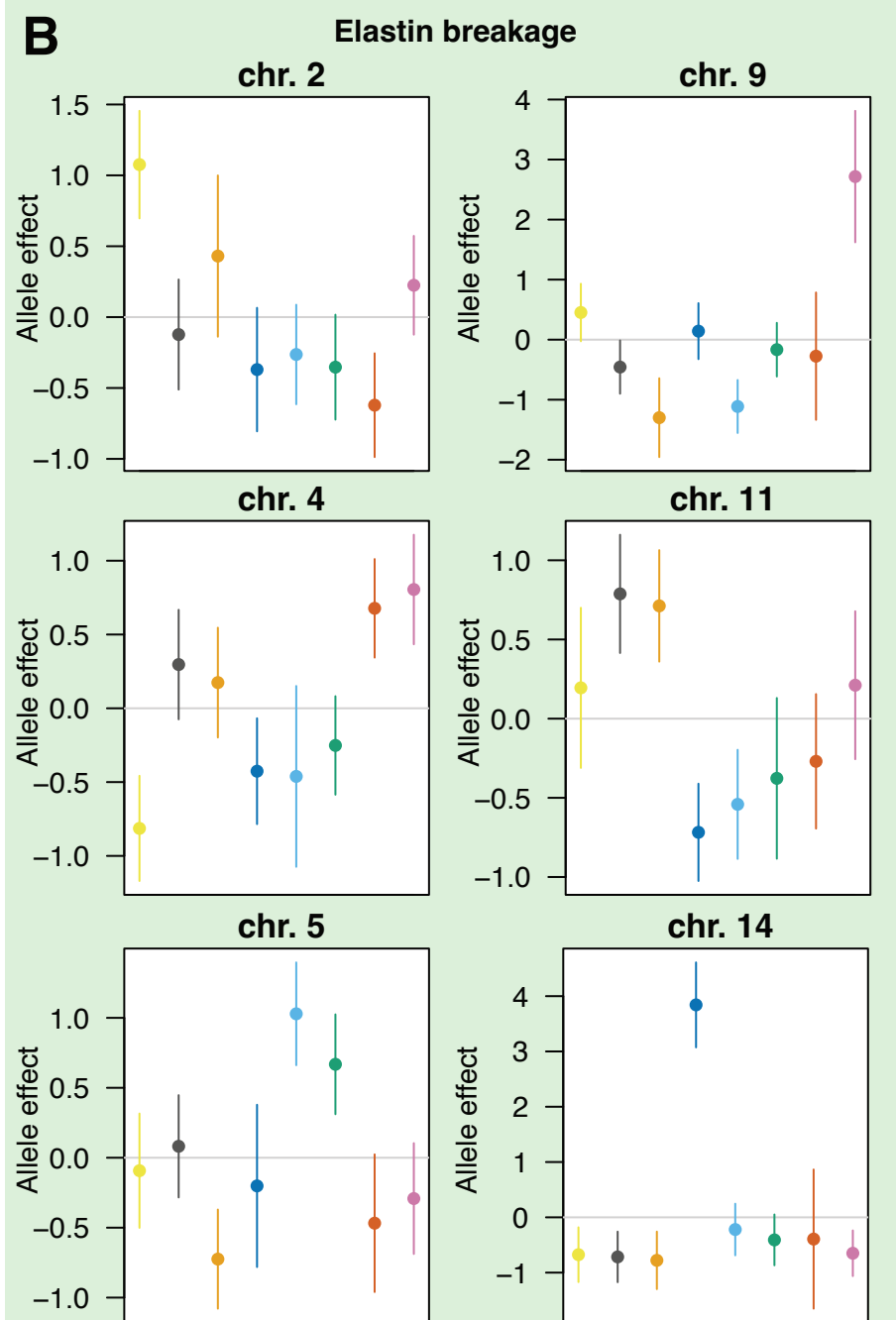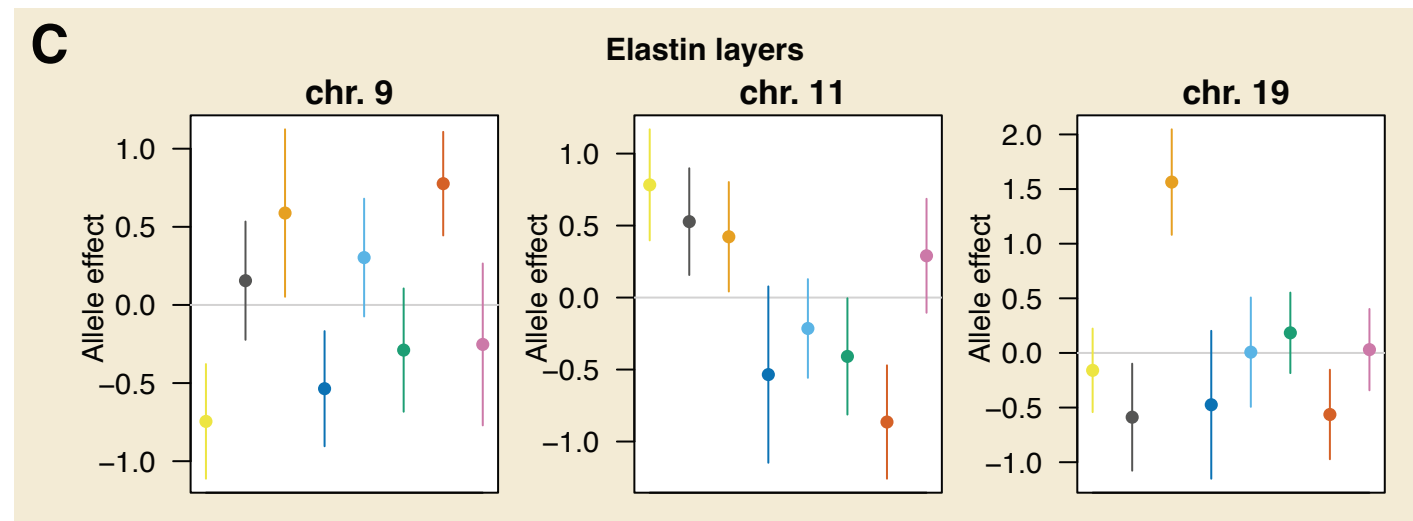

Supplement: S16 Fig — Allelic effects (BLUPs) and corresponding standard errors of the loci detected in genome-wide scans of elastin meta-traits. A, Elastin thickness and percent area. B, Elastin breakage and tortuosity. C, Number of elastin layers. (PDF) [file pgen.1012037.s016.pdf]

Figure S17, Mullis et al, 2025

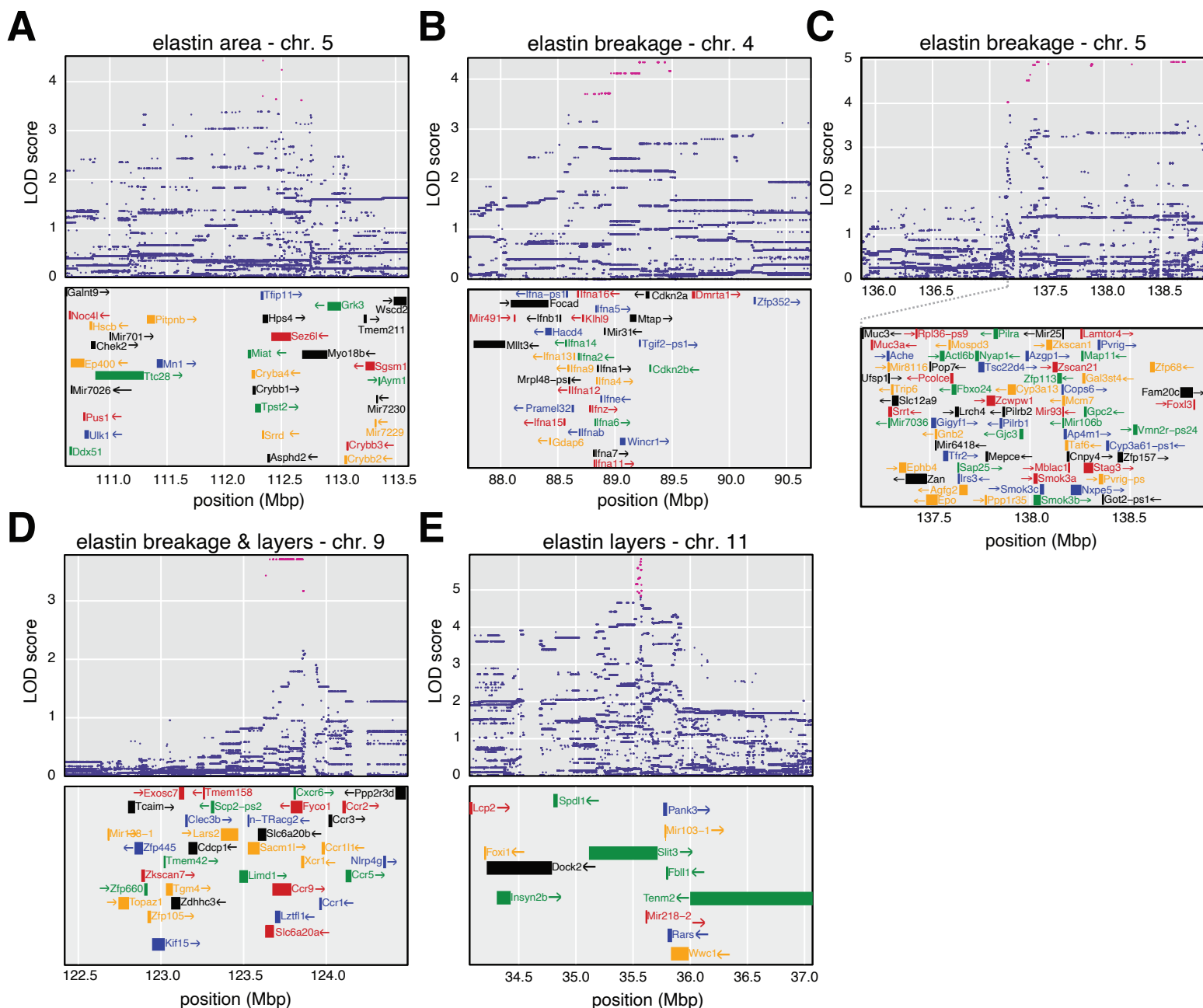

Supplement: S17 Fig — A-E, Variant association mapping plots for each elastin QTL at which the allelic effects were significantly correlated with at least one cis-eQTL in an external transcriptomic dataset from cardiac tissue in aging mice. (Top) The LOD scores for each variant within the 2 LOD support intervals around each locus in the meta-analysis. The most likely candidate SNPs are highlighted in pink. (Bottom) Genes within the 2 LOD support intervals are depicted, with the exception of panel C, which depicts the genes underlying the most likely SNPs. (PDF) [file pgen.1012037.s017.pdf]

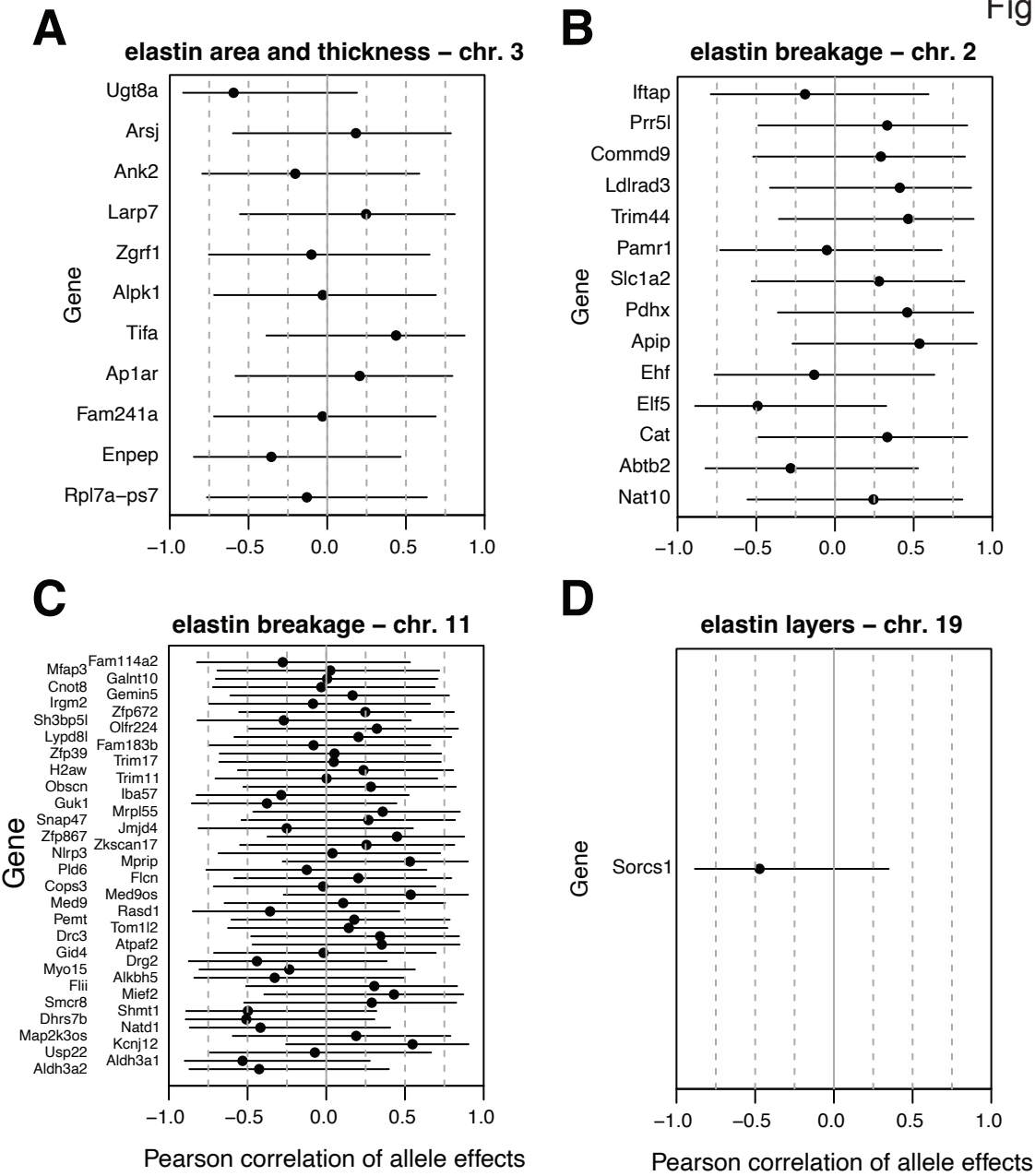

Supplement: S18 Fig — A-D, Correlations of haplotype effects between the peak markers at QTL influencing elastin phenotypes and cis-eQTL in an external DO cardiac tissue transcriptomic dataset. Candidate genes are highlighted with red text. (PDF) [file pgen.1012037.s018.pdf]

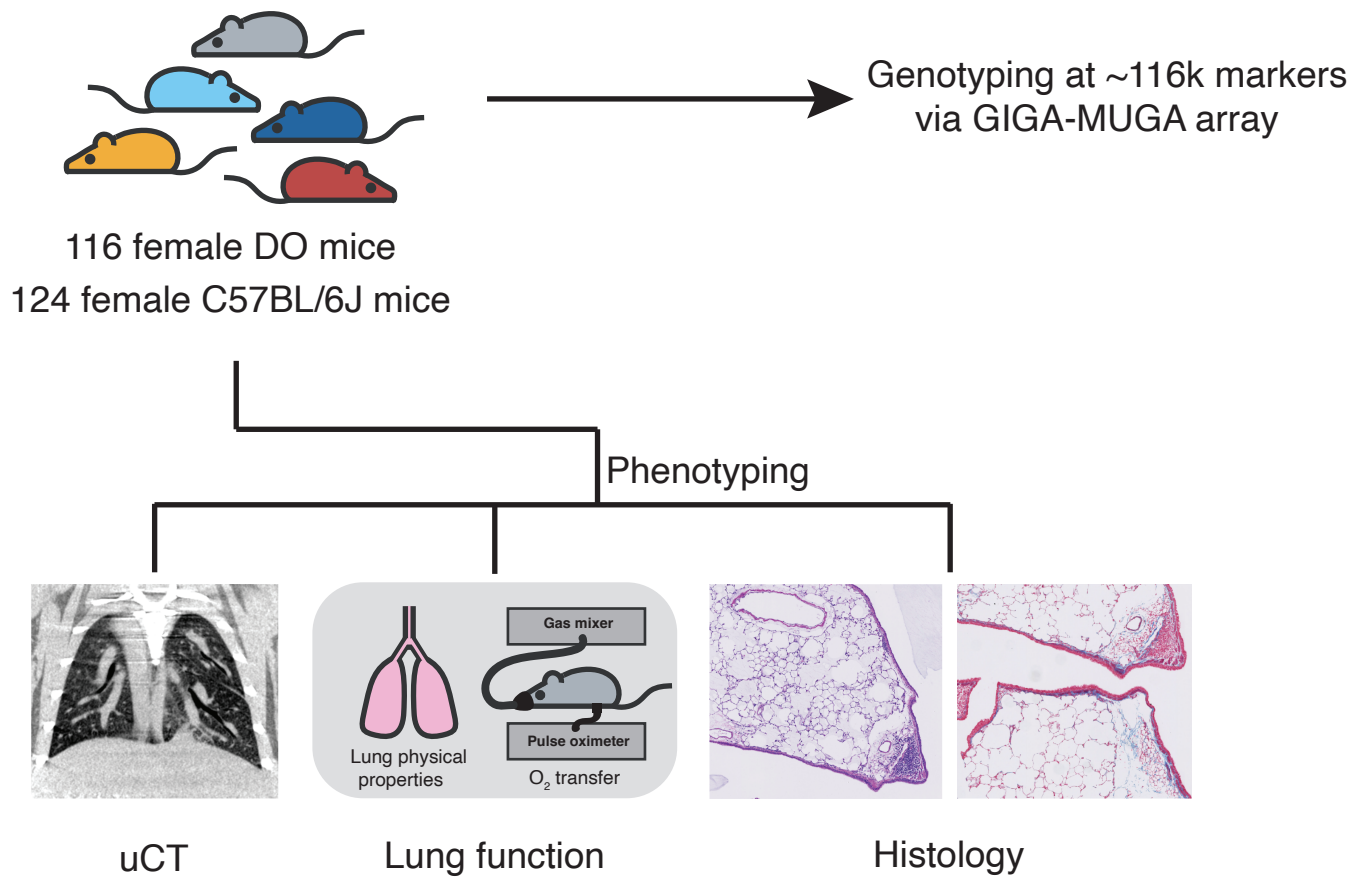

Supplement: S19 Fig — Study design for the assessment of lung properties in mice. 116 DO mice (and 124 C57BL/6J) animals were genotyped and participated in a phenotyping pipeline that included µCT scans of the lungs and functional measurements. Upon exit from the study, lung tissue was harvested and fixed, and tissue sections were stained (TC or HE) and imaged. (PDF) [file pgen.1012037.s019.pdf]

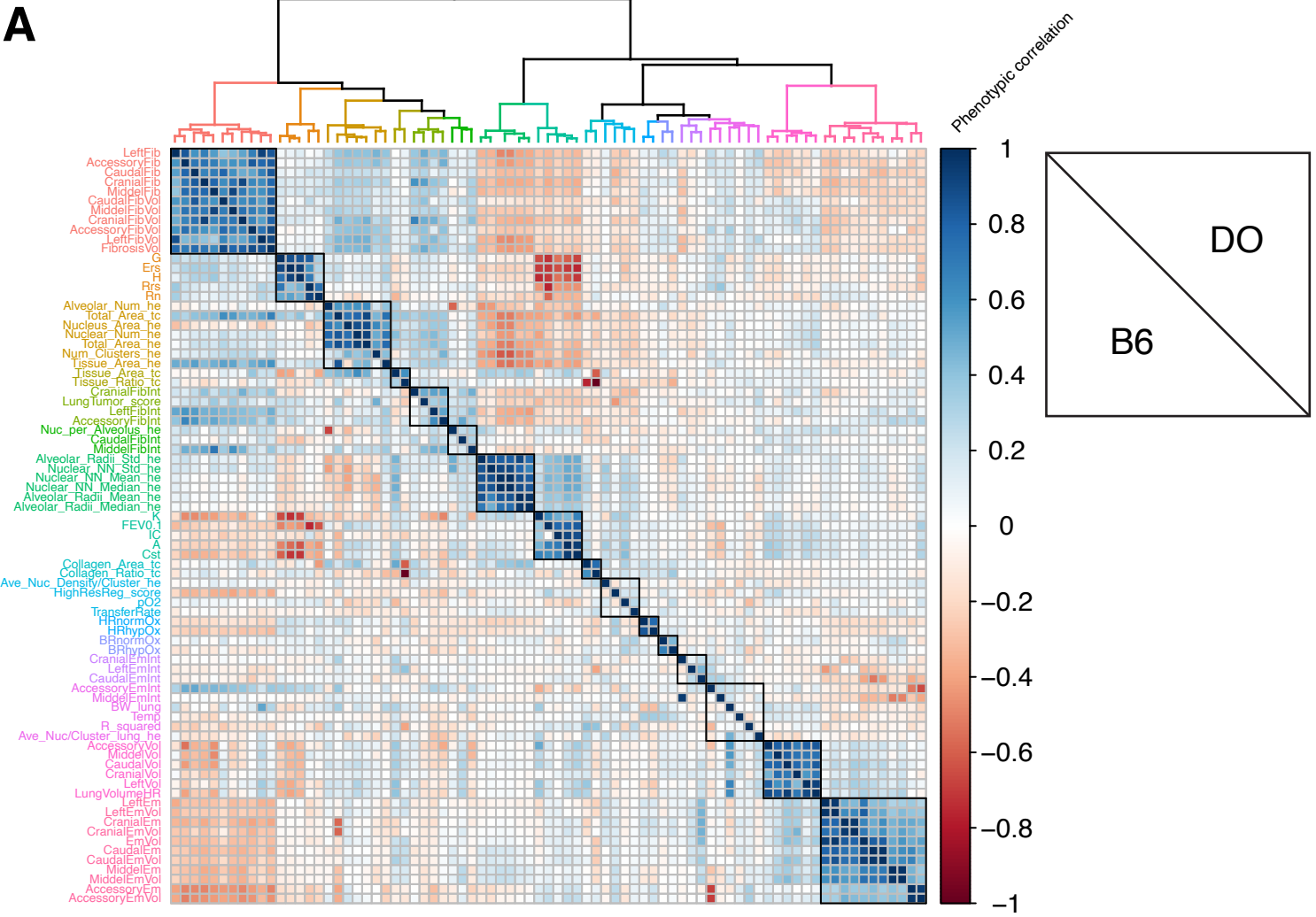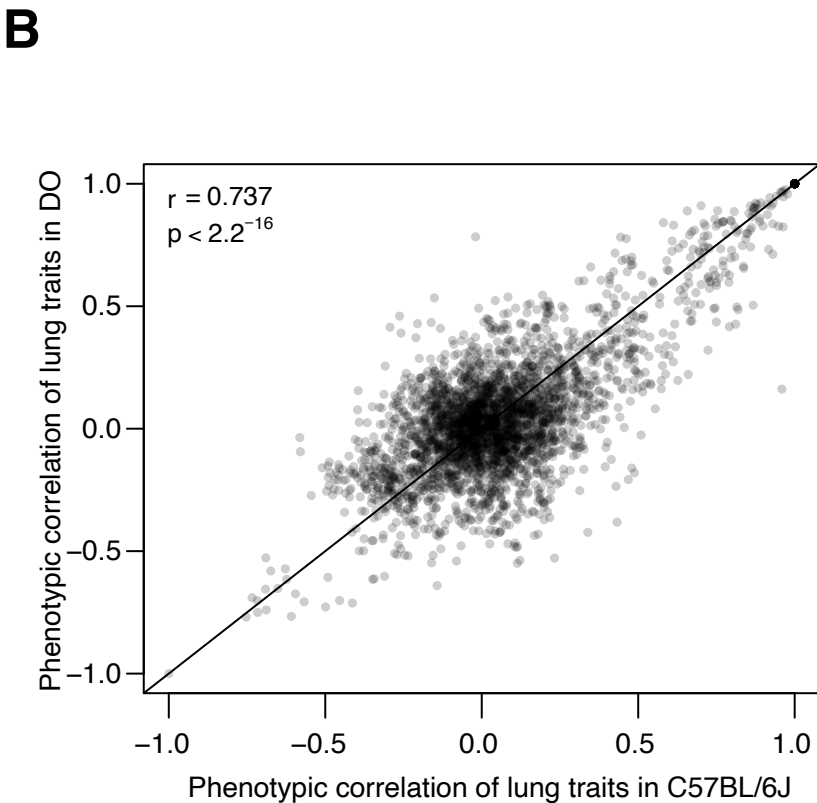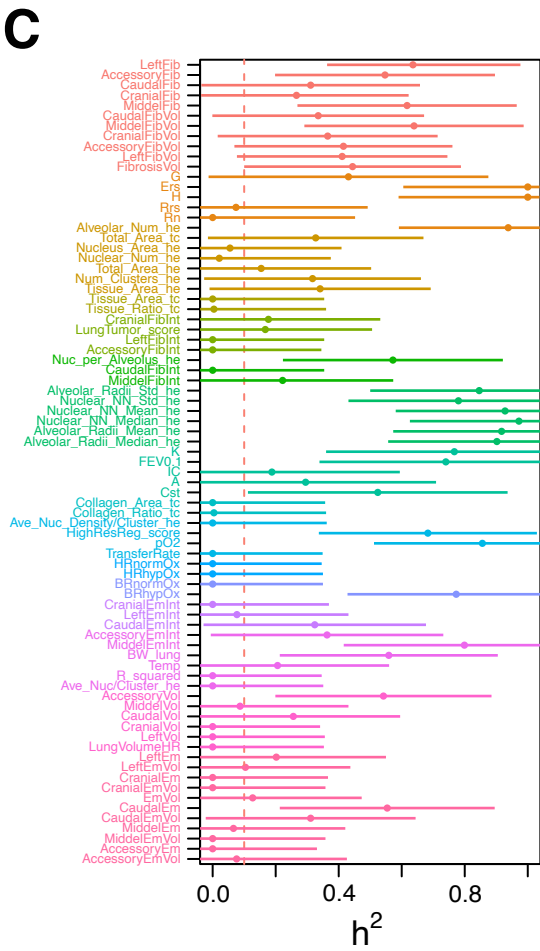

Supplement: S20 Fig — A, Phenotypic correlations among hierarchically clustered lung phenotypes in DO (upper triangle) and C57BL/6J (lower triangle) mice. Clusters were determined via silhouette score; phenotype names and the accompanying dendrogram are colored by cluster. B, Pairwise phenotypic correlations among pairs of lung traits plotted as a function of their correlation in C57BL/6J mice. C, Heritability estimates and standard errors of each lung phenotype prior to filtering. The dashed red line indicates the threshold of h2 < 0.1 at which traits were excluded from downstream analysis. (PDF) [file pgen.1012037.s020.pdf]

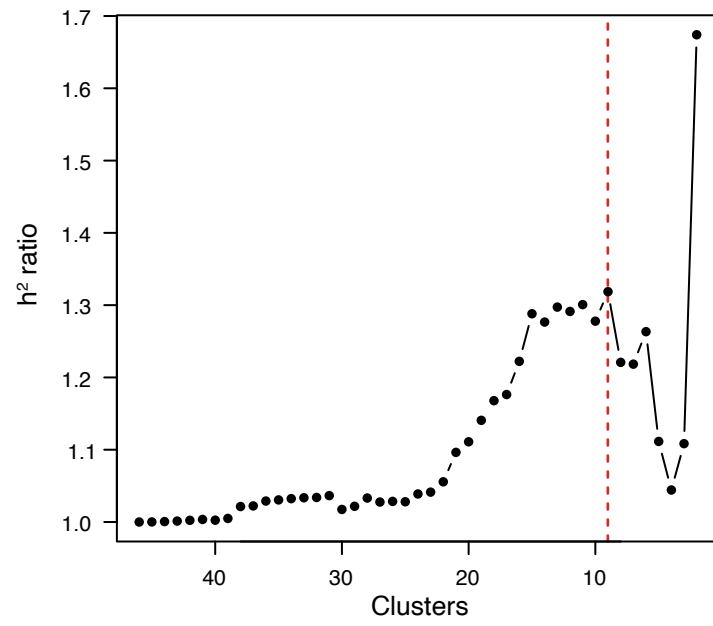

Supplement: S21 Fig — Plot of mean h2 ratios among clusters of lung meta-traits at different levels of hierarchical clustering (k). (PDF) [file pgen.1012037.s021.pdf]

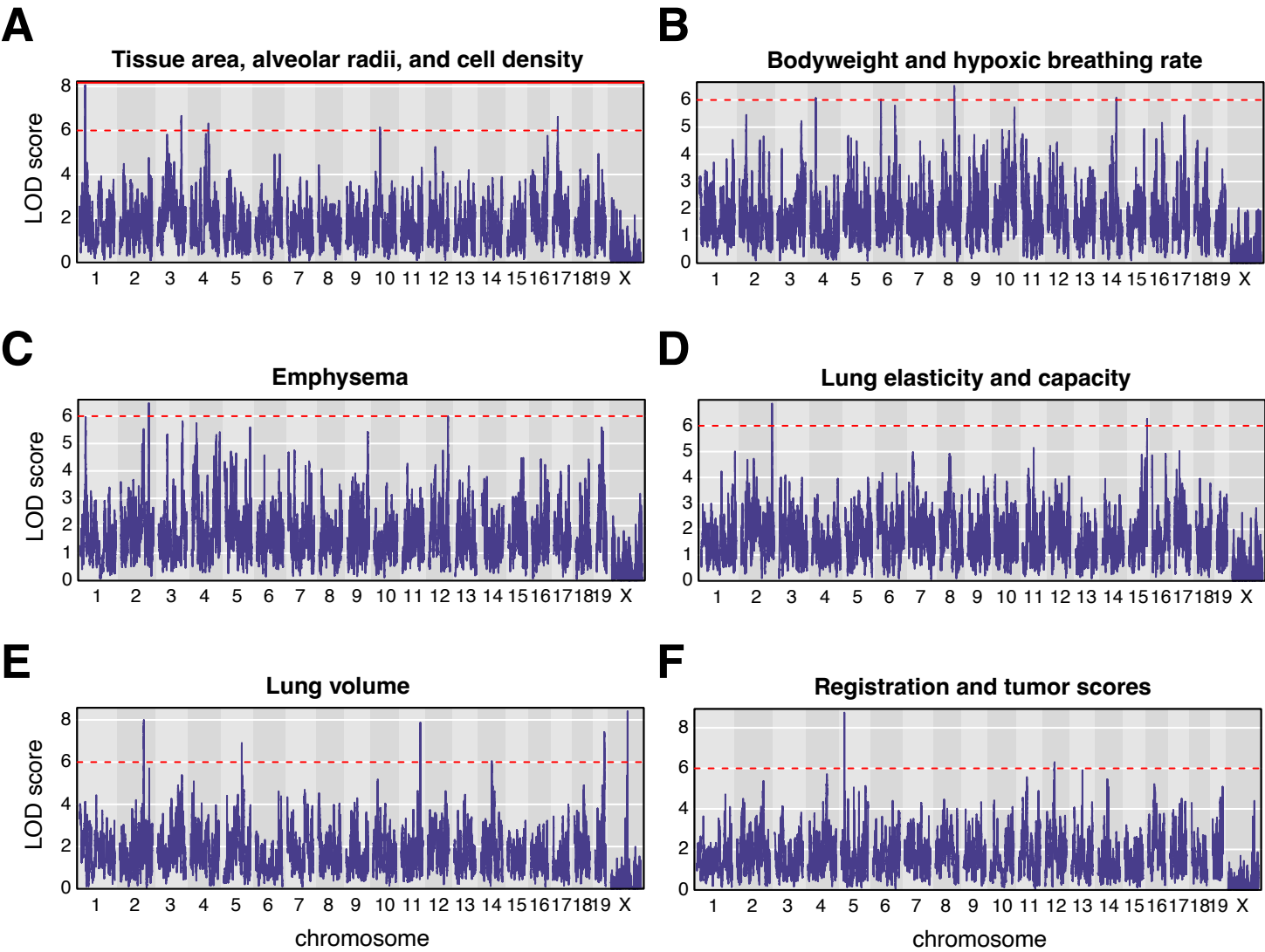

Supplement: S22 Fig — Manhattan plots of additive genome-wide scans for additional meta-traits composed of clustered lung phenotypes: A, Tissue area, alveolar radii, and cell density. B, Body weight and breathing rate under hypoxic conditions. C, Emphysema as measured via µCT imaging. D, Lung elasticity and capacity. E, Lung volume. F, Mouse registration and lung tumor scores. Solid red lines indicate a trait-specific and permutation-based genome-wide significance threshold. Dashed red lines indicate an additional threshold of LOD > 6. (PDF) [file pgen.1012037.s022.pdf]

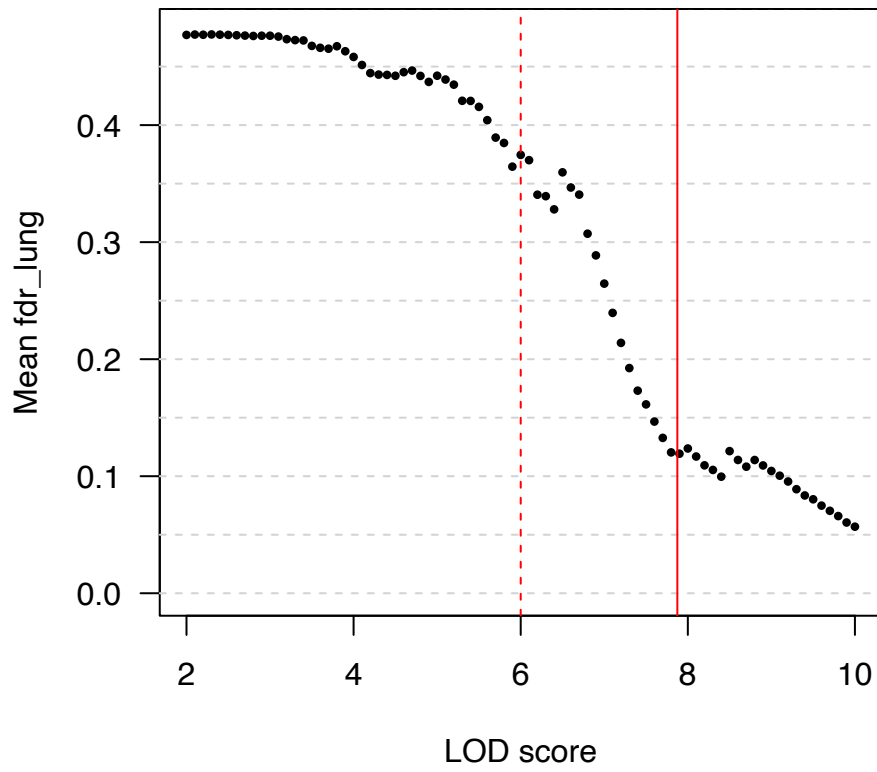

Supplement: S23 Fig — The solid vertical line corresponds to the average genome-wide significance threshold of ⍺ = 0.05 based on 1,000 permutations of the data, performed separately on each meta-trait. The dashed vertical line corresponds to the LOD-based threshold of 6. (PDF) [file pgen.1012037.s023.pdf]

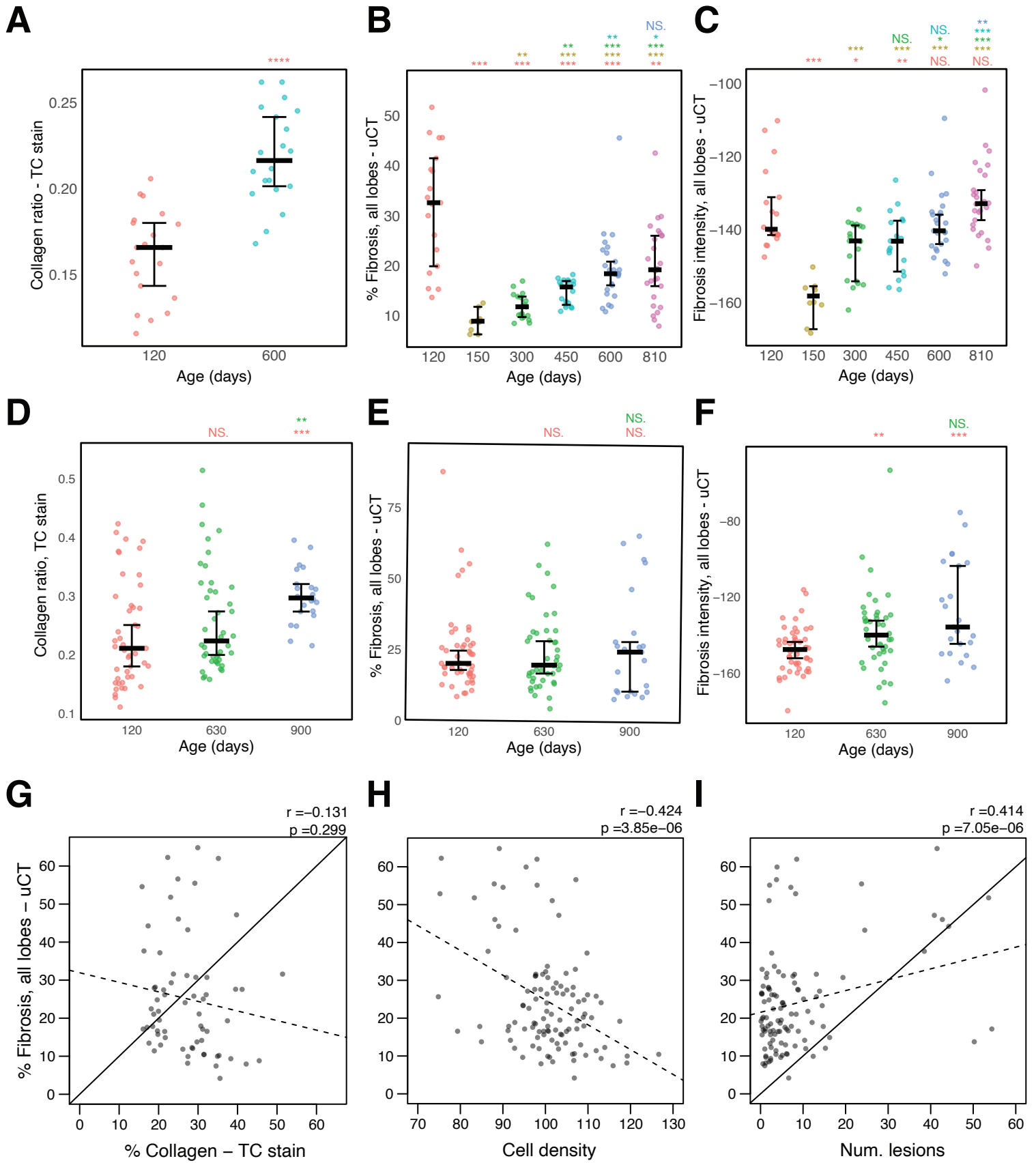

Supplement: S24 Fig — A, Fraction of lung tissue area stained for collagen via trichrome (TC) staining as a function of age in C57BL/6J mice. B, Percent area of the lung categorized as “high density” via µCT scan in C57BL/6J mice. C, Pixel intensity of “high density” tissue in µCT scans in C57BL/6J mice. D, Fraction of lung tissue area stained for collagen via TC staining as a function of age in DO mice. E, Percent area of the lung categorized as “high density” via µCT scan in DO mice. F, Pixel intensity of “high density” tissue in µCT scans in DO mice. G-H, Percent area of the lung categorized as “high density” via µCT scan as a function of (G) percent tissue area stained for collagen via TC stain, (H) the cellular density in the tissue via H&E (HE) staining, and (I) the number of abnormal lesions in the tissue in DO mice. The Pearson correlation coefficients and corresponding p values are reported above each plot. The solid lines are lines of identity and the dashed lines are best-fine lines from linear models. (PDF) [file pgen.1012037.s024.pdf]

Figure S25, Mullis et al, 2025

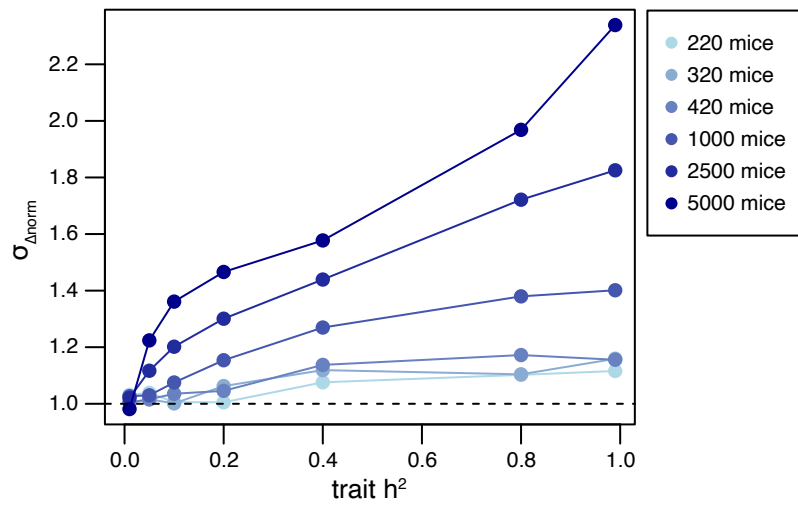

Supplement: S25 Fig — Standard deviation of σ∆norm values for simulated phenotypes across a range of h2 and population sizes. Values less than one indicate that the standard error of h2 for the simulated phenotypes is greater than expected. Values greater than one indicate that the precision of the standard error of h2 estimates is less than expected. (PDF) [file pgen.1012037.s025.pdf]

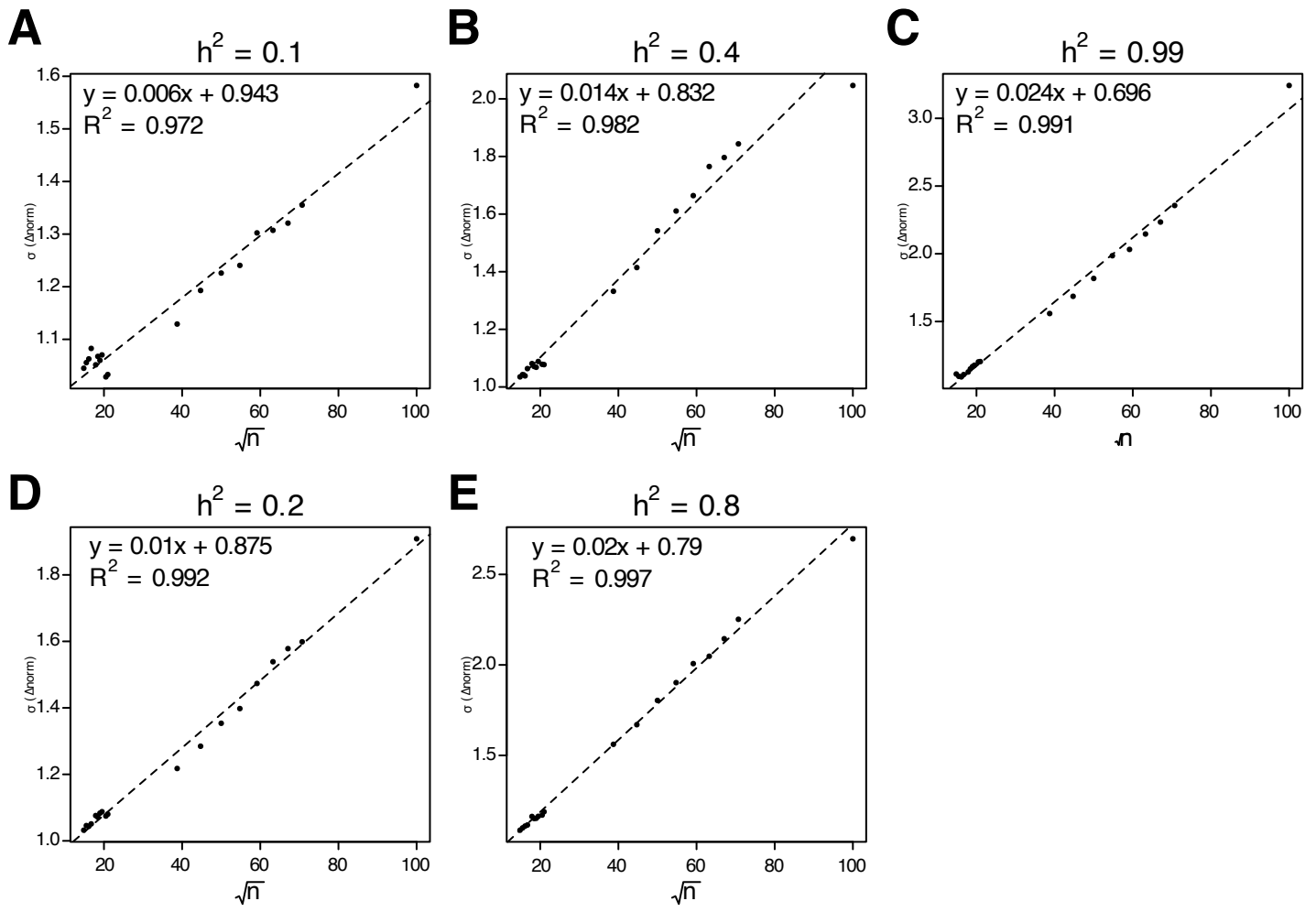

Supplement: S26 Fig — Linear regressions of σ∆norm values for simulated phenotypes across different population sizes (n) at A, h2 = 0.1, B, h2 = 0.2, C, h2 = 0.4, D, h2 = 0.8, E, h2 = 0.99. The coefficients and R2 of each linear regression are reported in the upper left of each panel. (PDF) [file pgen.1012037.s026.pdf]

**A**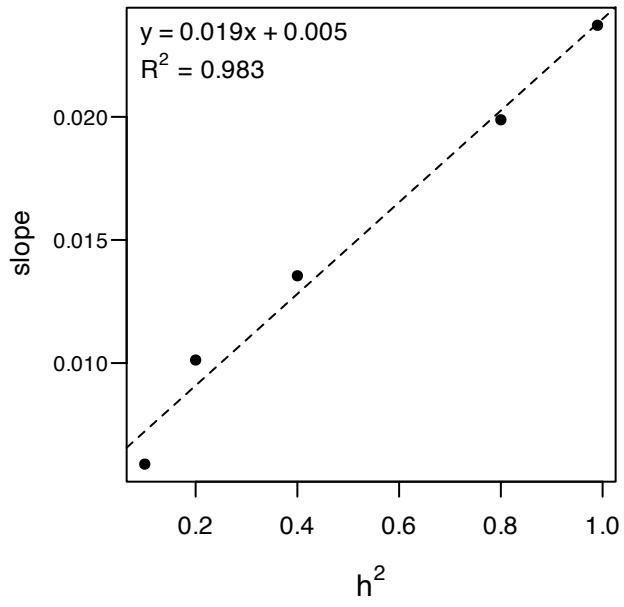**B**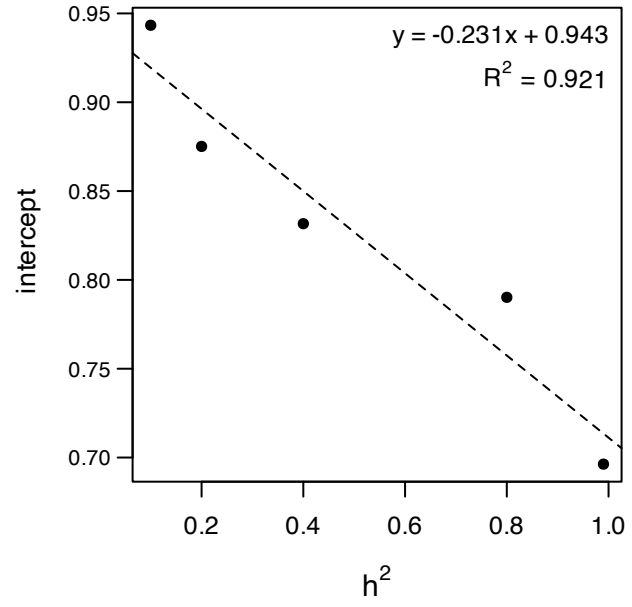

Supplement: S27 Fig — The A, slope and B, intercept terms from linear regressions of σ∆norm on √n plotted as a function of h2, demonstrating how standard error estimates deviate from expected precision as a function of the h2 of the examined phenotype. The coefficients and R2 of each linear regression are reported in the upper left of each panel. (PDF) [file pgen.1012037.s027.pdf]

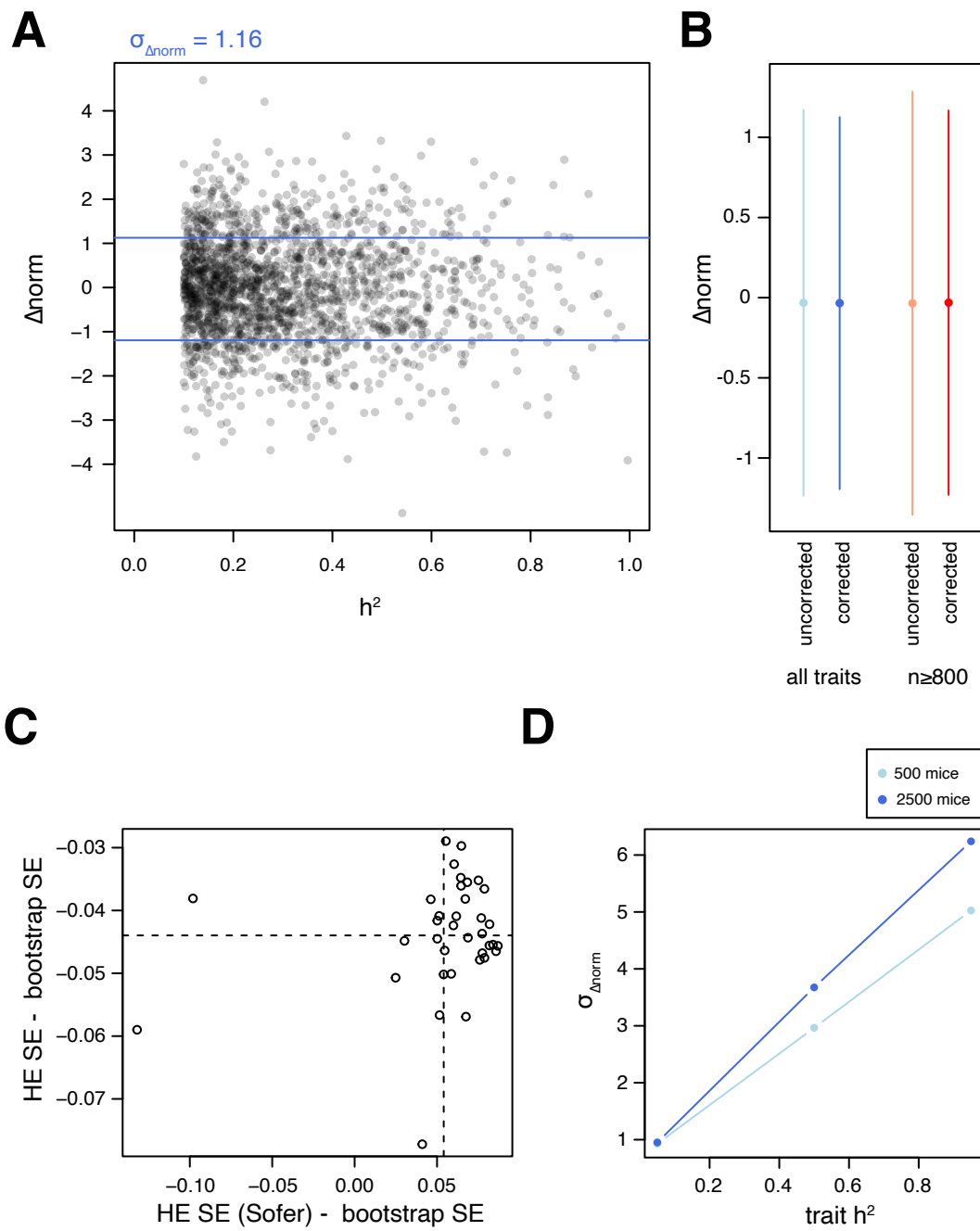

Supplement: S28 Fig — A, ∆norm values for 1,898 traits in DO mice using corrected standard error estimates are plotted as a function of trait h2. B, The mean and standard deviation of ∆norm values using corrected and uncorrected standard error estimates for all traits (left) and traits with larger sample sizes (right). C, Differences in the closed-form (y-axis) and analytic (x-axis) standard errors for h2 and the standard errors produced via bootstrapping as in [17]. Dashed lines indicated the mean difference among a set of 34 diverse traits measured in DO mice. D, Standard deviation of bootstrapped σ∆norm values for simulated phenotypes across a range of h2 and population sizes. (PDF) [file pgen.1012037.s028.pdf]
