# Supplementary material for: Genetic correlation-guided mega-analysis of DO mice provides mechanistic insight and candidate genes for age-related pathologies
Source: PLoS Genet. 2026 Feb 27;22(2):e1012037. doi: 10.1371/journal.pgen.1012037 (PMC12948109; doi:10.1371/journal.pgen.1012037)

Figure S12, Mullis et al, 2025

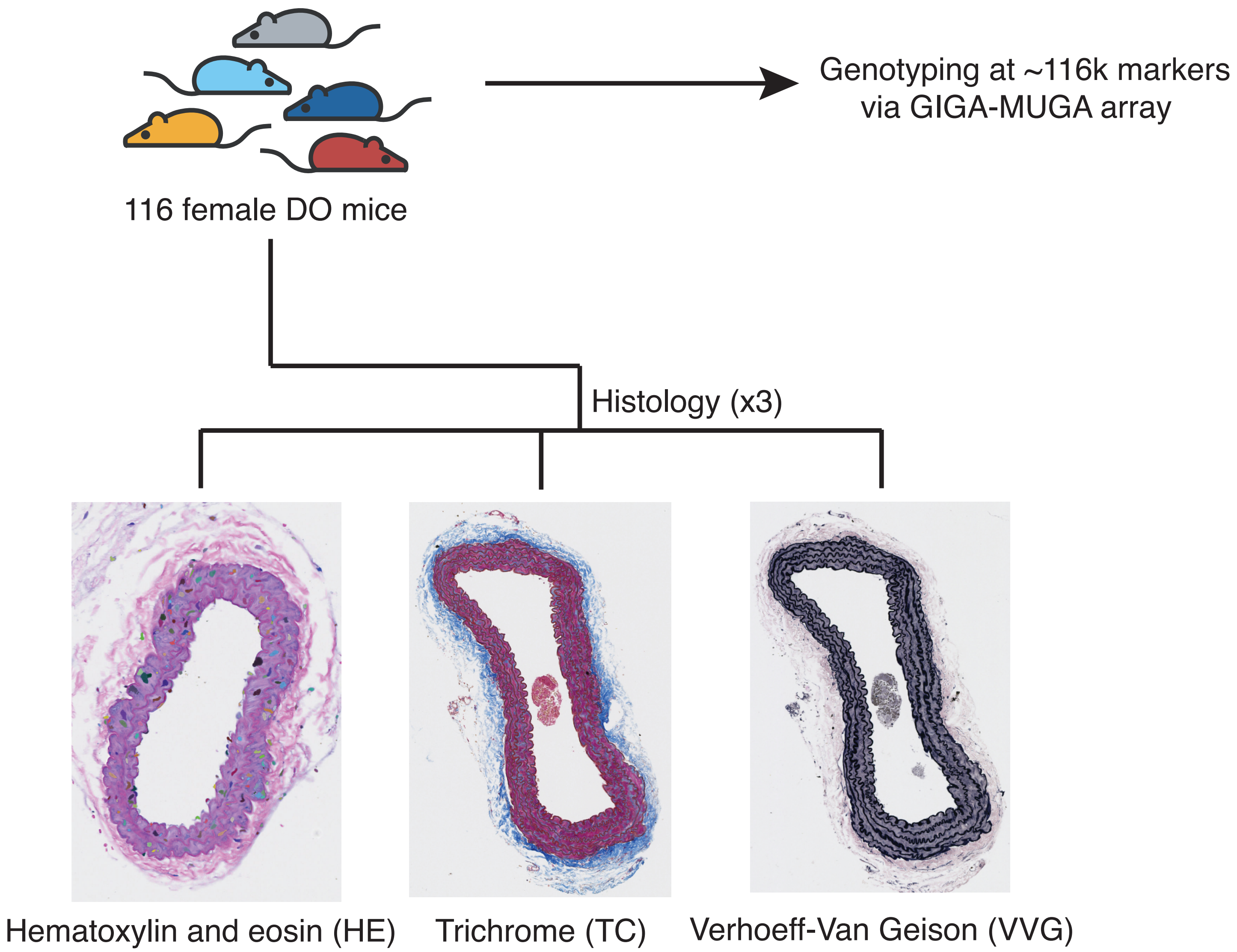

Supplement: S12 Fig — 116 DO mice were genotyped and their aortas were harvested. Tissues were sectioned, stained with HE, TC, or VVG, and imaged. (PDF) [file pgen.1012037.s012.pdf]
